# Multimodal signal dataset for 11 intuitive movement tasks from single upper extremity during multiple recording sessions

**DOI:** 10.1093/gigascience/giaa098

**Published:** 2020-10-07

**Authors:** Ji-Hoon Jeong, Jeong-Hyun Cho, Kyung-Hwan Shim, Byoung-Hee Kwon, Byeong-Hoo Lee, Do-Yeun Lee, Dae-Hyeok Lee, Seong-Whan Lee

**Affiliations:** Department of Brain and Cognitive Engineering, Korea University, 145 Anam-ro, Seongbuk-gu, Seoul 02841, South Korea; Department of Brain and Cognitive Engineering, Korea University, 145 Anam-ro, Seongbuk-gu, Seoul 02841, South Korea; Department of Brain and Cognitive Engineering, Korea University, 145 Anam-ro, Seongbuk-gu, Seoul 02841, South Korea; Department of Brain and Cognitive Engineering, Korea University, 145 Anam-ro, Seongbuk-gu, Seoul 02841, South Korea; Department of Brain and Cognitive Engineering, Korea University, 145 Anam-ro, Seongbuk-gu, Seoul 02841, South Korea; Department of Brain and Cognitive Engineering, Korea University, 145 Anam-ro, Seongbuk-gu, Seoul 02841, South Korea; Department of Brain and Cognitive Engineering, Korea University, 145 Anam-ro, Seongbuk-gu, Seoul 02841, South Korea; Department of Brain and Cognitive Engineering, Korea University, 145 Anam-ro, Seongbuk-gu, Seoul 02841, South Korea; Department of Artificial Intelligence, Korea University, 145 Anam-ro, Seongbuk-gu, Seoul 02841, South Korea

**Keywords:** brain–computer interface, multimodal signals, intuitive upper extremity movements, multiple sessions

## Abstract

**Background:**

Non-invasive brain–computer interfaces (BCIs) have been developed for realizing natural bi-directional interaction between users and external robotic systems. However, the communication between users and BCI systems through artificial matching is a critical issue. Recently, BCIs have been developed to adopt intuitive decoding, which is the key to solving several problems such as a small number of classes and manually matching BCI commands with device control. Unfortunately, the advances in this area have been slow owing to the lack of large and uniform datasets. This study provides a large intuitive dataset for 11 different upper extremity movement tasks obtained during multiple recording sessions. The dataset includes 60-channel electroencephalography, 7-channel electromyography, and 4-channel electro-oculography of 25 healthy participants collected over 3-day sessions for a total of 82,500 trials across all the participants.

**Findings:**

We validated our dataset via neurophysiological analysis. We observed clear sensorimotor de-/activation and spatial distribution related to real-movement and motor imagery, respectively. Furthermore, we demonstrated the consistency of the dataset by evaluating the classification performance of each session using a baseline machine learning method.

**Conclusions:**

The dataset includes the data of multiple recording sessions, various classes within the single upper extremity, and multimodal signals. This work can be used to (i) compare the brain activities associated with real movement and imagination, (ii) improve the decoding performance, and (iii) analyze the differences among recording sessions. Hence, this study, as a Data Note, has focused on collecting data required for further advances in the BCI technology.

## Data Description

### Background and purpose

The brain–computer interface (BCI) technology allows users to communicate with external devices including a speller [1], wheelchair [2], robotic arm [3–5], and robotic exoskeleton [6,7]. A non-invasive BCI commonly uses electroencephalography (EEG) signals to decode user intentions [8–11] because the EEG-based BCI system offers lower risk, lower cost, and more convenience than other non-invasive BCI paradigms (e.g., functional near-infrared spectroscopy [12]). EEG-based BCIs have been developed using various paradigms including motor imagery (MI) [13–15], steady-state visual evoked potential [16,17], event-related potential [18], and movement-related cortical potential [19,20]. Over the past decades, the information regarding the EEG datasets of these general paradigms has been published through competitions, cooperative projects, and open-access articles [21–25]. Some research groups have developed advanced machine learning algorithms and deep learning architectures for improving BCI performance using these datasets.

The recent advances in BCI systems have been focused on topics ranging from intuitive EEG decoding to directly matching the interaction between user intention and device feedback for real-world environments [20,26]. For example, to control a neuro-prosthetic arm using typical BCI paradigms, we temporarily matched BCI commands with robotic arm motions (e.g., MI for both hands = grasping motion of the robotic hand). However, this unintended artificial matching is hindered by several constraints, such as the small number of restrictive classes for communicating with devices and inflexible user training owing to unintuitive commands in real-world scenarios [7,13,27]. For example, if a robotic hand performs the grasping motion, the users should also imagine the hand-grasping motion the same as the robotic hand motion to enhance the natural decoding experience [27].

In this work, we collected data on intuitive upper extremity movements from 25 participants. To collect high-quality signal data, the experiments were conducted on healthy participants, who had maintained good physical condition by, e.g., limiting their alcohol intake and getting sufficient sleep. We focused on various upper extremity motions because they are the most extensible and available movements among all the body movements. Accordingly, we selected the upper extremities for decoding intuitive movements and then collected data based on the movement-based multimodal signals. The participants were asked to perform 11 different movement tasks: arm-reaching along 6 directions, hand-grasping of 3 objects, and wrist-twisting with 2 different motions. The corresponding 11 classes were designed for each segmented motion related to the arm, hand, and wrist, rather than for continuous limb movements. Therefore, the users of our dataset could either conduct respective analyses for individual classes or attempt decoding the complex upper extremity movements by combining data from different classes. For researchers focused on more advanced and analytical approaches using multimodal signals, the dataset comprised not only EEG data but also electromyography (EMG) and electro-oculography (EOG) data. These data were synchronously collected in the same experimental environment, while ensuring no unintentional interference between them. The data acquired using a 60-channel EEG, 7-channel EMG, and 4-channel EOG were simultaneously recorded during the experiment. EEG sensors were placed according to international specifications to collect signals from all the regions of the scalp. Additionally, EMG sensors were attached to carefully selected locations on the right arm to reflect the most relevant muscle activity information associated with the corresponding upper limb movement. We also recorded the EOG signals using 4 channels independent of the EEG channels to capture detailed eye movements, which were mainly used for artifact removal. The participants performed real upper extremity movements and MI associated with the 11 aforementioned motions. Additionally, each participant participated in 3 recording sessions at 1-week intervals and followed the same experimental protocols. To acquire a large amount of high-quality data, we prioritized the physical and mental condition of the participants during the experiments. Eventually, the multimodal signal dataset became sufficiently large for the BCI experiment because it now included data acquired from 82,500 trials performed for all the participants (i.e., 3,300 trials were collected per participant).

To the best of our knowledge, the present dataset descriptor is the first large public dataset for intuitive BCI paradigms to include multimodal signals such as EEG, EMG, and EOG signals. This study might contribute to the realization of reliable neurorehabilitation of patients with motor disabilities and a high-level BCI system for healthy users. Furthermore, to ensure the practicality of the BCI technology, we intend to investigate how to robustly decode motor-related intentions despite different recording sessions and subject dependency (i.e., the session-to-session problem [23,28] and subject independence problem [29]). However, at present only a few datasets exist that can be applied to various types of real-world applications and to develop a robust neural decoding model. To overcome this challenge, this study could contribute to the development of a practical BCI system based on deep learning techniques and multiple modalities by providing a large dataset.

### Experimental design

#### Participants

Twenty-five participants (all right-handed, aged 24–32 years, 15 men and 10 women) who were naive BCI users participated in the experiments. They were healthy individuals with no known neurophysiological anomalies or musculoskeletal disorders. Before the experiments, they were informed about the experimental protocols, paradigms, and purpose. After ensuring that they had understood the information, they provided their written consent according to the Declaration of Helsinki. The participants signed a form that agreed to the anonymous public release of their data. We checked their physical and mental states so that the influence of the BCI performance could be compared according to individual state. Additionally, each participant was required to be in normal health, get sufficient sleep (~8 h), and avoid alcohol, caffeinated drinks, and strenuous physical activity before the experiments. All the experimental protocols and environments were reviewed and approved by the Institutional Review Board (IRB) at Korea University (1040548-KU-IRB-17-181-A-2).

#### Environment

**Figure 1:**
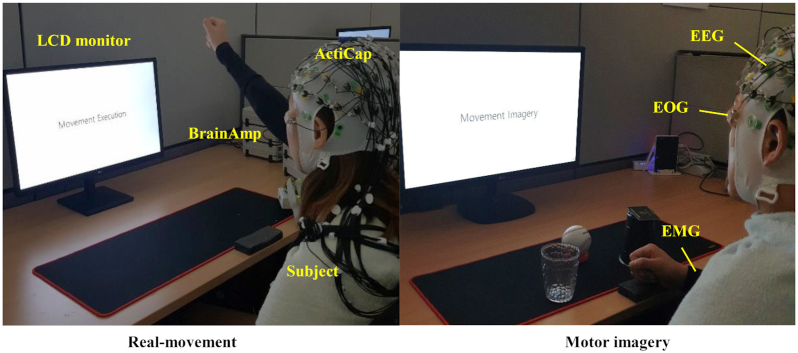
Experimental environments for acquiring multimodal signals related to the intuitive movement tasks. The participants were asked to perform real-movement (e.g., arm-reaching) and MI tasks (e.g., hand-grasping).

During the experiments, each participant was comfortably seated in a chair with armrests facing the front of an LCD monitor, ~80 ± 5 cm away from each other [30]. An EEG cap (Fig. 1) with 60 channels (actiCap, BrainProduct GmbH, Gilching, Bayern, Germany) was placed on the head of each participant. Surface EMG and EOG electrodes were attached to pre-assigned locations on the right arm and around the eyes of each participant, respectively. The participants were then asked to perform the movements with relaxed muscles and minimum eye and body movements during the data recording.

The duration of the experiment was ~6-7 h a day. Our experiment comprised multiple recording sessions (3 days) to consider inter-session and inter-participant variabilities. Compared with typical BCI experiments, our experiments required a longer recording time. To maintain the physical and mental condition of the participants and thus ensure high signal quality, the participants took sufficient breaks between each task. During the breaks, we first confirmed the physical and mental condition of the participants through self-report. If they reported any inconvenient position or unstable condition, we either adjusted the experimental environment according to their requests or halted the experiment. In the case the experiment was halted, the participants could ask to conduct the experiment next time or withdraw from the experiment altogether. However, if the conditions of the participants were good to conduct the experiment, we checked the impedances of the EEG, EMG, and EOG electrodes and injected electrolyte gel into them to maintain impedance values <15 kω. Thus, we attempted to obtain clear signals excluding spontaneous noise due to muscle and mental fatigue during the recording.

#### Experimental paradigm

**Figure 2:**
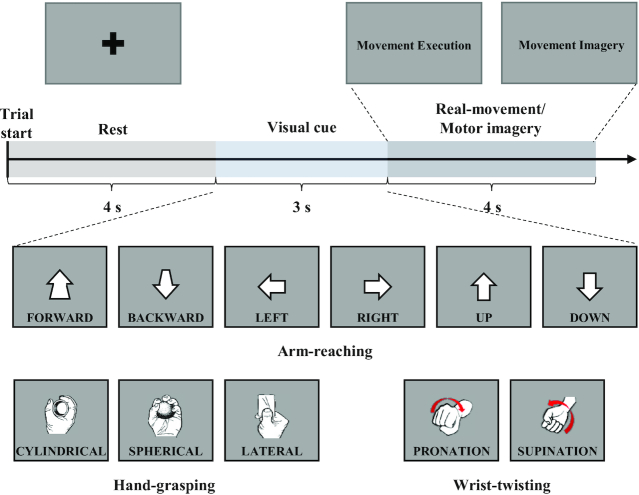
Experimental paradigm in a single trial and the representation of visual cues according to each task.

The experiment was designed to quantitatively acquire data related to the 11 different upper extremity movements for both real-movement and MI tasks. The participants conducted the experimental tasks using the same limbs. Decoding different tasks related to the same limb by using EEG signals could increase the number of possibilities of controlling the BCI system compared with typical somatosensory rhythm–based BCIs, which often only detected left/right hand and foot imagery [27]. The experimental tasks comprised 3 main upper extremity motions: arm-reaching, hand-grasping, and wrist-twisting. When the experiment began, visual instructions were provided on the monitor by displaying a black cross sign on a gray background. The participants stared at the visual instructions for 4 s while resting. After resting, a visual cue was displayed on the monitor with a text sign for 3 s, following which the participants began preparing to perform the real-movement or MI tasks according to the visual cue (see Fig. 2). Upon changing the visual cue to a text sign reading “Movement Execution” and “Movement Imagery,” the participants performed the corresponding tasks during 4 s. During the real-movement tasks, the participants were asked to focus on the sensations involved with each motion and to remember those sensations for the MI tasks.

**Figure 3:**
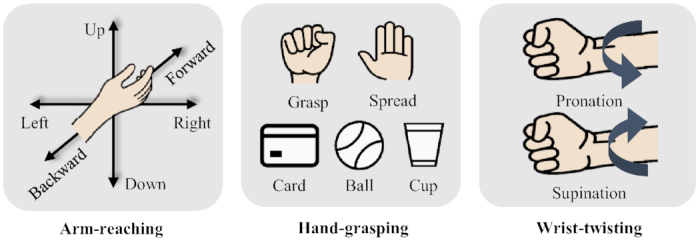
Experimental tasks of 11 intuitive upper extremity movements related to arm-reaching, hand-grasping, and wrist-twisting, respectively.

Arm-reaching along 6 directions: The participants were asked to perform multi-direction arm-reaching tasks directed from the center of their bodies outward. They performed the tasks along 6 different directions in 3D space: forward, backward, left, right, up, and down, as depicted in Fig. 3. In the real-movement tasks, the participants extended their arms along 1 of the directions. The arm-reaching paradigm required 50 trials along each direction so that data could be collected for a total of 300 trials. However, in the MI tasks, the participants only imagined performing an arm-reaching task; the number of trials in the MI paradigm was the same as in the real-movement paradigm.

Hand-grasping 3 objects: The participants were asked to grasp 3 objects of daily use via the corresponding grasping motions. They performed the 3 designated grasp motions by holding the objects, namely, card, ball, and cup, corresponding to lateral, spherical, and cylindrical grasp, respectively (see Fig. 3). In the real-movement tasks, we asked the participants to use their right hands to grasp a randomly selected object and hold it using its corresponding grasping motion. Eventually, we acquired data on 50 trials for each grasp, and hence, we collected 150 trials per participant. In the MI tasks, the participants performed only 1 of the 3 grasping motions per trial, randomly. The number of trials in the MI paradigm was the same as that in the real-movement paradigm.

Wrist-twisting with 2 different motions: For the wrist-twisting tasks, the participants rotated their wrists to the left (pronation) and right (supination), as depicted in Fig. 3. During real-movement task, each participant maintained his/her right hand in a neutral position with the elbow comfortably placed on the desk. Notably, wrist pronation and supination are complex actions used to decode user intentions from brain signals. Additionally, these movements are intuitive motions for realizing neurorehabilitation and prosthetic control [31]. We collected data for 50 trials per motion (i.e., total 100 trials) per day, and the visual cues were randomly displayed.

Additionally, the participants were asked to participate in 3 recording sessions with a 1-week interval between each session. The experimental environment and protocols were the same for all 3 sessions. Consequently, we collected data from 3,300 trials (1,800 trials for arm-reaching, 900 for hand-grasping, and 600 for wrist-twisting) in all classes per participant, for both real-movement and MI paradigms.

### Data records

We simultaneously collected 3 different kinds of physiological signals, namely, EEG, EMG, and EOG signals for 11 different upper extremity movements (see Fig. 3). During the experiment, the signals were acquired using the same digital amplifier and types of electrodes. Therefore, the raw signals were stored together in 1 data file according to each participant. To obtain high-quality signals, the impedances of all the channels were maintained to be <15 kω. After applying conductive gel to the electrodes, we validated the accuracy of the EEG and EOG signals by asking the participants to blink and close their eyes. The eye-blinking task was used to identify strong spikes in the frontal EEG channels (e.g., Fp1 and Fp2) and 4 EOG channels. The eye-closing task was used to confirm the α oscillations in the occipital channels (e.g., O1, O2, and Oz). We also asked the participants to perform a simple hand-grasping motion to confirm the strong spikes in the EMG signals.

#### EEG signals

**Figure 4:**
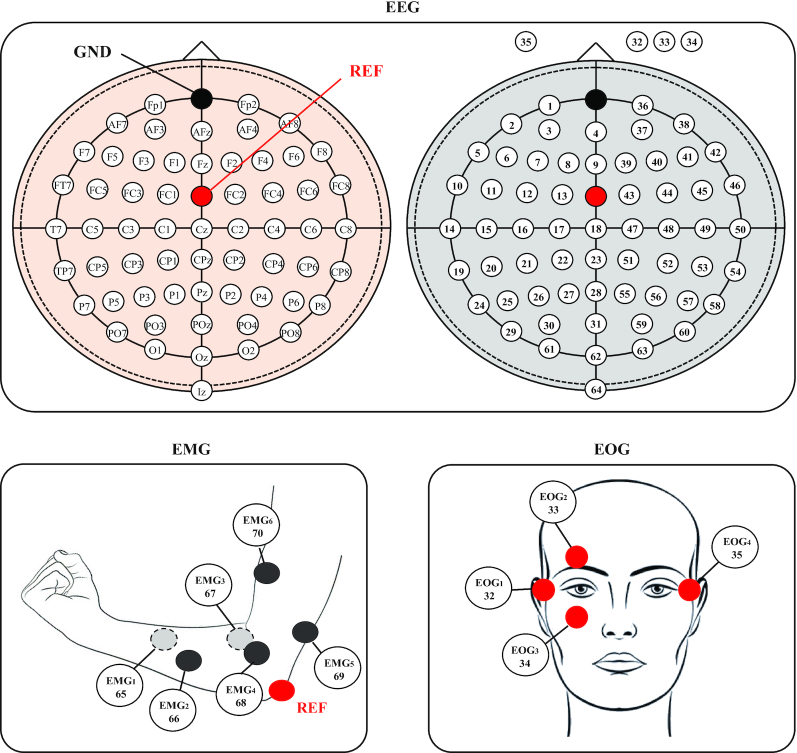
Data configuration for 60 EEG, 7 EMG, and 4 EOG channels. Specifically, 4 EOG channels were numbered as 32–35 out of the 64-channel actiCAP montage.

The EEG data were recorded in conjunction with an EEG signal amplifier (BrainAmp, BrainProduct GmbH, Germany), sampled at 2,500 Hz. Additionally, we applied a 60 Hz with a notch filter to reduce the effect of external electrical noises (e.g., DC noise due to power supply, scan rate of the monitor display, and frequency of the fluorescent lamp) in raw signals [21,32,33]. The raw data were recorded using BrainVision (BrainProduct GmbH, Germany) with MATLAB 2019a (MathWorks Inc., USA). Furthermore, a toal of 60 EEG electrodes were selected by following a 10-20 international configuration (Fp1-2, AF5-6, AF7-8, AFz, F1-8, Fz, FT7-8, FC1-6, T7-8, C1-6, Cz, TP7-8, CP1-6, CPz, P1-8, Pz, PO3-4, PO7-8, POz, O1-2, Oz, and Iz). Ground and reference channels were placed on the Fpz and FCz, respectively (see Fig. 4). The impedances of all the electrodes between the sensors and scalp skin were maintained to be <15 kω. During breaks, conductive gel was injected into the electrodes using a syringe with a blunt needle.

#### EMG signals

The EMG signals were recorded using 7 silver/silver chloride electrodes from the digital amplifier, the same equipment used to record the EEG signals. We simultaneously acquired the EMG and EEG signals using the same amplifer [34]. The signals were captured at a sampling rate of 2,500 Hz with a 60 Hz notch filter, the same as the setting used to record the EEG signals. The EMG data were recorded from 6 related muscles for right arm movement: extensor carpi ulnaris, extensor digitorum, flexor carpi radialis, flexor carpi ulnaris, biceps brachii, and triceps brachii (see Fig. 4) [35]. The ground and reference were recorded in Fpz and FCz, respectively, which are the same as the EEG and EOG signals. The last electrode was placed on the elbow of the right arm, which is a non–muscle movement area, as an alternative reference signal [36]. The purpose of recording EMG signals was to detect muscle activities when the participants performed the designated tasks. The signals could prove that the participants performed MI tasks without muscle movement. Simultaneously, the electrodes were placed so as to record a sufficient number of signals from various arm and hand movements (i.e., 6 arm-reaching, 3 hand-grasping, and 2 wrist-twisting motions).

#### EOG signals

The EOG signals were recorded using 4 channels while following the same protocol. Subsequently, the FT9, FT10, TP9, and TP10 electrodes were moved to the region around the eyes to function as EOG channels to eliminate artifacts due to ocular activities. One of these channels was moved to the region around the left eye and the others to the region around the right eye (see Fig. 4). The electrodes EOG_1_ and EOG_4_ were used to record horizontal eye movements, while EOG_2_ and EOG_3_ were used to record vertical movements [37]. Medical tape was used to hold the sensors around the eyes and maintain the impedances of all the electrodes to be <15 kω.

#### Data format and structure

Readers can access our codes and datasets through the GigaDB repository. The “Read me”.pdf file, which overviews of data description and code execution, is included in the repository. Several useful scripts, including Data*_*analysis.m and Visualization.m files, are in the “SampleCode” folder. We recommend the BBCI (http://www.bci.de) toolbox [38] in the “Reference toolbox” folder and the “Signal Processing” toolbox in the MATLAB software, for data processing using our custom code. Please directly contact the authors for more information on the code script.

Each dataset (raw signals, converted data, and scripts) is also publicly available via the GigaDB repository. The raw signals and converted data are contained in the corresponding folders, namely, “Raw data” and “Converted data,” respectively. Table 1 summarizes the data description for both folders. The indicated "Task" includes the names of arm-reaching, multigrasp, and twisting. We provide the folders, namely, “Raw data” and “Converted data,” which comprise .eeg, .vmrk, .vhdr, and .mat files for each participant. The .eeg file includes the raw EEG, EOG, and EMG signal data because of the simultaneous data acquisition performed using the same amplifier. Moreover, the .vmrk file provides the marked trigger information (e.g., trigger number, marked time, and file name) and the .vhdr file includes the number of channels, sampling rate, channel position, and electrode impedances. The .mat file includes pre-processed EEG, EOG, and EMG data, channels, class information, scalp montage, and sample frequency. Additionally, to enable easy data access for the users, we provided the dataset after converting the .eeg file to a .mat file in the “Converted data” folder. The converted .mat file includes some information such as trigger mark information, channel configuration, and epoch segmentation.

**Table 1:** Data description for Raw data folder and Converted data folder

Raw data		Converted data (e.g., session1_sub1_multigrasp_MI.mat)		
Name	Description	Name	Suffix	Description
session1_sub1_(Task)_(realMove/MI).eeg	Raw signals (session 1)	.dat	.x	Pre-processed data
session1_sub1_(Task)_(realMove/MI).vhdr	Data header information (session 1)		.fs	Sampling frequency
session1_sub1_(Task)_(realMove/MI).vmrk	Marker information (session 1)		.file	File name
			.clab	Channel information
		.mnt	.x	X coordinates for channel position
session2_sub1_(Task)_(realMove/MI).eeg	Raw signals (session 2)		.y	Y coordinates for channel position
session2_sub1_(Task)_(realMove/MI).vhdr	Data header information (session 2)		.pos_3d	3D coordinates for channel position
session2_sub1_(Task)_(realMove/MI).vmrk	Marker information (session 2)		.clab	Channel information
		.mrk	.pos	Trigger marking time
			.toe	Trigger number
session3_sub1_(Task)_(realMove/MI).eeg	Raw signals (session 3)		.fs	Sampling frequency
session3_sub1_(Task)_(realMove/MI).vhdr	Data header information (session 3)		.y	Class labels
session3_sub1_(Task)_(realMove/MI).vmrk	Marker information (session 3)		.className	Class name
			.mics	Experiment start and end information

Data_analysis.m provides the basic data-processing script, which includes data loading, signal pre-processing, artifact rejection, feature extraction, classification, and performance evaluation. All users can download and unzip the SampleData.zip file contained in the “SampleData” folder before executing each code.

Visualization.m enables the visualization of raw signals, scalp distribution, and event-related spectral perturbation (ERSP) using EEGLAB [39]. The raw signals were visualized as channels through the time representation for the representative participant No. 8. The scalp plot can be visualized to choose a specific channel and time epoch for a selected participant. The ERSP plot requires the installation of the EEGLAB toolbox. After installing the EEGLAB toolbox, users can load the .vhdr file to EEGLAB and visualize the ERSP pattern that follows the description.

## Data Validation

### Methods

The technical signal validation was conducted using a BBCI toolbox [38] in the MATLAB 2019a environment. The initial settings of the recording program for converting an analog signal into a digital one were slightly different for each signal because of the scale of the signal amplitude. For each of the EEG, EMG, and EOG signals, triggers were marked to indicate the experimental state.

**Figure 5:**
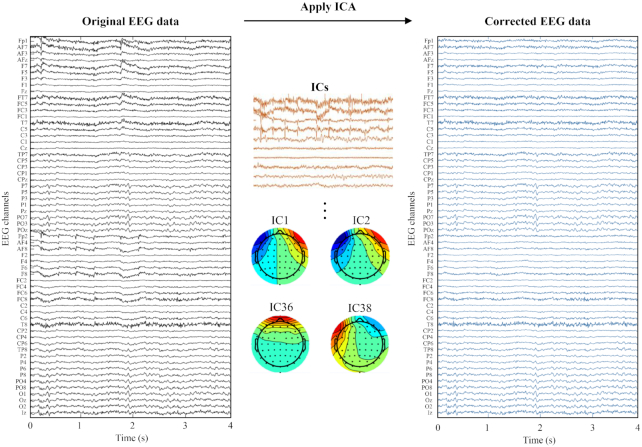
Infomax ICA based on EOG data was applied to eliminate the eye movement–induced noise from the original EEG data. The right panel shows the corrected EEG data wherein noise has been eliminated by the application of the infomax ICA to the original EEG data.

Initially, for data pre-processing, we applied a zero-phase fourth-order Butterworth filter for band-pass signal filtering in the EEG signals. The data were filtered between 8 and 30 Hz (μ and β bands, respectively), known to be within the motor-related frequency range, and this could also include the spectral range for somatosensory rhythm observation (i.e., 13–15 Hz) [40,41]. For artifact rejection, the apparent eye-blinking contamination in the EEG signal was removed via independent component analysis (ICA) [42]. To obtain corrected EEG data, we removed the contamination factors using the infomax ICA [43], which is used to decompose brain signals into statistically independent components (ICs). From various types of ICA methods, we adopted the ICA with the infomax algorithm because it could robustly remove artifacts, such as eye and head movement artifacts, from the EEG data [44]. The EEG data were transformed by the ICA mixing matrix. The contaminated ICs with patterns similar to the EOG channels (i.e., horizontal and vertical eye movements) were removed. Subsequently, the remaining ICs were projected back into the scalp channel space to be reconstructed as the corrected EEG data (see Fig. 5). Before feature extraction, we segmented a time interval of −0.5 to 4 s for performing EEG classification and also selected a baseline period as −0.5 to 0 s [25].

For evaluating the classification performance using EEG signals, we adopted the common spatial pattern (CSP) algorithm as a feature extraction method and a regularized linear discriminant analysis (RLDA) method as the classification method. CSP feature extraction method and RLDA classifier are generally used as baseline algorithms for decoding EEG-based MI and motor execution in the field of BCI [26,45,46]. Especially, CSP, one of the EEG feature extraction methods, is proved to be robust in spatial feature extraction for decoding movement-related tasks and MI. CSP trained for finding the optimal spatial filter that maximizes inter-class variation and minimizes intra-class variation. We calculated a transformation matrix using CSP in which the logarithmic variances of the first and last 3 columns were used as a feature. We trained the RLDA classifier by adding a regularization term to the covariance matrix using the optimal shrinkage parameters [47,48]. Essentially, shrinkage performs regularization to improve the estimation of covariance matrices where the training samples are fewer than features. Therefore, during the training period, the optimal shrinkage parameter was automatically estimated with the maximum covariance between classes. In the classification procedure, we classified the multi-class according to each different experimental task, separately, such as arm-reaching along 6 directions (6-class), hand-grasping of 3 objects (3-class), and wrist-twisting with 2 different motions (2-class), as depicted in Fig. 3. We applied 10 × 10-fold cross-validation for fair performance measurement so that we partitioned the data samples as equal sizes into 10 subsets. One of the subsets was selected as the test dataset and the remaining others as the training datasets. The cross-validation process was conducted 10 times, with each of the 10 subsets used once as the test dataset to avoid variability problems in performance evaluation. The evaluation was estimated using all recorded classes simultaneously over all the recording sessions. Table 2 presents the averaged evaluation results obtained by estimating the classification performance of each arm-reaching (6-class), hand-grasping (3-class), and wrist-twisting (2-class) task.

**Table 2:** Classification accuracy for each task across all the participants during multiple recording sessions

Participant No.	Session 1 mean (±SD)						Session 2 mean (±SD)						Session 3 mean (±SD)					
	Real movement			Motor imagery			Real movement			Motor imagery			Real movement			Motor imagery		
	Reach	Grasp	Twist	Reach	Grasp	Twist	Reach	Grasp	Twist	Reach	Grasp	Twist	Reach	Grasp	Twist	Reach	Grasp	Twist
1	0.21	0.40	0.61	0.21	0.42	0.60	0.21	0.41	0.56	0.21	0.42	0.58	0.24	0.43	0.58	0.21	0.40	0.57
	(±0.01)	(±0.02)	(±0.03)	(±0.02)	(±0.04)	(±0.04)	(±0.02)	(±0.03)	(±0.03)	(±0.01)	(±0.04)	(±0.03)	(±0.01)	(±0.03)	(±0.03)	(±0.02)	(±0.03)	(±0.03)
2	0.24	0.41	0.56	0.22	0.40	0.63	0.23	0.41	0.63	0.23	0.40	0.56	0.20	0.41	0.66	0.21	0.40	0.60
	(±0.01)	(±0.03)	(±0.04)	(±0.01)	(±0.02)	(±0.02)	(±0.02)	(±0.03)	(±0.03)	(±0.01)	(±0.02)	(±0.04)	(±0.03)	(±0.02)	(±0.02)	(±0.02)	(±0.02)	(±0.04)
3	0.18	0.42	0.59	0.20	0.39	0.61	0.19	0.40	0.59	0.20	0.38	0.63	0.20	0.41	0.56	0.19	0.36	0.55
	(±0.02)	(±0.02)	(±0.04)	(±0.01)	(±0.04)	(±0.02)	(±0.02)	(±0.03)	(±0.03)	(±0.02)	(±0.03)	(±0.05)	(±0.01)	(±0.03)	(±0.04)	(±0.02)	(±0.02)	(±0.05)
4	0.17	0.40	0.64	0.21	0.39	0.60	0.28	0.39	0.59	0.18	0.38	0.59	0.21	0.41	0.57	0.20	0.40	0.54
	(±0.02)	(±0.05)	(±0.03)	(±0.02)	(±0.03)	(±0.05)	(±0.02)	(±0.01)	(±0.03)	(±0.02)	(±0.03)	(±0.03)	(±0.01)	(±0.02)	(±0.05)	(±0.02)	(±0.02)	(±0.03)
5	0.21	0.39	0.59	0.20	0.43	0.55	0.20	0.41	0.54	0.18	0.35	0.56	0.19	0.41	0.55	0.22	0.35	0.58
	(±0.01)	(±0.03)	(±0.02)	(±0.01)	(±0.03)	(±0.03)	(±0.02)	(±0.03)	(±0.04)	(±0.01)	(±0.03)	(±0.02)	(±0.02)	(±0.02)	(±0.06)	(±0.01)	(±0.03)	(±0.02)
6	0.19	0.41	0.60	0.19	0.46	0.55	0.22	0.43	0.60	0.24	0.44	0.60	0.24	0.47	0.62	0.21	0.46	0.54
	(±0.02)	(±0.03)	(±0.04)	(±0.02)	(±0.04)	(±0.04)	(±0.02)	(±0.04)	(±0.03)	(±0.01)	(±0.03)	(±0.03)	(±0.02)	(±0.03)	(±0.05)	(±0.01)	(±0.02)	(±0.03)
7	0.22	0.38	0.61	0.24	0.53	0.63	0.21	0.53	0.60	0.27	0.60	0.63	0.21	0.65	0.57	0.34	0.53	0.59
	(±0.01)	(±0.03)	(±0.03)	(±0.02)	(±0.03)	(±0.03)	(±0.02)	(±0.03)	(±0.02)	(±0.02)	(±0.02)	(±0.03)	(±0.02)	(±0.03)	(±0.04)	(±0.02)	(±0.03)	(±0.03)
8	0.20	0.50	0.59	0.21	0.54	0.65	0.22	0.66	0.53	0.19	0.79	0.63	0.22	0.65	0.61	0.19	0.75	0.59
	(±0.01)	(±0.03)	(±0.03)	(±0.02)	(±0.03)	(±0.04)	(±0.02)	(±0.03)	(±0.03)	(±0.02)	(±0.03)	(±0.04)	(±0.02)	(±0.02)	(±0.05)	(±0.01)	(±0.02)	(±0.04)
9	0.20	0.38	0.57	0.25	0.35	0.56	0.21	0.38	0.62	0.21	0.39	0.60	0.20	0.41	0.58	0.19	0.39	0.59
	(±0.01)	(±0.04)	(±0.02)	(±0.01)	(±0.03)	(±0.02)	(±0.02)	(±0.03)	(±0.05)	(±0.02)	(±0.03)	(±0.04)	(±0.02)	(±0.03)	(±0.04)	(±0.01)	(±0.05)	(±0.05)
10	0.20	0.43	0.58	0.21	0.41	0.55	0.22	0.51	0.60	0.18	0.48	0.60	0.22	0.39	0.58	0.19	0.37	0.62
	(±0.02)	(±0.02)	(±0.05)	(±0.02)	(±0.02)	(±0.02)	(±0.01)	(±0.04)	(±0.04)	(±0.02)	(±0.04)	(±0.03)	(±0.01)	(±0.03)	(±0.05)	(±0.02)	(±0.02)	(±0.03)
11	0.25	0.43	0.58	0.35	0.41	0.62	0.24	0.52	0.58	0.26	0.40	0.64	0.27	0.64	0.58	0.33	0.59	0.56
	(±0.02)	(±0.02)	(±0.05)	(±0.01)	(±0.03)	(±0.03)	(±0.02)	(±0.03)	(±0.04)	(±0.02)	(±0.02)	(±0.02)	(±0.02)	(±0.02)	(±0.05)	(±0.02)	(±0.02)	(±0.02)
12	0.21	0.77	0.56	0.21	0.93	0.55	0.22	0.89	0.56	0.21	0.94	0.63	0.21	0.58	0.58	0.21	0.56	0.65
	(±0.02)	(±0.02)	(±0.02)	(±0.01)	(±0.02)	(±0.04)	(±0.01)	(±0.02)	(±0.03)	(±0.01)	(±0.02)	(±0.03)	(±0.02)	(±0.02)	(±0.04)	(±0.02)	(±0.02)	(±0.03)
13	0.20	0.40	0.58	0.19	0.44	0.65	0.22	0.37	0.61	0.21	0.35	0.59	0.19	0.43	0.56	0.17	0.37	0.57
	(±0.01)	(±0.03)	(±0.06)	(±0.02)	(±0.04)	(±0.03)	(±0.02)	(±0.02)	(±0.04)	(±0.02)	(±0.03)	(±0.03)	(±0.02)	(±0.02)	(±0.04)	(±0.02)	(±0.04)	(±0.03)
14	0.22	0.43	0.52	0.21	0.46	0.56	0.23	0.69	0.54	0.21	0.42	0.55	0.21	0.50	0.56	0.18	0.45	0.54
	(±0.02)	(±0.03)	(±0.01)	(±0.02)	(±0.03)	(±0.05)	(±0.02)	(±0.02)	(±0.02)	(±0.02)	(±0.03)	(±0.03)	(±0.01)	(±0.02)	(±0.02)	(±0.02)	(±0.02)	(±0.03)
15	0.21	0.38	0.61	0.19	0.40	0.58	0.21	0.51	0.63	0.18	0.43	0.57	0.20	0.46	0.61	0.20	0.41	0.63
	(±0.01)	(±0.04)	(±0.03)	(±0.02)	(±0.04)	(±0.04)	(±0.02)	(±0.02)	(±0.03)	(±0.01)	(±0.03)	(±0.05)	(±0.02)	(±0.03)	(±0.04)	(±0.02)	(±0.02)	(±0.04)
16	0.21	0.40	0.63	0.23	0.43	0.59	0.20	0.43	0.59	0.20	0.36	0.51	0.19	0.43	0.55	0.20	0.37	0.65
	(±0.02)	(±0.03)	(±0.04)	(±0.02)	(±0.02)	(±0.03)	(±0.01)	(±0.04)	(±0.03)	(±0.02)	(±0.03)	(±0.03)	(±0.03)	(±0.03)	(±0.04)	(±0.02)	(±0.04)	(±0.02)
17	0.25	0.86	0.56	0.20	0.34	0.59	0.23	0.86	0.65	0.21	0.36	0.62	0.27	0.41	0.56	0.17	0.39	0.52
	(±0.02)	(±0.03)	(±0.04)	(±0.01)	(±0.03)	(±0.04)	(±0.01)	(±0.03)	(±0.03)	(±0.01)	(±0.03)	(±0.03)	(±0.01)	(±0.03)	(±0.04)	(±0.02)	(±0.04)	(±0.04)
18	0.22	0.47	0.64	0.22	0.36	0.63	0.21	0.70	0.64	0.22	0.50	0.63	0.24	0.52	0.58	0.20	0.37	0.57
	(±0.01)	(±0.02)	(±0.03)	(±0.01)	(±0.02)	(±0.05)	(±0.01)	(±0.03)	(±0.03)	(±0.02)	(±0.03)	(±0.04)	(±0.02)	(±0.03)	(±0.03)	(±0.02)	(±0.01)	(±0.02)
19	0.22	0.72	0.61	0.22	0.39	0.62	0.22	0.66	0.50	0.18	0.40	0.61	0.19	0.77	0.64	0.21	0.40	0.54
	(±0.02)	(±0.02)	(±0.03)	(±0.02)	(±0.03)	(±0.03)	(±0.02)	(±0.02)	(±0.03)	(±0.01)	(±0.03)	(±0.02)	(±0.02)	(±0.03)	(±0.03)	(±0.02)	(±0.02)	(±0.03)
20	0.43	0.38	0.56	0.19	0.41	0.58	0.35	0.60	0.55	0.21	0.41	0.59	0.29	0.42	0.61	0.20	0.40	0.54
	(±0.01)	(±0.02)	(±0.04)	(±0.02)	(±0.03)	(±0.03)	(±0.02)	(±0.03)	(±0.03)	(±0.01)	(±0.02)	(±0.04)	(±0.02)	(±0.03)	(±0.03)	(±0.02)	(±0.02)	(±0.04)
21	0.28	0.45	0.55	0.20	0.43	0.60	0.22	0.45	0.56	0.21	0.46	0.53	0.29	0.53	0.58	0.19	0.40	0.58
	(±0.02)	(±0.04)	(±0.05)	(±0.02)	(±0.02)	(±0.02)	(±0.02)	(±0.04)	(±0.03)	(±0.02)	(±0.03)	(±0.02)	(±0.02)	(±0.02)	(±0.04)	(±0.02)	(±0.01)	(±0.02)
22	0.29	0.73	0.61	0.23	0.46	0.60	0.24	0.46	0.55	0.21	0.36	0.61	0.23	0.60	0.55	0.21	0.42	0.55
	(±0.02)	(±0.02)	(±0.05)	(±0.02)	(±0.03)	(±0.03)	(±0.02)	(±0.02)	(±0.03)	(±0.02)	(±0.03)	(±0.03)	(±0.01)	(±0.03)	(±0.03)	(±0.02)	(±0.02)	(±0.03)
23	0.19	0.45	0.60	0.21	0.42	0.57	0.25	0.36	0.60	0.20	0.38	0.57	0.22	0.52	0.60	0.21	0.36	0.57
	(±0.01)	(±0.03)	(±0.04)	(±0.02)	(±0.03)	(±0.04)	(±0.01)	(±0.03)	(±0.04)	(±0.02)	(±0.02)	(±0.04)	(±0.01)	(±0.03)	(±0.03)	(±0.01)	(±0.03)	(±0.03)
24	0.20	0.35	0.53	0.23	0.43	0.61	0.21	0.47	0.59	0.20	0.47	0.58	0.22	0.57	0.51	0.22	0.35	0.56
	(±0.01)	(±0.03)	(±0.02)	(±0.01)	(±0.03)	(±0.04)	(±0.01)	(±0.04)	(±0.02)	(±0.02)	(±0.03)	(±0.03)	(±0.01)	(±0.03)	(±0.04)	(±0.03)	(±0.03)	(±0.01)
25	0.24	0.49	0.57	0.19	0.41	0.61	0.22	0.64	0.57	0.20	0.41	0.58	0.24	0.58	0.60	0.21	0.42	0.60
	(±0.01)	(±0.04)	(±0.04)	(±0.01)	(±0.04)	(±0.04)	(±0.02)	(±0.03)	(±0.04)	(±0.01)	(±0.02)	(±0.04)	(±0.01)	(±0.03)	(±0.04)	(±0.01)	(±0.03)	(±0.03)
Mean	0.23	0.47	0.59	0.22	0.44	0.60	0.23	0.53	0.58	0.21	0.45	0.59	0.22	0.50	0.58	0.21	0.43	0.58
	(±0.05)	(±0.14)	(±0.03)	(±0.03)	(±0.11)	(±0.03)	(±0.03)	(±0.15)	(±0.04)	(±0.02)	(±0.14)	(±0.03)	(±0.03)	(±0.10)	(±0.03)	(±0.04)	(±0.09)	(±0.03)

The evaluation was estimated from each upper extremity motion such as arm-reaching (6-class), hand-grasping (3-class), and wrist-twisting (2-class) in real movement and MI.

**Figure 6:**
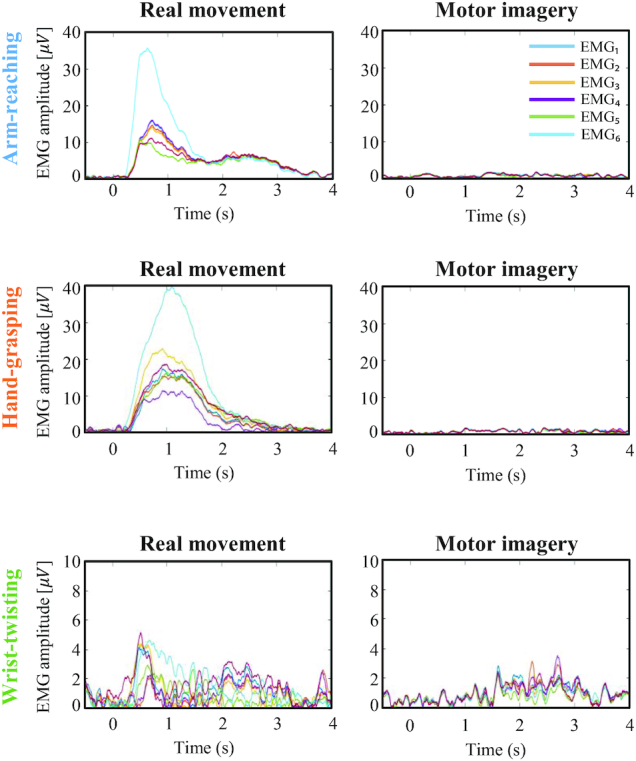
Example representation of EMG activation according to each 6-channel EMG. From the top, the plot represents the activities of the EMG signals as the representative participant No. 4 performs arm-reaching, hand-grasping, and wrist-twisting tasks, respectively.

Furthermore, we checked the EMG activation to verify whether the upper extremity movement was performed or not according to the tasks. In this work, the EMG signals were pre-processed from 10 to 500 Hz with a Butterworth fifth-order zero-phase band-pass filter [34, 49]. We segmented a time interval of −0.5 to −4 s for EMG data analysis. We selected the interval of −0.5 to 0 s as the baseline period. Subsequently, the data were rectified using the absolute values, following which we calculated the moving average of EMG amplitudes with a 100-ms interval. The EMG signals could show how well the participants had followed the experimental protocol for each task. For example, if the participant was asked to perform the MI task, the EMG activation should not show a peak shape because of the static state at that time, as depicted in Fig. 6. EMG signal patterns contain important information regarding muscle activation and noise. For example, the noise due to heartbeat and other unrelated movements reduces the quality of EMG signals. While the participants performed the wrist-twisting task, the amplitude scale was reduced as compared with other work, so that noise information could be confirmed to be displayed. Meanwhile, in the real-movement tasks, the EMG signals could show the activation while the participants were performing the upper extremity movement task. Notably, the signal amplitude of channel EMG_6_ is higher than those of the other channels. Because the biceps are relatively bigger than other muscles, a peak phenomenon occurred [50]. Particularly, biceps are used the most in bending the arm. The EMG signals featured a large signal amplitude because more muscle activity increases the signal amplitude. Consequently, the EMG signal amplitude was large for the arm-reaching and hand-grasping tasks but small for the wrist-twisting task [51].

### Results and Discussion

We verified the data through the EEG-based classification results, the EMG signal quality, and spectral and spatial EEG presentations. First, we verified the data on the basis of EMG signal quality, as shown in Fig. 6. Using the EMG signals recorded while the participants performed each arm-reaching, hand-grasping, and wrist-twisting experiment, we could confirm the quality of the data obtained. Furthermore, dynamic EMG signals were observed during the real-movement sessions; additionally insignificant changes were observed in the EMG signals close to the rest state in the MI sessions. The EMG signals, particularly, proved that we had collected the data appropriately because they were not activated throughout the entire duration of the actual motion session, and appeared strongly for the actual movements of the participants right after ~0.5 seconds from the onset. Additionally, the non-activated EMG signals during the MI tasks clearly indicate that the movement artifacts of the corresponding EEG signal were minimized.

**Figure 7:**
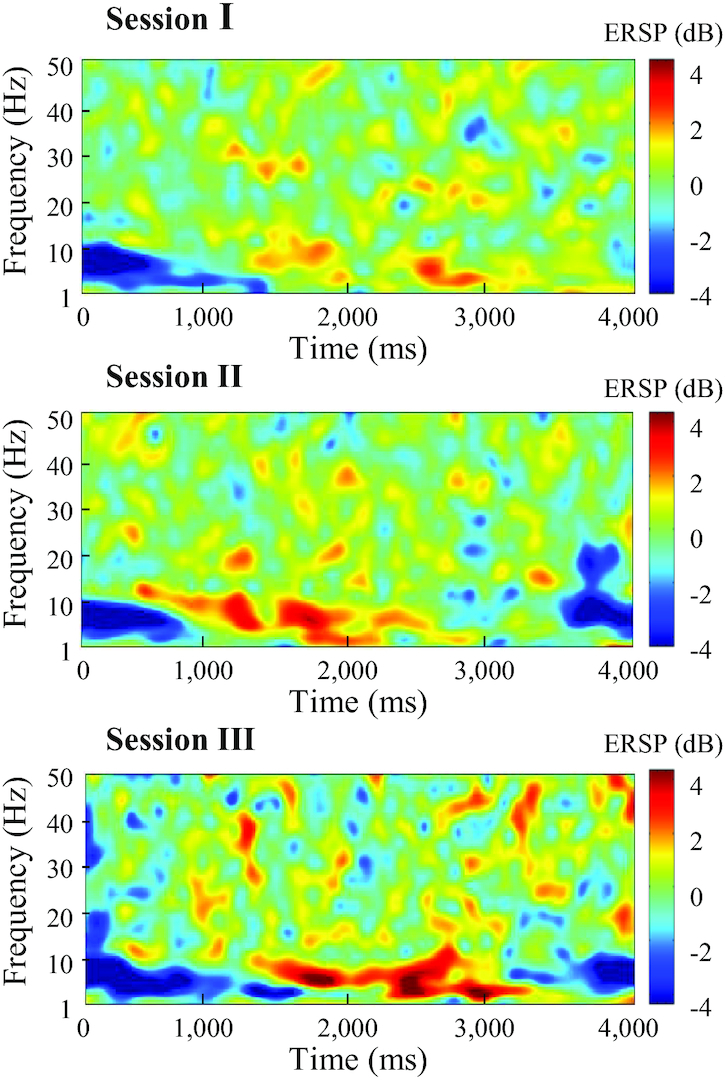
EEG data validation via spectral representation. The event-related desynchronization/synchronization (ERD/ERS) representation of channel C3 during multiple recording sessions.

We also analyzed the EEG signals in the spectral and spatial domains to confirm the data quality. Fig. 7 shows the examples of spectral energy information in the EEG data for a representative participant during multiple recording sessions. The ERSP plot illustrates spectral variability according to the time epoch in a certain channel (C3). Generally, the ERSP plot showed ERD/ERS patterns, which reflect sensorimotor activation and deactivation, respectively. The ERD patterns can be seen during motor preparation, execution, and imagery as correlations in an activated cortical region. ERS can be observed after the imagery or execution of movement over the same region [52,53]. In Fig. 7, ERD/ERS patterns appeared during all the imagery phases (0–4 s), showing the same cortical activation on the μ band across all the recording sessions. Additionally, we conducted a statistical analysis to confirm whether the participants consistently performed MI via their single upper extremities as instructed at the beginning. We selected typical EEG channels on the motor cortex corresponding to right-hand (channel C3), left-hand (channel C4), and foot (channel Cz) imagery [54,55]. The mean ERD/ERS values for channel C3 contain significant differences compared with other channels (Cz and C4), as confirmed through the paired *t*-test. All the *P*-values between the channels were <0.05 except for a few participants.

**Figure 8:**
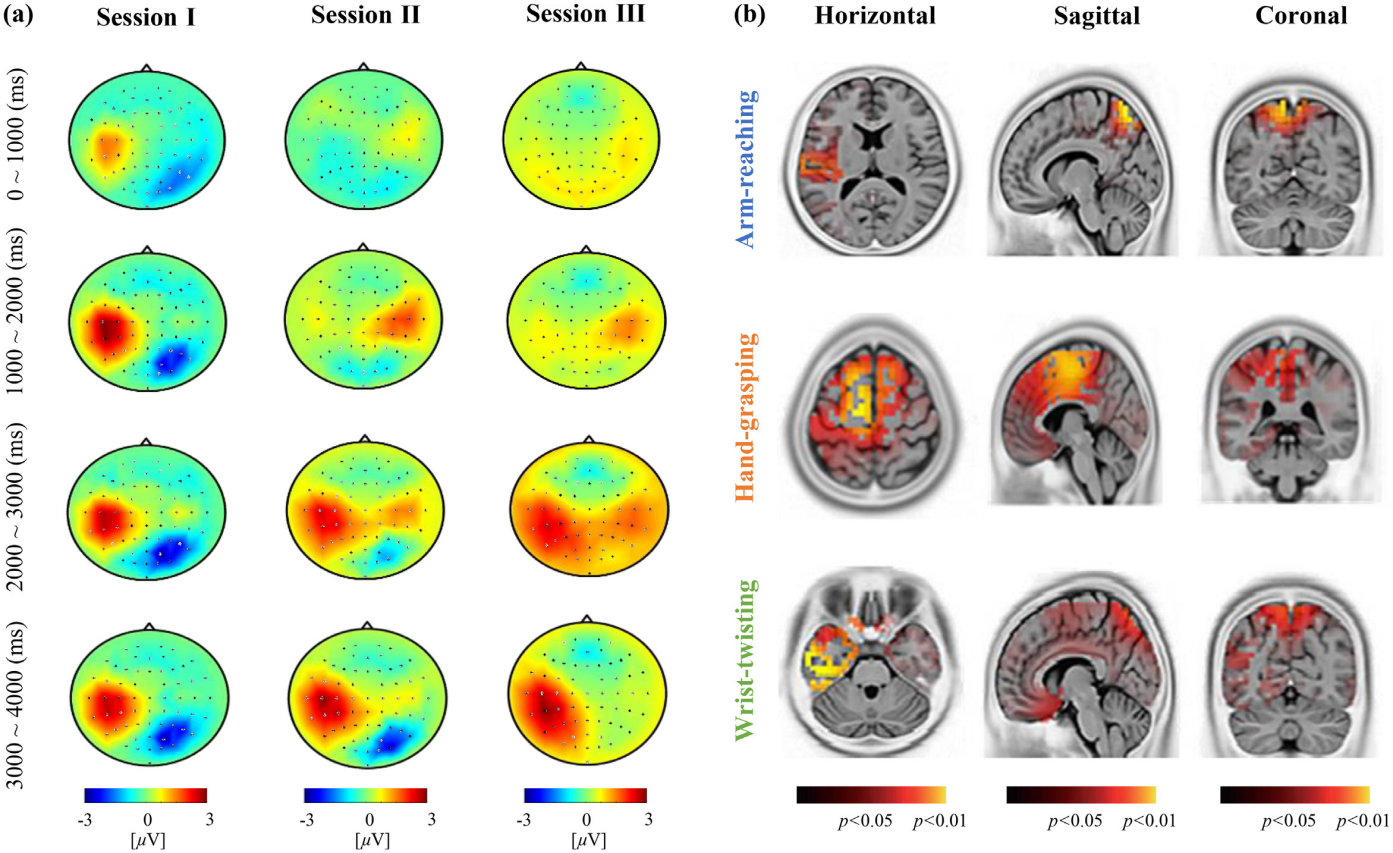
Data validation performed using the spatial information with respect to scalp activation. (a) Scalp topography of the wrist pronation task for a representative participant, No. 2. (b) Source imaging analysis performed via sLORETA using a statistically significant difference in the MI tasks for a representative participant, No. 13 (red: *P* < 0.05, yellow: *P* < 0.01).

Fig. 8a depicts the representative spatial distribution obtained using grand-average signal amplitude responses per time period [56,57]. For participant No. 2, the data for wrist pronation is presented in Fig. 8a. We used all of the EEG channels and adopted signal processing (similar to that in the pre-processing steps), such as band-pass filtering and epoch segmentation, and we also used a baseline period. We applied the moving average of EEG amplitudes with a 200-ms interval. The topographic maps of the mean amplitudes were visualized in 4 temporal intervals for all the recording sessions. Therefore, the left hemisphere of the contralateral sensorimotor region was activated while performing tasks in all the recording sessions. Hence, over time, the mean amplitude in the contralateral sensorimotor region of the left hemisphere significantly increased clearly as the participants performed MI. Moreover, it was clearly observed that the contralateral sensorimotor regions of the most participants were activated by MI in the 3–4 s period. Therefore, we confirmed 0–2 s as the preparation period and that the contralateral sensorimotor region was appropriately activated after the first 2 s in the recording phases.

For a more sophisticated analysis, we used a source imaging technique to identify and compare activated regions in the brain with each other while a participant was performing each task. We used a standardized low-resolution electromagnetic tomography (sLORETA)-based current density estimation technique for inverse modeling the brain points that were activated from the EEG signals. sLORETA is a variant of the weighted minimum norm estimation technique for obtaining an inverse solution [31,58]. We visualized the activated regions of the brain for each task and showed them in terms of the horizontal, sagittal, and coronal planes, as shown in Fig. 8b. The source images were visualized by the significant differences by calculating the *P*-values for the spatial distribution between the baseline period (−0.5 to 0 s) and MI period (0 to 4 s). The yellow colors indicate *P*-values <0.01, and the red colors *P*-values <0.05 [59]. The main differences were observed in the supplementary motor region and premotor cortex; they indicate that the participants satisfactorily performed MI. Because all the participants performed MI related to the movement of the right upper limb, it can be confirmed from Fig. 8b that the left side region of the cortex associated with the MI is activated.

We evaluated the dataset quality by observing BCI classification performance (see Table 2). By using the baseline machine learning method, we confirmed that the accuracies were at least higher than chance-level accuracy for each class. The performances were validated according to tasks including arm-reaching, hand-grasping, and wrist-twisting. We computed the chance-level accuracies with a significant confidence level (α = 5%) [60] and could obtain the chance results per evaluation as 0.17 (arm-reaching), 0.34 (hand-grasping), and 0.51 (wrist-twisting). Table 2 represents the classification accuracies with the standard deviation for each participant and recording session. Because our dataset was recorded over 3 different sessions, it allows for further research related to BCI calibration problems. According to our classification result obtained using the baseline decoding method and conventional approach, some participants showed a significant change in classification results between sessions; however, the other participants showed similar classification accuracies over different sessions.

The decoding of intuitive upper extremity movements from EEG signals is a challenging study. However, if the upper extremity movements can be successfully analyzed, BCI technology could be used for many applications. The BCI may be applied to the operation of robotic instruments, such as a robotic arm and neuro-prosthesis related to upper extremity movements, or to control peripheral devices using commands based on decoding the movement intention. In this study, to enable additional advances in the technology, we provide a multimodal signal dataset when the participants executed and imagined the intuitive movement tasks using a single arm. As mentioned in “Experimental paradigm,” decoding various tasks from the same limb could provide various BCI classes and significantly intuitive communication between users and BCI systems as compared with a typical paradigm. Therefore, a robust decoding model for this dataset could contribute one step toward the advancement of a practical and commercial BCI. Therefore, one must obtain high-quality data. Additionally, more advanced analyses can be attempted because we have constructed a database that includes not only the EOG and EMG data but also the EEG data. For example, the EOG data might be used to remove the noise due to explicit eye movement from the EEG data. Additionally, the EMG data may demonstrate the integrity of the EEG data by showing that no movement-related interference was present in the EMG data in the analysis and MI tasks associated with the EEG. In this work, we recorded the signals' data using 3 modalities, namely, EEG, EOG, and EMG. Additionally, we collected data from 25 participants and divided the experiment into 3 sessions to prepare the dataset. We also provided the spectral representation and time-spatial distribution of a representative participant according to multiple recording sessions. Generally, we confirmed that the data variability among each recording session did not show any significant differences in our dataset (i.e., *P*< 0.05). Furthermore, we confirmed that the classification accuracy per task was slightly higher than chance-level accuracy using the baseline method. Conversely, despite the difficult tasks involved in the experiments, the participants successfully focused on the experiments so that we could obtain high-quality data. In the future, the users of this dataset can contribute to increasing the present classification accuracy using their novel methodology.

Through preliminary data validation, we determined the sufficient quality of our dataset Further studies can be performed to determine the hidden characteristics and features related to the intention of upper-limb movements using only our EEG data, while the EOG data can be used to filter noise for obtaining clear EEG signals. Simultaneously, researchers can attempt to combine EEG and EMG signals using our dataset for developing hybrid BCI systems. In related studies, the hybrid approaches showed remarkable possibility to improve the decoding performance of real-movement and MI-based BCIs [34,61]. Additionally, our dataset can be used for studies that analyze the correlations between EEG and EMG. In related studies, the relevance of EEG and EMG signals can be found through the connectivity analysis of the data acquired over a specific period. For example, a statistical analysis of activated EEG channels conducted during the activation of a particular EMG channel can determine the region of the brain, channel location, and frequency band directly related to the movement of the particular muscle [62].

Inter-session comparisons are also important topics in BCI experiments. Because the BCI systems are recalibrated at the beginning of each recording session, this procedure becomes time-consuming and thus may limit the adoption of BCI systems for long-term daily use [63]. Furthermore, we recorded data over 3 sessions to enable cross-session analysis. For each session, we collected a dataset of uniform quality on the basis of classification results (see Table 2) because we focused on conducting all the experiments under stable conditions. Researchers can analyze the decoding performance using our dataset from the entire session and they can also compare the decoding results of each session with each other. Different approaches are also available, and they include training the decoding model in a particular session and testing the model using data from independent sessions on the basis of the principle of transfer learning in BCI, as done in [64]. Accordingly, creating a session-independent BCI decoding model is critical to establishing a practical BCI system such as a biometric authentication system [65] or brain-controlled augmented reality/virtual reality system [66]. Therefore, our experimental data can be useful for studies to build session-independent decoding models.

## Availability of Supporting Data and Materials

The data supporting this article, including EEG, EMG, and EOG datasets and example codes, are available in the GigaDB repository [67].

## Abbreviations

BCI: brain–computer interface; CSP: common spatial pattern; EEG: electroencephalography; EMG: electromyography; EOG: electro-oculography; ERD/ERS: event-related desynchronization/synchronization; ERSP: event-related spectral perturbation; IC: independent component; ICA: independent component analysis; LCD: liquid-crystal display; MI: motor imagery; RLDA: regularized linear discriminant analysis; sLORETA: standardized low-resolution electromagnetic tomography.

## Ethical Approval

This study was reviewed and approved by the Institutional Review Board at Korea University (1040548-KU-IRB-17-181-A-2).

## Competing Interests

The authors declare that they have no competing interests.

## Funding

This work was partly supported by Institute of Information and Communications Technology Planning and Evaluation (IITP) grants funded by the Korea government (No. 2015-0-00185, Development of Intelligent Pattern Recognition Softwares for Ambulatory Brain-Computer Interface; No. 2017-0-00451, Development of BCI based Brain and Cognitive Computing Technology for Recognizing User's Intentions using Deep Learning; No. 2019-0-00079, Department of Artificial Intelligence, Korea University).

## Authors' Contributions

J.-H.J., J.-H.C., and K.-H.S. designed the experimental protocols and paradigms. J.-H.J., B.-H.K., B.-H.L., D.-Y.L., and D.-H.L. collected the data and checked the physical and mental states of the participants during the experiments. J.-H.J., J.-H.C., and S.-W.L. revised the manuscript. All authors analyzed and validated the collected data technically. Furthermore, all authors prepared the manuscript and approved the public database.

## Supplementary Material

giaa098_GIGA-D-20-00075_Original_Submission

giaa098_GIGA-D-20-00075_Revision_1

giaa098_GIGA-D-20-00075_Revision_2

giaa098_Response_to_Reviewer_Comments_Original_Submission

giaa098_Response_to_Reviewer_Comments_Revision_1

giaa098_Reviewer_1_Report_Original_Submission Georgios Dimitrakopoulos -- 5/6/2020 Reviewed

giaa098_Reviewer_1_Report_Revision_1 Georgios Dimitrakopoulos -- 8/17/2020 Reviewed

giaa098_Reviewer_2_Report_Original_Submission M Mousavi -- 5/16/2020 Reviewed
