# Supplementary material for: Multimodal signal dataset for 11 intuitive movement tasks from single upper extremity during multiple recording sessions
Source: Gigascience. 2020 Oct 7;9(10):giaa098. doi: 10.1093/gigascience/giaa098 (PMC7539536; doi:10.1093/gigascience/giaa098)
Supplement: giaa098_GIGA-D-20-00075_Original_Submission [file giaa098_giga-d-20-00075_original_submission.pdf]

GigaScience

Intuitive brain-computer interface dataset for 11 upper extremity movement tasks during multiple recording sessions

--Manuscript Draft--

**Manuscript Number:**	GIGA-D-20-00075
**Full Title:**	Intuitive brain-computer interface dataset for 11 upper extremity movement tasks during multiple recording sessions
**Article Type:**	Data Note
**Funding Information:**	Institute of Information & Communications Technology Planning & Evaluation (IITP) grant (2017-0-00432) — Dr. Seong-Whan Lee<br>Institute of Information & Communications Technology Planning & Evaluation (IITP) grant (2017-0-00451) — Dr. Seong-Whan Lee<br>Institute of Information & Communications Technology Planning & Evaluation (IITP) grant (2019-0-00079) — Dr. Seong-Whan Lee
**Abstract:**	Background: Non-invasive brain-computer interfaces (BCIs) have been developed for natural bi-directional interaction between users and external robotic systems. However, communication between users and BCI systems through artificial matching is a critical issue. Recently, BCIs have been developed to adopt intuitive decoding which is the key for solving several problems such as a small number of classes or manually matching BCI commands with device control. Unfortunately, advances in this area have been slow owing to the lack of large and uniform datasets. This study provides a large intuitive dataset for 11 different upper extremity movement tasks obtained during multiple recording sessions. The dataset includes 60-channel electroencephalography (EEG), 7-channel electromyography (EMG), and 4-channel electrooculography (EOG) of 25 healthy subjects, from three-day sessions, and for 82,500 trials across all subjects. Findings: We validated our dataset with neuro-physiological analysis. We clearly obtained sensorimotor de-/activation and spatial distribution related to the real movement and motor imagery (MI). Further, we showed that this is a consistent dataset by evaluating the classification performance of each session using a basic machine learning method. Conclusions: This dataset included multiple recording sessions, various classes within the same limbs, and multi-modality signals. This study could be used to i) compare the brain activities of real movement and imagination, ii) improve decoding performance, and iii) analyze the differences in the recording sessions. We believe that the dataset can contribute to advances in the intuitive BCI and real-world scenarios using various upper extremity movement decoding.
**Corresponding Author:**	Seong-Whan Lee<br>Korea University<br>Seoul, KOREA, REPUBLIC OF
**Corresponding Author Secondary Information:**	
**Corresponding Author's Institution:**	Korea University
**Corresponding Author's Secondary Institution:**	
**First Author:**	Ji-Hoon Jeong
**First Author Secondary Information:**	
**Order of Authors:**	Ji-Hoon Jeong
	Jeong-Hyun Cho
	Kyung-Hwan Shim

Powered by Editorial Manager® and ProduXion Manager® from Aries Systems Corporation

	Byoung-Hee Kwon
	Byeong-Hoo Lee
	Do-Yeun Lee
	Dae-Hyeok Lee
	Seong-Whan Lee
**Order of Authors Secondary Information:**	
**Additional Information:**	
**Question**	**Response**
Are you submitting this manuscript to a special series or article collection?	No
**Experimental design and statistics**<br><br>Full details of the experimental design and statistical methods used should be given in the Methods section, as detailed in our Minimum Standards Reporting Checklist. Information essential to interpreting the data presented should be made available in the figure legends.<br><br>Have you included all the information requested in your manuscript?	Yes
**Resources**<br><br>A description of all resources used, including antibodies, cell lines, animals and software tools, with enough information to allow them to be uniquely identified, should be included in the Methods section. Authors are strongly encouraged to cite Research Resource Identifiers (RRIDs) for antibodies, model organisms and tools, where possible.<br><br>Have you included the information requested as detailed in our Minimum Standards Reporting Checklist?	Yes
**Availability of data and materials**<br><br>All datasets and code on which the conclusions of the paper rely must be	Yes

Powered by Editorial Manager® and ProduXion Manager® from Aries Systems Corporation

either included in your submission or deposited in publicly available repositories (where available and ethically appropriate), referencing such data using a unique identifier in the references and in the "Availability of Data and Materials" section of your manuscript.<br><br>Have you have met the above requirement as detailed in our Minimum Standards Reporting Checklist?	

Powered by Editorial Manager® and ProduXion Manager® from Aries Systems Corporation

Manuscript Click here to download Manuscript Manuscript_jhjeong.pdf

OXFORD (GIGA)^n SCIENCE

GigaScience, 2020, 1–10

doi: xx.xxxx/xxxx
Manuscript in Preparation
Data Note

DATA NOTE

Intuitive brain-computer interface dataset for 11 upper extremity movement tasks during multiple recording sessions

Ji-Hoon Jeong[1], Jeong-Hyun Cho[1], Kyung-Hwan Shim[1], Byoung-Hee Kwon[1], Byeong-Hoo Lee[1], Do-Yeun Lee[1], Dae-Hyeok Lee[1] and Seong-Whan Lee[2,*]

[1]Department of Brain and Cognitive Engineering, Korea University, 145 Anam-ro, Seongbuk-gu, Seoul, 02841, Republic of Korea and [2]Department of Artificial Intelligence, Korea University, 145 Anam-ro, Seongbuk-gu, Seoul, 02841, Republic of Korea.

[*]**Correspondence address**. Seong-Whan Lee, Department of Artificial Intelligence, Korea University, 145 Anam-ro, Seongbuk-gu, Seoul, 02841, Republic of Korea. Tel: +82-2-3290-3197; Fax: +82-2-3290-3583; E-mail: sw.lee@korea.ac.kr

Abstract

Background: Non-invasive brain-computer interfaces (BCIs) have been developed for natural bi-directional interaction between users and external robotic systems. However, communication between users and BCI systems through artificial matching is a critical issue. Recently, BCIs have been developed to adopt intuitive decoding which is the key for solving several problems such as a small number of classes or manually matching BCI commands with device control. Unfortunately, advances in this area have been slow owing to the lack of large and uniform datasets. This study provides a large intuitive dataset for 11 different upper extremity movement tasks obtained during multiple recording sessions. The dataset includes 60-channel electroencephalography (EEG), 7-channel electromyography (EMG), and 4-channel electrooculography (EOG) of 25 healthy subjects, from three-day sessions, and for 82,500 trials across all subjects.
Findings: We validated our dataset with neuro-physiological analysis. We clearly obtained sensorimotor de-/activation and spatial distribution related to the real movement and motor imagery (MI). Further, we showed that this is a consistent dataset by evaluating the classification performance of each session using a basic machine learning method.
Conclusions: This dataset included multiple recording sessions, various classes within the same limbs, and multi-modality signals. This study could be used to *i*) compare the brain activities of real movement and imagination, *ii*) improve decoding performance, and *iii*) analyze the differences in the recording sessions. We believe that the dataset can contribute to advances in the intuitive BCI and real-world scenarios using various upper extremity movement decoding.

Key words: brain-computer interface (BCI); physiological signals; intuitive upper extremity movement; multiple sessions

Data Description

Background and purpose

Brain-computer interface (BCI) technology allows users to communicate with external devices such as a speller [1], wheelchair [2], robotic arm [3–5] and robotic exoskeleton [6, 7]. Noninvasive BCI commonly adopt electroencephalography (EEG) signals for decoding user intentions [8–10] because the EEG-based BCI system has low risk, incurs lower cost, and is easier to use than other non-invasive BCI paradigms (e.g., functional near-infrared spectroscopy (fNIRS) [11] or magne-

Compiled on: March 13, 2020.
Draft manuscript prepared by the author.

toencephalography (MEG). EEG-based BCIs have been developed using various paradigms such as motor imagery [12–14], steady-state visual evoked potential (SSVEP) [15, 16], event-related potential (ERP) [17], and movement-related cortical potential (MRCP) [18, 19]. Over the past decades, EEG datasets of these general paradigms have been published through competitions, cooperation projects, and an open-access article [20–23]. Some research groups have developed advanced machine learning algorithms and deep learning architectures for improving BCI performance using these datasets.

Recent advances in BCI systems have focused on intuitive decoding without artificial interaction between user thought and device feedback for the real-world environment [19, 24, 25]. For example, to control a neuro-prosthetic arm using typical paradigms, we temporally matched BCI commands with robotic arm motions (e.g., motor imagery for both hands = grasping motion of robotic hand). However, this temporal matching has several limitations, such as constrained classes for communicating with devices and inflexible user training due to unintuitive commands in real-world scenarios [7, 12].

In this work, we collected data on the intuitive upper extremity movements from 25 subjects. To collect high-quality signal data, the experiment was conducted on healthy subjects who maintained good physical conditions by, for instance, limiting alcohol intake and getting sufficient sleep. We focused on various motions of the upper extremities because they have the most extensibility and availability in the body. Therefore, we selected the upper extremities for decoding intuitive movements and collected data based on multi-modality signals related to the movements. The subjects were asked to perform 11 different movement tasks: arm-reaching for six multiple directions, hand-grasping for three objects, and wrist-twisting with two different motions. The corresponding 11 classes were designed for each segmented motion related to the arm, hand, and wrist, rather than simply for continuous limb movements. Therefore, users of our data can either conduct respective analyses for individual classes or try decoding complex upper extremity movements by combining data from different classes. For researchers focused on more advanced and analytical approaches using multi-modality signals, the dataset comprises not only EEG data but also electromyography (EMG) and electrooculography (EOG) data. These data were collected synchronously in the same experimental environment, while ensuring that there was no unintentional interference between them. Data from a 60-channel EEG, 7-channel EMG, and 4-channel EOG were simultaneously used for data recording during the experiment. EEG sensors were placed according to international specifications to collect signals from all areas of the scalp, and EMG sensors were attached to carefully selected locations on the right arm to reflect the most relevant muscle activity information associated with the corresponding upper limb movement. We also recorded the EOG signal using four channels independent of the EEG channels to capture detailed eye movement, which were mainly used for artifact removal. The subjects performed real movements of their upper extremities and motor imagery of the 11 motions. In addition, each subject participated in three recording sessions at one-week intervals with the same experimental protocols. To acquire a large-amount of high-quality data, we considered the subject's physical and mental condition as a priority during the experiment. This intuitive BCI dataset became a large dataset for the BCI experiment because it included 82,500 trials across all subjects (i.e., 3,300 trials were collected per subject).

To the best of our knowledge, the present dataset descriptor is the first large public dataset for intuitive BCI paradigms that includes EEG, EMG, and EOG data. We believe that this work will contribute for the reliable neuro-rehabilitation of motor-disabled patients and a high-level BCI system for healthy users. Many related studies on users' motor intentions have been performed; however, unsatisfactory decoding performance was a significant problem, predominantly when testing in the background of MI and in a session independent situation [26, 27]. To overcome the difficulties of decoding EEG signals, some studies proposed the idea of combining them with EMG signals [28] and adopting a deep learning approach, which has the characteristics of a large dataset for model training [29, 30]. This work can contribute to the development of an intuitive BCI system based on various approaches such as deep learning and multi-modality by providing a large dataset.

Experimental design

Subjects

Twenty-five subjects (S1-S25, aged 24-32 years, 15 males and 10 females) who were naïve BCI users participated in the experiments. The subjects were all healthy individuals with no known neurophysiological anomalies or musculoskeletal disorders. Before the experiment, the volunteers were informed about the experimental protocols, paradigms, and purpose. After ensuring that they had understood, all of them provided their given written consent according to the Declaration of Helsinki. The subjects signed a form providing the anonymous release of their data to the public. We checked the subjects' physical and mental states for comparing the influence of BCI performance according to individual state. In addition, each subject was required to be in normal health, get enough sleep (approximately 8 hours), and avoid alcohol, caffeinated drinks, and strenuous exercise before the experiments. All experimental protocols and environments were reviewed and approved by the Institutional Review Board (IRB) at Korea University (1040548-KU-IRB-17-181-A-2).

Environment

During the experiments, the subjects were seated comfortably in a chair with armrests in front of an LCD monitor approximately 60 ($\pm$5)cm away. An EEG cap (Fig. 1) with 60 channels (ActiCap, BrainProduct GmbH, Germany) was placed on their heads. Surface EMG and EOG electrodes were attached to pre-assigned locations on the subjects' right arm and around the eyes, respectively. The subjects were then asked to perform the movements with relaxed muscles and minimum eye and body movements for the duration of data recording.

The duration of the experiment was approximately 6~7 hours a day. Our experiment comprised multiple recording sessions (three days) to consider inter-session and inter-subject variability. It required a relatively long recording time compared with typical BCI experiments. To maintain the user's physical and mental condition and high signal quality, the subjects took sufficient breaks between each task. During breaks, we checked the impedances of the EEG, EMG, and EOG electrodes and injected electrolyte gel to keep the values below 15 kΩ.

Experimental paradigm

The experiment was designed to quantitatively acquire the 11 different upper extremity movements for real movement and motor imagery tasks. The experimental tasks comprised three main upper-extremity motions: arm-reaching, hand-grasping, and wrist-twisting. When the experiment started, visual instructions were provided on the monitor with a black plus sign and a gray background. The subjects stared at the visual instructions for 4 seconds while resting. After the resting phase, a visual cue was displayed on the monitor with a text sign for 3 seconds, and the subjects prepared to perform the real movement or motor imagery according to the visual

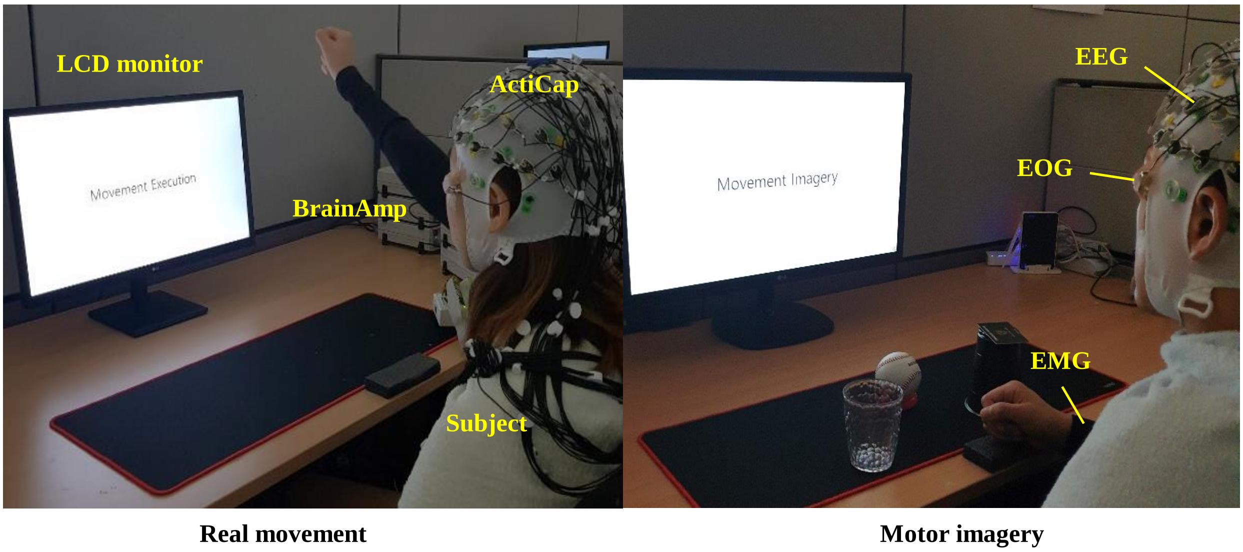

Figure 1. Experimental environments for acquiring intuitive BCI dataset. Subjects were asked to perform real movement tasks (e.g., arm-reaching) and motor imagery tasks (e.g., hand-grasping).

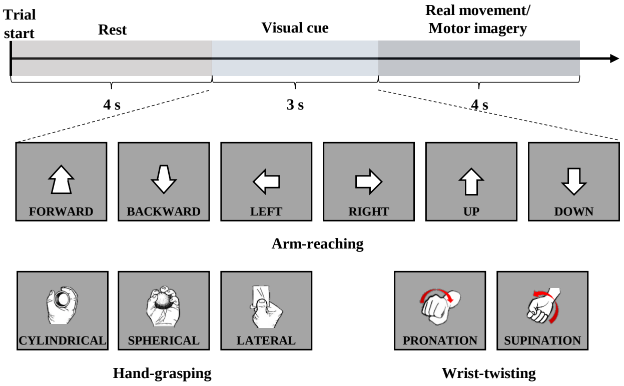

Figure 2. Experimental paradigm in a single-trial and the representation of visual cues according to each task.

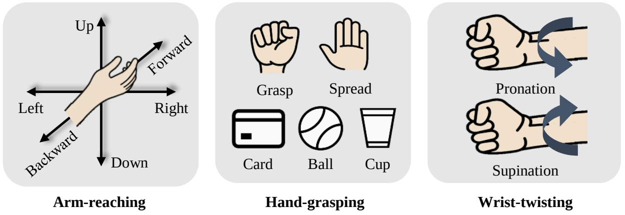

Figure 3. Experiment tasks of 11 intuitive upper extremity movements related to arm-reaching, hand-grasping, and wrist-twisting.

cue (Fig. 2). When the visual cue was changed to the text sign, the subjects performed the task for 4 seconds. During the real movement tasks, the subjects were asked to focus on the sensations involved in each motion to remember them for the motor imagery tasks.

Arm-reaching in six directions: We asked the subjects to perform multi-direction arm-reaching tasks from the center of their bodies in the outward direction. The subjects performed it in six different directions 3D space; forward, backward, left, right, up, and down as depicted in Fig. 3. In the real movement tasks, the subjects reached their arms in one of the directions. The arm-reaching paradigm required 50 trials in each direction so that data could be collected for a total of 300 trials. In the motor imagery tasks, the subjects only imagined performing an arm-reaching task. The number of trials was the same as in the real movement paradigm.

Hand-grasping of three objects: the experiment asked the subjects to grasp three objects of daily use with the corresponding grasp motions. The subjects performed the three designated grasp motions by holding the actual objects; card, ball, and cup corresponding to cylindrical grasp, spherical grasp, and lateral grasp, respectively (Fig. 3). In the real movement tasks, we asked the subjects to use their right hand to grasp one of the objects that were randomly selected and hold it with the specific grasping motion. We acquired data on 50 trials for each grasp; hence, we collected 150 trials per subject. In the motor imagery tasks, the subjects only one of the three grasping motions per trial, randomly. The number of trials was the same as the that in the real movement paradigm.

Wrist-twisting with two different motions: for the wrist-twisting tasks, the subjects rotated their wrist to the left (pronation) and right (supination) as depicted on Fig. 3. During real movement, the subjects kept their right hand in a neutral position with the elbow placed comfortably on the desk. Wrist pronation and supination are complex actions used to decode user intention from brain signals. In addition, these movements are intuitive motions for neuro-rehabilitation and prosthetic control [31]. We collected data from 50 trials per motion (100 trials in total) a day, while the visual cues were randomly indicated.

Additionally, the subjects were asked to participate in three recording sessions at one-week intervals. The experimental environment and protocols were the same for all three sessions. As a result, we collected data from 3,300 trials in all classes per subject (1,800 trials for arm-reaching, 900 trials for hand-grasping, and 600 trials for wrist-twisting), including real movements and motor imagery paradigms.

Data Records

In this study, we simultaneously collected three different kinds of physiological signals, EEG, EMG, and EOG for 11 different up-

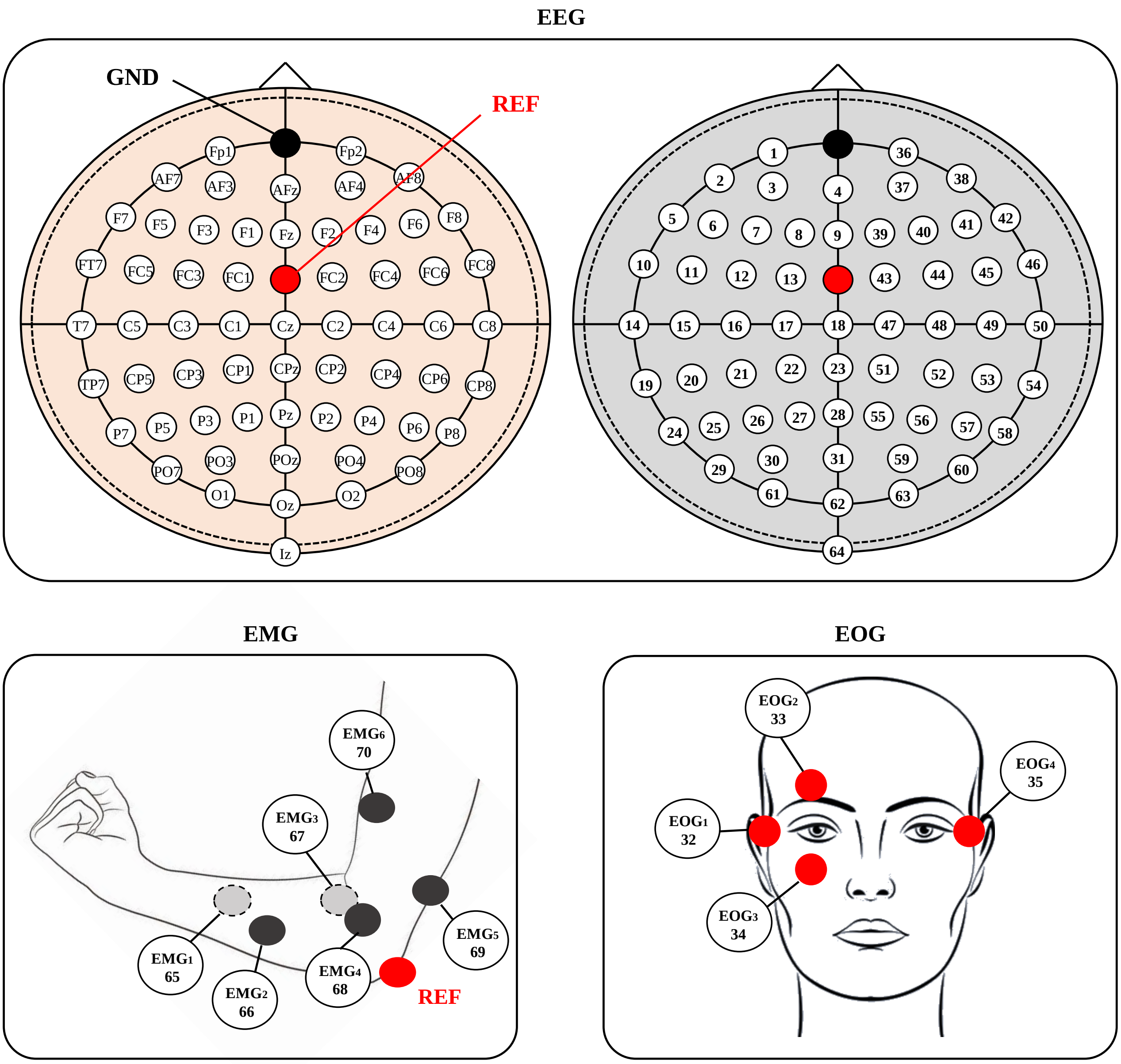

Figure 4. Data configuration for 60-EEG channels, 7-EMG channels, and 4-EOG channels.

per extremity movements (Fig. 3). The signals were acquired using the same digital amplifier and the same types of electrodes during the experiment. Therefore, the raw signals were stored together in one data file according to each subject. To obtain high-quality signals, the impedances of all channels were maintained below 15 kΩ. After applying the conductive gel, we validated the accuracy of the EEG and EOG signals by asking subjects to blink and close their eyes. The eye-blinking task was used to identify strong spikes in frontal EEG channels (Fp1 and Fp2) and 4-channel EOG. The eye-closing task was used to confirm the alpha oscillations in the occipital channels (e.g., O1, O2, and Oz). We also asked the subjects to perform a simple hand-grasping motion to confirm strong spikes in the EMG signals.

EEG signals

The EEG data were recorded in conjunction with an EEG signal amplifier (BrainAmp, BrainProduct GmbH, Germany), sampled at 2,500 Hz, and applied a 60 Hz with a notch filter. The raw data were recorded using BrainVision (BrainProduct GmbH, Germany) with MATLAB 2019a (The MathWorks Inc., USA). 60 EEG electrodes were selected by following a 10-20 international configuration (Fp1-2, AF5-6, AF7-8, AFz, F1-8, Fz, FT7-8, FC1-6, T7-8, C1-6, Cz, TP7-8, CP1-6, CPz, P1-8, Pz, PO3-4, PO7-8, POz, O1-2, Oz, and Iz). Ground and reference channels were placed on the Fpz and FCz (Fig. 4). The impedance of all electrodes between the sensors and the skin of the scalp were maintained below 15 kΩ. During the break time, conductive gel was injected using a syringe with a blunt injection needle.

EMG signals

EMG signals were recorded using 7 Ag/AgCl electrodes from the digital amplifier, which is the same equipment used to record EEG signals. We simultaneously acquired EMG and EEG signals [28]. The signals were captured at a sampling rate of 2,500 Hz with a 60 Hz notch filter. The EMG data were recorded from six related muscles for right arm movement; extensor carpi ulnaris, extensor digitorum, flexor carpi radialis, flexor carpi ulnaris, biceps brachii, and triceps brachii (Fig. 4) [32]. The last electrode was placed on the elbow of the right arm which is a non-muscle movement area, as a reference signal. The purpose of recording EMG signals was to detect muscle activities

Table 1. Data description for *Raw data* folder and *Converted data* folder

Raw data		Converted data (e.g., session1_sub1_multigrasp_MI.mat)		
Name	Description	Name		Description
session1_sub1_(*Task*)_(realMove/MI).eeg	Raw signals (session I)		.x	Pre-processed data
session1_sub1_(*Task*)_(realMove/MI).vhdr	Data header information (session I)	.dat	.fs	Sampling frequency
session1_sub1_(*Task*)_(realMove/MI).vhdr	Marker information for (session I)		.file	File name
			.clab	Channel information
			.x	X coordinates for channel position
session2_sub1_(*Task*)_(realMove/MI).eeg	Raw signals (session II)	.mnt	.y	Y coordinates for channel position
session2_sub1_(*Task*)_(realMove/MI).vhdr	Data header information (session II)		.pos_3d	3D coordinates for channel position
session2_sub1_(*Task*)_(realMove/MI).vhdr	Marker information for (session II)		.clab	Channel information
			.pos	Trigger marking time
			.toe	Trigger number
session3_sub1_(*Task*)_(realMove/MI).eeg	Raw signals (session III)	.mrk	.fs	Sampling frequency
session3_sub1_(*Task*)_(realMove/MI).vhdr	Data header information (session III)		.y	Class lebels
session3_sub1_(*Task*)_(realMove/MI).vmrk	Marker information for (session III)		.className	Class name
			.mics	Experiment start and end information

when the subjects performed the designated tasks. The signals could prove that the subjects performed motor imagery without muscle movement. At the same time, the placement of electrodes was selected to record sufficient signals from the various arm and hand movements (i.e., six reaching, three grasping, and two twisting actions) during the experiment.

EOG signals

4-channel EOG signals were recorded with the same protocol. FT9, FT10, TP9, and TP10 electrodes were subsequently moved to around the eyes to function as EOG channels to eliminate artifacts from ocular activities. One of these channels was moved to around the left eye and the others to the right eye (Fig. 4). The electrodes EOG_1 and EOG_2 were used to record horizontal eye movements, while the electrodes EOG_3 and EOG_4 were used to measure vertical movements [33]. Medical tape was used to hold the sensors around the eyes and maintain the impedances of all electrodes below 15kΩ.

Data format and structure

Readers can access our codes and datasets through a GigaDB repository. The '*Read me*'.pdf file, which contains the introductions for overview of data description and code execution, is included in the repository. Several useful scripts, named *Data_analysis*.m and *Visualization*.m files, are in the 'SampleCode' folder. We recommend the BBCI (http://www.bci.de) toolbox [34] in the 'Reference toolbox' folder and the 'Signal Processing' toolbox in MATLAB software, for data processing using our custom code. Please contact the authors directly for more information on the code script.

Every dataset (raw signals, converted data, and scripts) is also publicly available via the GigaDB repository. The raw signals and converted data are contained in the corresponding folders (i.e., 'Raw data' and 'Converted data', respectively). Table 1 represents the data description for 'Raw data' folder and 'Converted data' folder briefly. The indicated *Task* includes the names of reaching, multigrasp, and twist. We provide the following: 'Raw data' and 'Converted data' folders comprise *.eeg*, *.vmrk*, *.vhdr*, and *.mat* files according to each subject. In the *.eeg* file, the raw EEG, EOG, and EMG signals are included because of the simultaneous acquisition of data with the same amplifier. *.vmrk* provides the marked trigger information (e.g., trigger number, marked time, and file name) and *.vhdr* includes the number of channels, sampling rate, channel position, and electrode impedances. *.mat* shows that the pre-processed data contain pre-processed EEG, EOG, and EMG data, channels, class information, scalp montage, and sample frequency. Additionally, to access the data more easily for the users, we provided the converted data after basis pre-processing step on the 'Converted data' folder. The converted data (*.mat*) provided the essential information of EEG, EMG, and EOG signals depending on each channel.

Data_Analysis.m provides the basic script for data processing, which includes data load, signal pre-processing, artifact rejection, feature extraction, classification, and performance evaluation. All users can download and unzip the '*SampleData*'.zip file in the 'SampleData' folder before executing each code.

Visualization.m enables the visualization of raw signals, scalp distribution, and event-related spectral perturbation (ERSP) using EEGLAB [35]. The raw signals were visualized as time by channels for all channels. The visualization of the scalp plot can be used to select a specific channel and time epoch for a selected subject. The ERSP plot requires the installation of the EEGLAB toolbox. After setting up the EEGLAB toolbox, users can load the *.vhdr* file to EEGLAB and visualize the ERSP pattern that follows the description.

Data Validation

Methods

The technical signal validation was conducted using a BBCI toolbox [34] through the MATLAB 2019a environment. The initial settings of the recording program for converting an analog signal into a digital signal were slightly different for each signal owing to the scale of the signal amplitude. For each EEG, EMG, and EOG signals, the triggers were marked to indicate the experimental state.

Initially, for data pre-processing, we used a zero-phase fourth-order Butterworth filter for band-pass signal filtering. The data were filtered between 8 and 30 Hz (mu-band and beta-band) which is widely known as the spectral range for somatosensory rhythm (SMR) observation. For artifact rejection, apparent eye-blinking contamination in the EEG signal was removed by using an independent component analysis (ICA) method [36]. To obtain corrected EEG data, we removed the

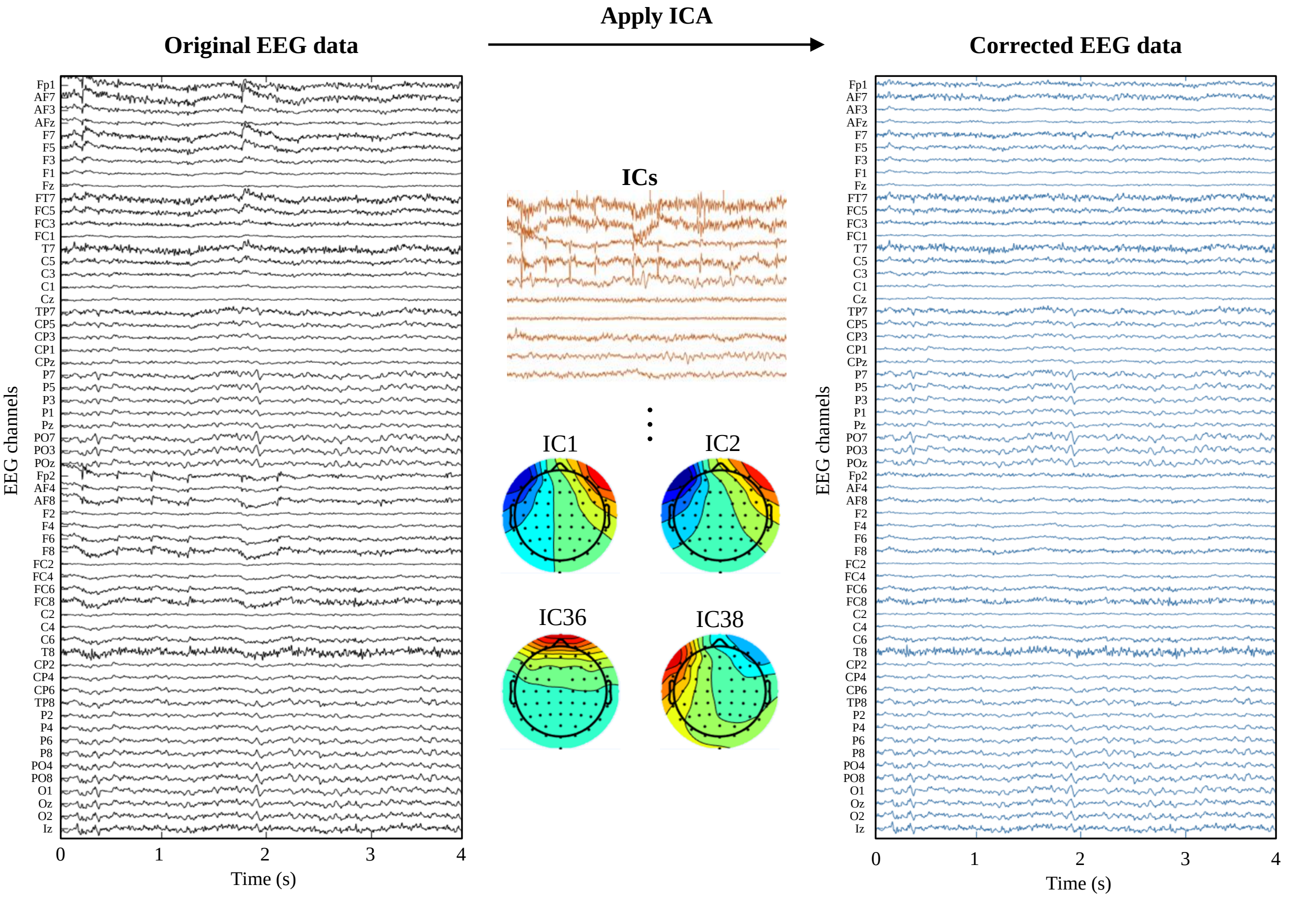

Figure 5. Independent component analysis (ICA) based on EOG data was applied to eliminate noise, caused by eye movement from the original data. The right side shows the corrected EEG data where noise has been eliminated by the application of ICA to the original EEG data.

contamination factors using the ICA algorithm, which is commonly used to decompose the brain signals into statistically independent components (ICs). The EEG data were transformed by the ICA mixing matrix. The contaminated ICs with patterns similar to the EOG channels (i.e., horizontal and vertical eye movements) were removed. The remaining ICs were projected back into the scalp channel space to be reconstructed as the corrected EEG data (Fig. 5).

Then, we checked the EMG activation to verify whether the upper extremity movement or not according to the tasks. The EMG signals could show how well the subject performed the experimental protocol for each task. For example, if the subject were asked to perform the motor imagery task, the EMG activation should not show the peak shape owing to the static state at that time as depicted in Fig. 6. Meanwhile, in the real movement tasks, the EMG signals could show the activation while the subject was performing an upper extremity movement task.

In this work, the EMG signals were band-pass filtered from 50–250 Hz with a Butterworth fifth-order zero-phase shift bandpass filter [28]. We segmented a time interval of -0.5 s~-4 s for EMG data analysis. We selected an interval of -0.5 s~0 s for as the baseline time period. Then, the data were rectified using the absolute values, and we calculated the moving average of EMG amplitudes with a 100 ms interval.

For performance evaluation, we adopted a common spatial pattern (CSP) algorithm as a feature extraction method and a regularized linear discriminant analysis (RLDA) method as the classification method. The CSP+RLDA method is commonly used for decoding motor imagery and execution in the BCI field [24, 37]. The CSP algorithm has been proved to be robust at

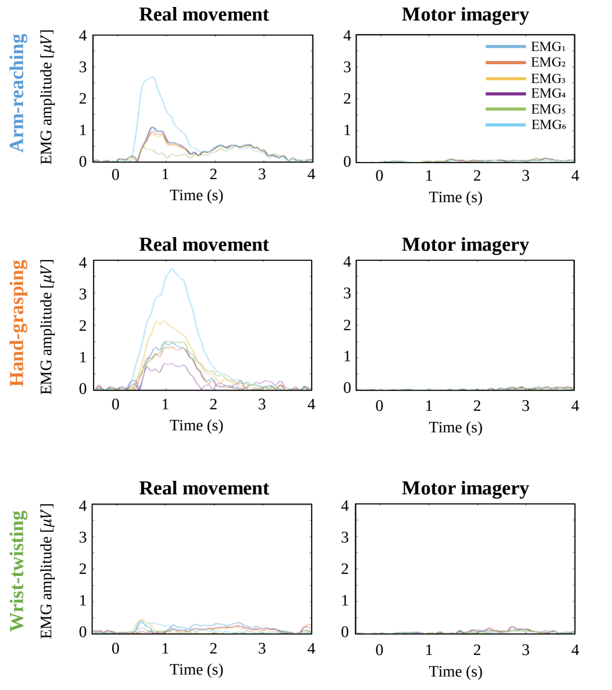

Figure 6. The example representation of EMG activation according to each 7-channel EMG. From the top, the plot represents activities of EMG signals during the representative subject S4 performs arm-reaching, hand-grasping, and wrist-twisting tasks, respectively.

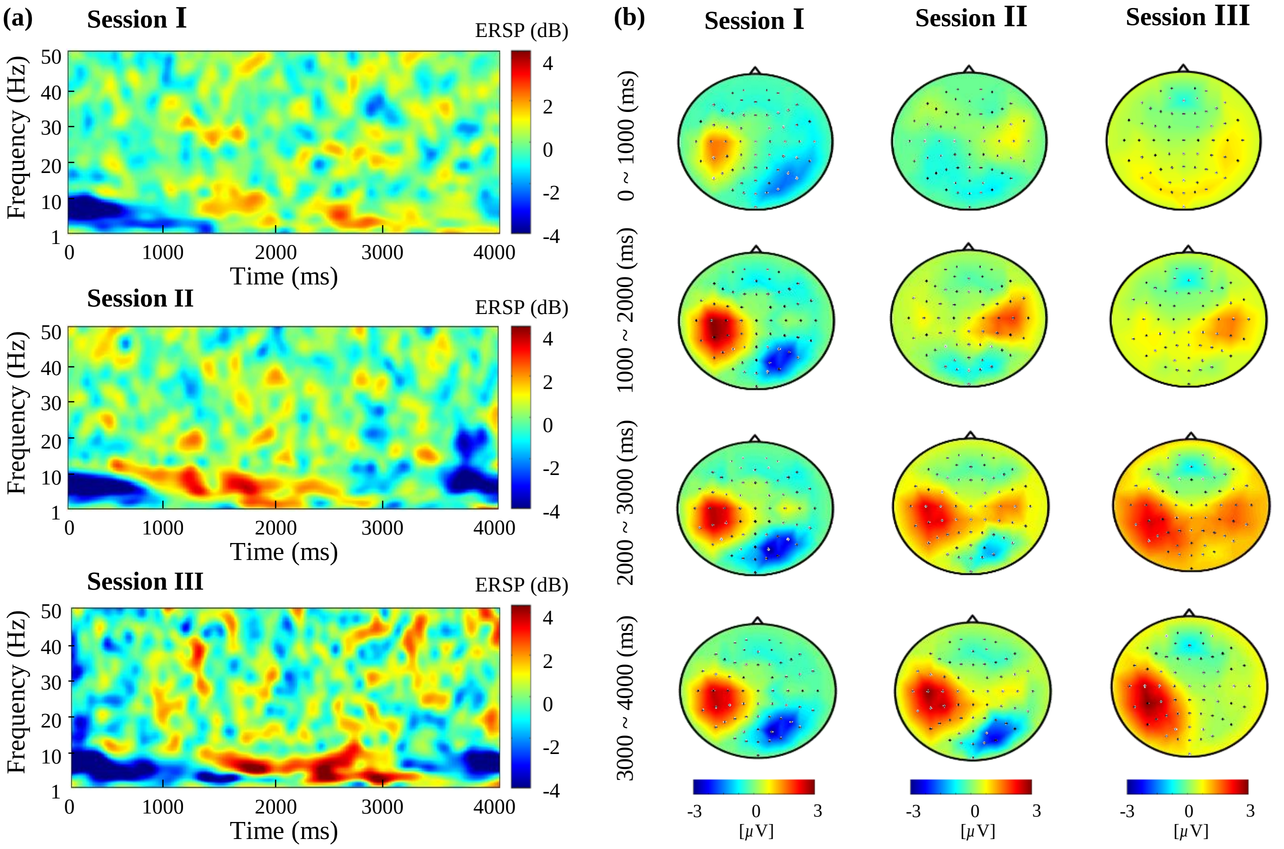

Figure 7. EEG data validation using spectral and spatial representation. (a) ERD/ERS representation of channel C3 during multiple recording sessions. (b) Scalp topography of wrist pronation task for representative subject S2.

spatial feature extraction for decoding movement-related tasks and MI [37]. In this work, we calculated a transformation matrix from CSP consisting of the logarithmic variances of the first three and last three columns were used as a feature. For multi-class classification, we trained the RLDA classifier by adding a regularization term to a covariance matrix using the optimal shrinkage parameter [38]. We applied 10-fold cross-validation for fair performance measurement.

Results and Discussion

We verified the data through the classification results based on EEG, the quality of the EMG signals, and spectral and spatial presentation of EEG. First, we verified the data based on the quality of EMG signals, as shown in Fig. 6. Through the EMG signals recorded while the subjects performed each arm-reaching, hand-grasping, and wrist-twisting experiment, we could confirm the quality of the data obtained. Dynamic EMG signals were observed in the real movement sessions; little changes were observed in the EMG signals close to the rest state in the motor imagery sessions. The EMG signals, in particular, prove that we collected the data properly, given that they were not activated across the entire duration of the actual motion session, and appeared strongly for the actual movements of the subjects right after approximately 0.5 seconds from the onset. In addition, the non-activated EMG signals during the motor image clearly indicate that the movement artifacts of the corresponding EEG signal were minimized.

We also analyzed the EEG signals in the spectral and spatial domain to confirm the quality of the data. Fig. 7 shows examples of spectral energy and spatial activation distribution in the EEG data for a representative subject during multiple recording sessions. Fig. 7(a) is an ERSP plot that illustrates spectral variability according to the time epoch in a certain channel (C3). The ERSP showed event-related de-synchronization (ERD/ERS) patterns, which reflect sensorimotor activation and

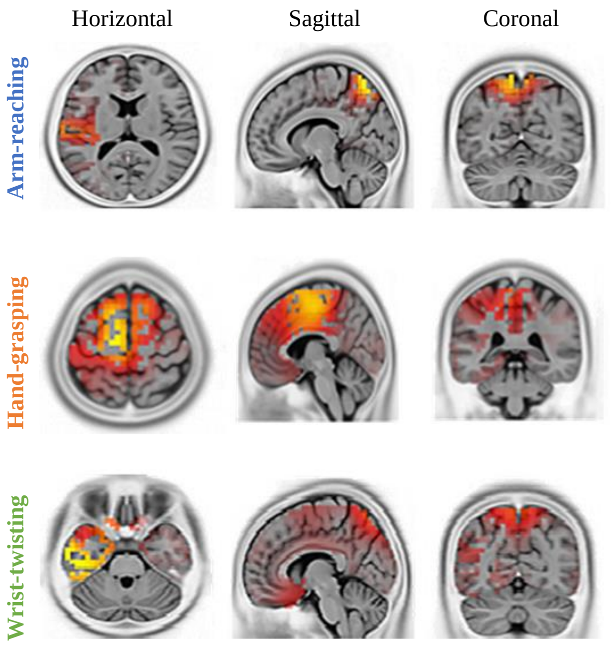

Figure 8. Data validation based on source imaging using sLORETA. Brain images were visualized using EEG data recorded in the motor imagery session.

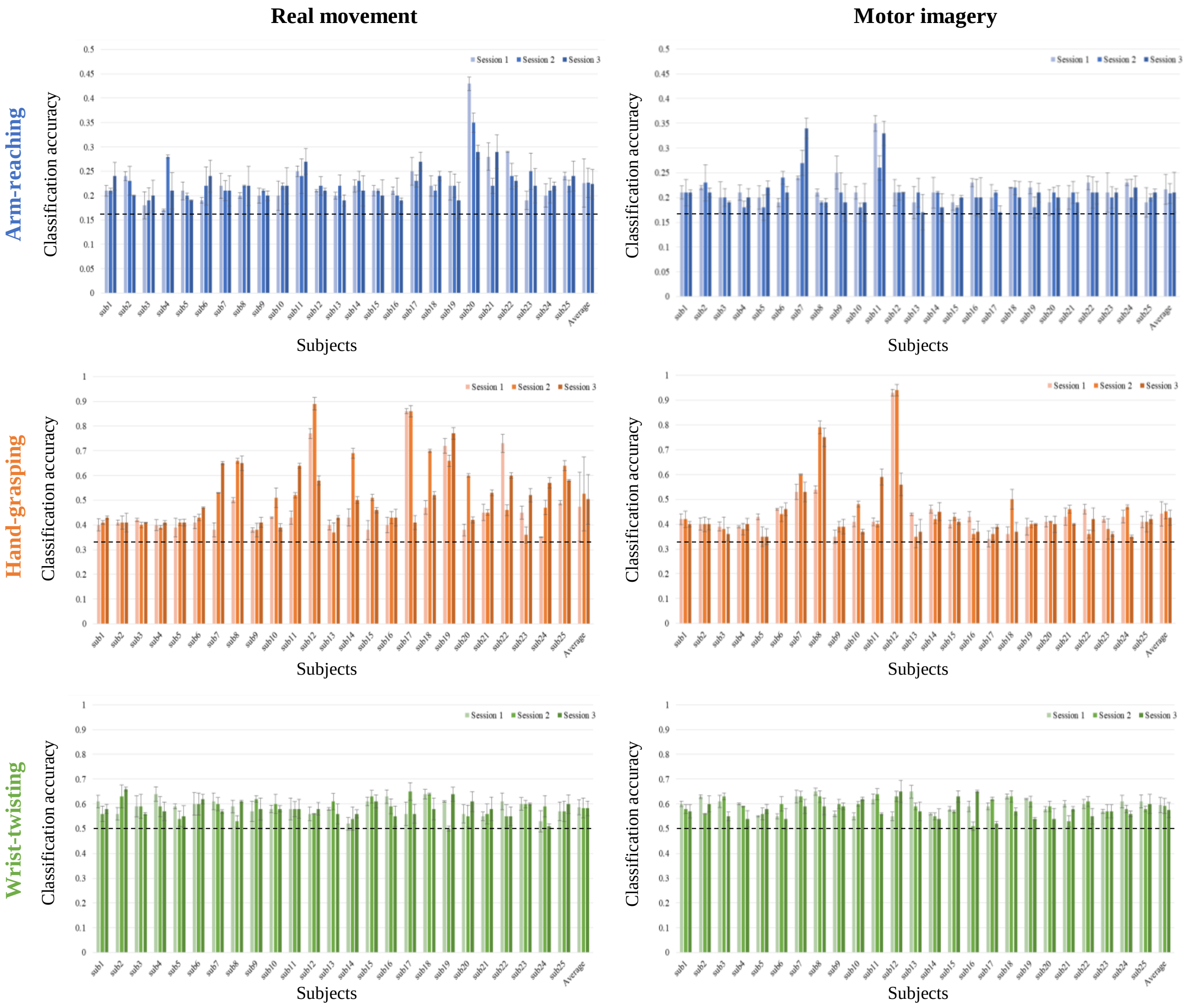

Figure 9. Classification accuracy for each task across all subjects during multiple recording session. EEG data obtained from each upper limb movement (6-, 3-, and 2-class, respectively) was classified and compared in real movement and motor imagery tasks.

deactivation. The ERD patterns can be seen during motor preparation, execution, and imagery as the correlations in an activated cortical area. ERS can be observed after the imagery or execution of movement over the same area [39, 40]. Fig. 7(a) clearly shows the same cortical activation such as ERD/ERS patterns, on the mu-band across all recording sessions. Fig. 7(b) shows the scalp topographies for spatial distribution for each 1-second time epoch. In our dataset, the subjects performed wrist pronation using their right hand. Therefore, the left-hemisphere of the contralateral sensorimotor area was activated while performing tasks in all recording sessions.

For more sophisticated analysis, we used source imaging technique to identify and compare activated regions in the brain when the subject was performing each task. We used a standardized low-resolution electromagnetic tomography (sLORETA)-based current density estimation technique for inverse modeling the points of the brain that were active from the EEG signals. sLORETA is a variant of the weighted minimum norm estimation technique for an inverse solution [31, 41]. We visualized the activated regions of the brain in relation to each task and showed them in terms of the horizontal, sagittal, and coronal planes, as shown in Fig. 8. In the figure, the spatial distribution of each task was projected onto a rendered brain to visualize the spatial distribution on the cortex. The color on the brain image indicates the contribution of that dipole location for separating a given task from the other two, with the brightest colors, yellow and orange indicating the strongest representation. Note that, in the source imaging result, the observed regions are those that are strongly activated only on the motor cortex when the subject performs motor imagery. It is encouraging that the strongly activated regions are mainly concentrated on the left side, because the imagery is associated with the right arm.

We evaluated the quality of the dataset through BCI classification performance (Fig. 9). We confirmed that the performances were at least higher than chance-level accuracy for each class by using the basic machine learning method. The performances were validated according to the following task: arm-reaching (6-class), hand-grasping (3-class), and wrist-twisting (2-class). Therefore, the chancel-level accuracies of each evaluation were computed as 0.16 (6-class), 0.33 (3-class), and 0.50 (2-class), respectively. In the figure, each bar represents classification accuracy, and the three bars of different shades for each subject represent the results for each of the three sessions. Because our data were recorded over three sessions, it allows further research related to session dependency or independency. According to our classification result obtained using a basic decoding method and conventional ap-

proach, some subjects showed a significant change in classification results with the session changing; however, other subjects showed similar classification accuracies over different session. Further, there were some high-performance subjects in the arm-reaching and hand-grasping tasks, but most subjects showed similar levels of classification accuracy in the wrist-twisting task.

Decoding intuitive upper extremity movements from EEG signals is challenging. However, if the upper extremity movements can be successfully analyzed, BCI technology can be applied to many applications based on it. The BCI may be applied to the operation of robotic instruments, such as a robotic arm or neuro-prosthesis related to upper extremity movements, or to control peripheral devices based on these commands by decoding movement intention. For this, it is essential to obtain high quality data. In addition, more advanced analyses can be attempted because we have constructed a database including EOG and EMG as well as EEG data. For example, the EOG data might apply noise removal related to explicit eye movement for EEG data, and EMG data may demonstrate the integrity of EEG data by showing that there was no interference in the EMG data in the analysis and motor imagery tasks of the EEG related to movement. In this work, we recorded signals' data through three modalities, EEG, EOG, and EMG, and also collected data from 25 subjects and divided the experiment into three sessions to prepare a dataset. We also analyzed whether the data were inter-session dependent or independent. Creating a BCI analysis model that is independent of the session is critical for establishing a practical BCI system. Therefore, our experimental data can be useful for studies that build session-independent decoding models like this.

Through preliminary data validation, we determined the sufficient quality of our dataset. Studies can be done to find the hidden characteristics and features related to the intention of the upper limb movements using only our EEG data, while EOG data can be used for noise removal to filter for obtaining clear EEG signals. At the same time, researchers can attempt to combine EEG and EMG signals using our dataset for developing hybrid BCI systems. In related studies, the hybrid approaches showed remarkable possibility to improve the decoding performance for the real movement and motor imagery BCIs. In addition, our dataset can be used for studies that analyze the correlations between EEG and EMG. In related studies, the relevance of EEG and EMG signals can be found through connectivity analysis of the data acquired over a specific period. For example, a statistical analysis of the activated EEG channels conducted at a time similar to the activation of a particular EMG channel can determine the region of the brain, the channel location, and the frequency band directly related to the movement of the particular muscle.

Inter-session comparisons are also one of the important issues in EEG data analysis. Developing a robust decoding model independent of a session is a challenge because of the influence of subjects' conditions and fine changes in the electrodes' positions in each session. We prepared data recorded over three sessions to enable this cross-session analysis. For each session, we collected a dataset of uniform quality based on the classification results (see Fig. 9), because we focused on conducting all experiments in stable conditions. Researchers can analyze the decoding performance using our dataset from the entire session and also compare the decoding results of each session separately. Different approaches are also available, such as training the decoding model in a particular session and testing the model with data from independent sessions. The variance of the averaged classification accuracy in session-to-session data was low. The dataset has a reliable performance in increasing the overall classification accuracy and can be used for further researches.

Availability of supporting data and materials

The data supporting this paper, including EEG, EMG, and EOG datasets and example codes, are avaliable in the Gigascience database, GigaDB repository.

Abbreviations

BCI: brain-computer interface; EEG: electroencephalography; fNIRS: functional near-infrared spectroscopy; MEG: magnetoencephalography; SSVEP: steady-state visual evoked potential; ERP: event-related potential; MRCP:movement-related cortical potential; EMG: electromyography; EOG: electrooculography; LCD: liquid crystal display; SMR: somatosensory rhythm; ICA: Independent component analysis; CSP: common spatial pattern; RLDA: regularized linear discriminant analysis; ERSP: event-related spectral perturbation; ERD/ERS: event-related desynchronization/synchronization; sLORETA: standardized low-resolution electromagnetic tomography

Ethical Approval

This study was reviewed and approved by the Institutional Review Board at Korea University (1040548-KU-IRB-17-181-A-2).

Competing Interests

The authors declare that they have no competing interests.

Funding

This work was partly supported by Institute of Information & Communications Technology Planning & Evaluation (IITP) grant funded by the Korea government (No. 2017-0-00432, Development of Non-Invasive Integrated BCI SW Platform to Control Home Appliances and External Devices by User's Thought via AR/VR Interface), partly supported by IITP grant funded by the Korea government (No. 2017-0-00451, Development of BCI based Brain and Cognitive Computing Technology for Recognizing User's Intentions using Deep Learning), and partly supported by IITP grant funded by the Korea government (No. 2019-0-00079, Department of Artificial Intelligence, Korea University)

Author's Contributions

J.-H. Jeong, J.-H. Cho, and K.-H. Shim designed the experimental protocols and paradigms. J.-H. Jeong, B.-H. Kwon, B.-H. Lee, D.-Y. Lee, and D.-H. Lee collected the data and checked the participant's physical and mental states during the experiments. J.-H Jeong, J.-H. Cho, and S.-W. Lee revised the manuscript. All of the authors analyzed and validated the collected data technically. Also, the authors prepared the manuscript and approved the submitted manuscript.

References

1. Kaufmann T, Kübler A. Beyond maximum speed—a novel two-stimulus paradigm for brain-computer interfaces based on event-related potentials (P300-BCI). J Neural Eng 2014;11(5):056004.
2. Kim KT, Suk HI, Lee SW. Commanding a brain-controlled wheelchair using steady-state somatosensory

evoked potentials. IEEE Trans Neural Syst Rehabil Eng 2018;26(3):654–665.
3. Jeong JH, Shim KH, Kim DJ, et al. Trajectory decoding of arm reaching movement imageries for brain-controlled robot arm system. In: Proc. 41th Int. Conf. IEEE Eng. Med. Biol. Soc. (EMBC) Berlin, Germany; July 23 2019. p. 5544–5547.
4. Penaloza CI, Nishio S. BMI control of a third arm for multitasking. Sci Robot 2018;3(20):eaat1228.
5. Meng J, Zhang S, Bekyo A, et al. Noninvasive electroencephalogram based control of a robotic arm for reach and grasp tasks. Sci Rep 2016;6:38565.
6. Kwak NS, Müller KR, Lee SW. A lower limb exoskeleton control system based on steady state visual evoked potentials. J Neural Eng 2015;12(5):056009.
7. He Y, Eguren D, Azorín JM, et al. Brain–machine interfaces for controlling lower-limb powered robotic systems. J Neural Eng 2018;15(2):021004.
8. Abiri R, Borhani S, Sellers EW, et al. A comprehensive review of EEG-based brain–computer interface paradigms. J Neural Eng 2019;16(1):011001.
9. Wolpaw JR, Birbaumer N, McFarland DJ, et al. Brain–computer interfaces for communication and control. Clin Neurophysiol 2002;113(6):767–791.
10. Craik A, He Y, Contreras-Vidal JLP. Deep learning for Electroencephalogram (EEG) classification tasks: A review. J Neural Eng 2019;16(3):031001.
11. Lee MH, Fazli S, Mehnert J, et al. Subject-dependent classification for robust idle state detection using multi-modal neuroimaging and data-fusion techniques in BCI. Patt Recognit 2015;48(8):2725–2737.
12. Ang KK, Guan C. EEG-based strategies to detect motor imagery for control and rehabilitation. IEEE Trans Neural Syst Rehabil Eng 2017;25(4):392–401.
13. Tabar YR, Halici U. A novel deep learning approach for classification of EEG motor imagery signals. J Neural Eng 2016;14(1):016003.
14. Lu N, Li T, Ren X, et al. A deep learning scheme for motor imagery classification based on restricted Boltzmann machines. IEEE Trans Neural Syst Rehabil Eng 2017;25(6):566–576.
15. Won DO, Hwang HJ, Dähne S, et al. Effect of higher frequency on the classification of steady-state visual evoked potentials. J Neural Eng 2015;13(1):016014.
16. Kwak NS, Müller KR, Lee SW. A convolutional neural network for steady state visual evoked potential classification under ambulatory environment. PloS One 2017;12(2):e0172578.
17. Yeom SK, Fazli S, Müller KR, et al. An efficient ERP-based brain-computer interface using random set presentation and face familiarity. PloS one 2014;9(11).
18. Shakeel A, Navid MS, Anwar MN, et al. A review of techniques for detection of movement intention using movement-related cortical potentials. Comput Math Method M 2015;2015.
19. Jeong JH, Kwak NS, Guan C, et al. Decoding movement-related cortical potentials based on subject-dependent and section-wise spectral filtering. IEEE Trans Neural Syst Rehabil Eng 2020;.
20. Kaya M, Binli MK, Ozbay E, et al. A large electroencephalographic motor imagery dataset for electroencephalographic brain computer interfaces. Sci Data 2018;5:180211.
21. Cao Z, Chuang CH, King JK, et al. Multi-channel EEG recordings during a sustained-attention driving task. Sci Data 2019;6(1):1–8.
22. Lee MH, Kwon OY, Kim YJ, et al. EEG dataset and OpenBMI toolbox for three BCI paradigms: an investigation into BCI illiteracy. GigaScience 2019;8(5):giz002.
23. Choi GY, Han CH, Jung YJ, et al. A multi-day and multi-band dataset for a steady-state visual-evoked potential–based brain-computer interface. GigaScience 2019;8(11):giz133.
24. Shiman F, López-Larraz E, Sarasola-Sanz A, et al. Classification of different reaching movements from the same limb using EEG. J Neural Eng 2017;14(4):046018.
25. Kim IH, Kim JW, Haufe S, et al. Detection of braking intention in diverse situations during simulated driving based on EEG feature combination. J Neural Eng 2014;12(1):016001.
26. Ofner P, Schwarz A, Pereira J, et al. Upper limb movements can be decoded from the time-domain of low-frequency EEG. PloS one 2017;12(8).
27. Suk HI, Lee SW. Subject and class specific frequency bands selection for multiclass motor imagery classification. Int J Imag Syst Tech 2011;21(2):123–130.
28. Li X, Samuel OW, Zhang X, et al. A motion-classification strategy based on sEMG-EEG signal combination for upper-limb amputees. J Neuroeng Rehabil 2017;14(1):2.
29. Dai G, Zhou J, Huang J, et al. HS-CNN: a CNN with hybrid convolution scale for EEG motor imagery classification. J Neural Eng 2020;17(1):016025.
30. Kwon OY, Lee MH, Guan C, Lee SW. Subject-independent brain-computer interfaces based on deep convolutional neural networks. IEEE Trans Neural Netw Learn Syst 2019;p. 1–14.
31. Edelman BJ, Baxter B, He B. EEG source imaging enhances the decoding of complex right-hand motor imagery tasks. IEEE Trans Biomed Eng 2015;63(1):4–14.
32. Furui A, Hayashi H, Nakamura G, et al. An artificial EMG generation model based on signal-dependent noise and related application to motion classification. PloS one 2017;12(6).
33. Ma J, Zhang Y, Cichocki A, et al. A novel EOG/EEG hybrid human–machine interface adopting eye movements and ERPs: Application to robot control. IEEE Trans Biomed Eng 2014;62(3):876–889.
34. Blankertz B, Tangermann M, Vidaurre C, et al. The Berlin brain–computer interface: non-medical uses of BCI technology. Front Neurosci 2010;4:198.
35. Delorme A, Makeig S. EEGLAB: an open source toolbox for analysis of single-trial EEG dynamics including independent component analysis. Journal of neuroscience methods 2004;134(1):9–21.
36. Singh B, Wagatsuma H. A removal of eye movement and blink artifacts from EEG data using morphological component analysis. Comput Math Method M 2017;2017.
37. Blankertz B, Tomioka R, Lemm S, et al. Optimizing spatial filters for robust EEG single-trial analysis. IEEE Signal Process Mag 2007;25(1):41–56.
38. Treder MS, Porbadnigk AK, Avarvand FS, et al. The LDA beamformer: optimal estimation of ERP source time series using linear discriminant analysis. Neuroimage 2016;129:279–291.
39. Yao L, Mrachacz-Kersting N, Sheng X, et al. A Multi-class BCI based on Somatosensory Imagery. IEEE Trans Neural Syst Rehabil Eng 2018;26(8):1508–1515.
40. Tang N, Guan C, Ang K, et al. Motor imagery-assisted brain-computer interface for gait retraining in neurorehabilitation in chronic stroke. Ann Phys Rehabil Med 2018;61:e188.
41. Handiru VS, Vinod A, Guan C. EEG source space analysis of the supervised factor analytic approach for the classification of multi-directional arm movement. J Neural Eng 2017;14(4):046008.
