# Supplementary material for: Multimodal signal dataset for 11 intuitive movement tasks from single upper extremity during multiple recording sessions
Source: Gigascience. 2020 Oct 7;9(10):giaa098. doi: 10.1093/gigascience/giaa098 (PMC7539536; doi:10.1093/gigascience/giaa098)
Supplement: giaa098_GIGA-D-20-00075_Revision_1 [file giaa098_giga-d-20-00075_revision_1.pdf]

GigaScience

Multimodal signal dataset for 11 intuitive movement tasks from single upper extremity during multiple recording sessions

--Manuscript Draft--

**Manuscript Number:**	GIGA-D-20-00075R1
**Full Title:**	Multimodal signal dataset for 11 intuitive movement tasks from single upper extremity during multiple recording sessions
**Article Type:**	Data Note
**Funding Information:**	Institute of Information & Communications Technology Planning & Evaluation (IITP) grant (2015-0-00185) — Mr. Ji-Hoon Jeong<br>Institute of Information & Communications Technology Planning & Evaluation (IITP) grant (2017-0-00451) — Dr. Seong-Whan Lee<br>Institute of Information & Communications Technology Planning & Evaluation (IITP) grant (2019-0-00079) — Dr. Seong-Whan Lee
**Abstract:**	Background: Non-invasive brain-computer interfaces (BCIs) have been developed for realizing natural bi-directional interaction between users and external robotic systems. However, the communication between users and BCI systems through artificial matching is a critical issue. Recently, BCIs have been developed to adopt intuitive decoding which is the key to solving several problems such as a small number of classes and manually matching BCI commands with device control. Unfortunately, the advances in this area have been slow owing to the lack of large and uniform datasets. This study provides a large intuitive dataset for 11 different upper-extremity movement tasks obtained during multiple recording sessions. The dataset includes 60-channel electroencephalography (EEG), 7-channel electromyography (EMG), and 4-channel electrooculography (EOG) of 25 healthy subjects collected over 3-day sessions for a total of 82,500 trials across all the subjects. Findings: We validated our dataset via neuro physiological analysis. We observed clear sensorimotor de-/activation and spatial distribution related to real-movement and motor imagery (MI), respectively. Furthermore, we demonstrated the consistency of the dataset by evaluating the classification performance of each session using a baseline machine learning method. Conclusions: The dataset includes the data of multiple recording sessions, various classes within the single upper-extremity, and multimodal signals. This work can be used to i) compare the brain activities associated with real-movement and imagination, ii) improve the decoding performance, and iii) analyze the differences among recording sessions. Hence, this study, as a data note, has focused on collecting data required for further advances in the BCI technology.
**Corresponding Author:**	Seong-Whan Lee<br>Korea University<br>Seoul, KOREA, REPUBLIC OF
**Corresponding Author Secondary Information:**	
**Corresponding Author's Institution:**	Korea University
**Corresponding Author's Secondary Institution:**	
**First Author:**	Ji-Hoon Jeong
**First Author Secondary Information:**	
**Order of Authors:**	Ji-Hoon Jeong<br>Jeong-Hyun Cho<br>Kyung-Hwan Shim<br>Byoung-Hee Kwon

Powered by Editorial Manager® and ProduXion Manager® from Aries Systems Corporation

	Byeong-Hoo Lee
	Do-Yeun Lee
	Dae-Hyeok Lee
	Seong-Whan Lee
**Order of Authors Secondary Information:**	
**Response to Reviewers:**	Reply to the Comments by Reviewers<br><br>Please find the attached files named as Reply to Reviewer's Comments for better reading.<br><br>Title: Multimodal signal dataset for 11 intuitive movement tasks from single upper extremity during multiple recording sessions<br>Authors: Ji-Hoon Jeong, Jeong-Hyun Cho, Kyung-Hwan Shim, Byoung-Hee Kwon, Byeong-Hoo Lee, Do-Yeun Lee, Dae-Hyeok Lee, and Seong-Whan Lee<br><br>We would like to thank the reviewers for taking the time to carefully read the manuscript, as well as for their valuable comments and constructive suggestions. Their help enabled us to clarify many important points that we were unaware of; thus, we believe that that the manuscript quality has been improved. We attempted to incorporate as many changes as possible. For ease of review, the changes in the revised manuscript have been highlighted in blue.<br><br>==<br><br>Reviewer #1<br>The paper presents the data taken in an experiment concerning hand movement in real and imaginary motion task. The experiment included 11 different motions collecting in total 3,300 trials for each of the 25 subjects. This large dataset will be useful for other research groups to expand knowledge in the field of Neuroscience. The description of the experiment and the data is clear, however there are some comments that need to be addressed.<br><br>-Comment 1: Indeed the dataset appears to be efficient to facilitate studies about the brain kinetic patterns both in real as well as imaginary motion. This dataset can be used to train BCI simulations or do off-line analysis about kinetic brain aspects. However, since only the dataset is provided, the frequent use of BCI term (title, abstract etc) might be somewhat misleading since currently there is no interface for real-time communication between mental activities and an application<br>-Response : Thank you for your critical comment. As the reviewer mentioned, the submitted dataset can be used to train BCI simulations or perform the offline analysis of the kinetic aspects of the brain. We have also focused on collecting multimodal signals required for further high-level BCI advances. Initially, we intended the BCI term as one of the research fields. However, we completely agree with the reviewer’s comment that is no interface for the real-time communication between human and an application. The dataset provided the multimodal signal data acquired over multiple recording sessions to solve this critical BCI problem. It is one of the BCI problems, called the session-to-session problem, which is attributed to non-stationary signals. Therefore, we have changed the title of manuscript and updated the description of the BCI term in more detail. We appreciate the reviewer’s comment to improve the manuscript quality.<br>-Revision: (SECTION: Title) Multimodal signal dataset for 11 intuitive movement tasks from single upper-extremity during multiple recording sessions<br>(SECTION: Background and purpose) To the best of our knowledge, the present dataset descriptor is the first large public dataset for intuitive BCI paradigms to include multimodal signals such as EEG, EMG, and EOG signals. This study might contribute to the realization of reliable neuro-rehabilitation of patients with motor disabilities and a high-level BCI system for healthy users. Furthermore, to ensure the practicality of the BCI technology, we intend to investigate how to robustly decode motor-related intentions despite different recording sessions and subject dependency (i.e., the session-to-session problem [23, 28] and subject-independent problem [29]). However,

Powered by Editorial Manager® and ProduXion Manager® from Aries Systems Corporation

	presently, only few datasets exist to be applied to various types of real-world applications and to develop a robust neural decoding model. To overcome this challenge, this study could contribute to the development of a practical BCI system based on deep learning techniques and multi-modalities by providing a large dataset.<br><br>-Comment 2: Regarding Methods, preprocessing of the EEG could also include baseline removal as was done to EMG and referencing. Additionally, somatosensory rhythm (SMR) is usually between 13 to 15Hz, while mu is similar to alpha (8-12Hz) but mostly found over the motor cortex.<br>-Response : Thank you for mentioning the critical points. We completely agree regarding the unclear explanation of two points. First, we also adopted the baseline removal in EEG signals in the period of -0.5 s to 0 s [25], but not included in the submitted manuscript. In response to the reviewer's suggestion, we added the baseline removal of EEG signals in the Method section.<br>Additionally, we are very grateful for the reviewer's valuable comment that helped us to avoid the confusion in the manuscript. Therefore, we have modified the manuscript to provide the exact information of spectral ranges for motor-related tasks (i.e., mu-band, beta-band) included in SMR observation (13–15 Hz); please refer to [40, 41].<br>-Revision : (SECTION: Methods) Before feature extraction, we segmented a time interval of -0.5 to 4 s for performing EEG classification and also selected a baseline period as -0.5 to 0 s [25].<br>(SECTION: Methods) The data were filtered between 8 and 30 Hz (mu- and beta-bands, respectively) known as within motor-related frequency range and it could also include the spectral range for SMR observation (i.e., [13-15] Hz) [40, 41].<br><br>-Comment 3: The experimental settings took several hours each day for each participants. Is it possible that muscular and mental fatigue was included as noise in the recordings?<br>-Response : We appreciate that you indicated the experimental settings for each participant. As the reviewer was concerned, we thoroughly designed the experiment, thereby taking significant time. Therefore, during the experiment, muscular and mental fatigues could be induced. To avoid these environmental limitations, we provided sufficient breaks between the experimental tasks. Additionally, we first confirmed the physical and mental conditions of the participants through self-report. If they reported of any fatigues or unstable conditions, we reflected their opinion and made a decision to either pause or resume the experiment. Consequently, we could obtain clear signals as minimizing internal and external noises during the signal recording procedure. We have provided the details of the experimental environments in the manuscript for better comprehension.<br>-Revision : (SECTION: Experimental design) To maintain the physical and mental conditions of the subjects and thus ensure high signal quality, the subjects took sufficient breaks between each task. During the breaks, we first confirmed the physical and mental conditions of the subjects through self-report. If they reported of any inconvenient position or unstable conditions, we either adjusted the experimental environment according to their requests or halted the experiment. In the case the experiment was halted, the subjects could ask to conduct the experiment next time or not perform the experiment altogether. However, if the conditions of the subjects were good to conduct the experiment, we checked the impedances of the EEG, EMG, and EOG electrodes and injected electrolyte gel into them to maintain the impedance values below 15 kΩ. Therefore, we attempted to obtain clear signals excluding the spontaneous noise due to muscle and mental fatigue during the recording.<br><br>-Comment 4: The authors performed a validation analysis via neuro-physiological analysis, however this analysis is at preliminary level and could be enhanced by providing more details in results section, e.g. the findings related to ERDS at Fig 7 and areas in Fig 8 (still I acknowledge the main aim is the data description). Moreover, in the results section and Fig. 7 different spectral and temporal representations are showed in regard to different sessions. Were any systematic differences observed between sessions?<br>-Response : Thank you for your valuable comment. We completely agree with the insufficient validation analysis via neuro-physiological analysis. Following the

Powered by Editorial Manager® and ProduXion Manager® from Aries Systems Corporation

suggestion of the reviewer, we conducted additional data analysis for any findings related to the figures. Consequently, we could include some descriptions of brain activities during the experiment and statistical analysis for ERD/ERS values per EEG channel. Although we analyzed the data at the preliminary level, we could confirm that the dataset is well-constructed with high-quality. We have updated the manuscript by including the additional analysis and appreciate the reviewer for their suggestions to improve the manuscript quality.
-Revision : (SECTION: Results and Discussion) In Fig. 7, ERD/ERS patterns appeared during all the imagery phases (0 to 4 s), showing the same cortical activation on the mu-band across all the recording sessions. Additionally, we conducted a statistical analysis to confirm whether the subjects consistently performed MI via their single upper-extremities as instructed at the beginning. We selected typical EEG channels on the motor cortex corresponding to right-hand (channel C3), left-hand (channel C4), and foot (channel Cz) imageries [54. 55]. The mean ERD/ERS values for channel C3 contain significant differences compared with other channels (Cz and C4), as confirmed through the paired t-test. All the p-values between the channels were below 0.05 except for a few subjects.
(SECTION: Results and Discussion) Fig. 8(a) depicts the representative spatial distribution obtained using grand-average signal amplitude responses per time period [56. 57]. For subject sub2, the data for wrist pronation is presented in Fig. 8(a). We used all of EEG channels and adopted signal processing (similar to that in the pre-processing steps), such as band-pass filtering and epoch segmentation, and we also employed a baseline period. We applied the moving average of EEG amplitudes with a 200 ms interval. The topographic maps of the mean amplitudes were visualized in four temporal intervals for all the recording sessions. Therefore, the left-hemisphere of the contralateral sensorimotor region was activated while performing tasks in all the recording sessions. Hence, over time, the mean amplitude in the contralateral sensorimotor region of the left hemisphere significantly increased clearly as the subjects performed MI. Moreover, it was clearly observed that the contralateral sensorimotor regions of the most subjects were activated by MI in the 3 to 4 s. Therefore, we confirmed 0 to 2 s as the preparation period and that the contralateral sensorimotor region was appropriately activated after the first 2 s in the recording phases.
(SECTION: Results and Discussion) The main differences were observed in the supplementary motor region and premotor cortex; they indicate that the subjects satisfactorily performed MI. Since all the subjects performed MI related to the movement of the right upper limb, it can be confirmed from Fig. 8(b) that the left side region of the cortex associated with the MI is activated.
(SECTION: Results and Discussion) We evaluated the dataset quality by observing BCI classification performances (see Table 2). By using the baseline machine learning method, we confirmed that the accuracies were at least higher than chance-level accuracy for each class. The performances were validated according to tasks including arm-reaching, hand-grasping, and wrist- twisting. We computed the chance-level accuracies with a significant confidence level (a = 5%) [60] and could obtain the chance results per evaluation as 0.17 (arm-reaching), 0.34 (hand-grasping), and 0.51 (wrist-twisting). Table 2 represents the classification accuracies with the standard deviation for each subject and recording session. Because our dataset was recorded over three different sessions, it allows for further research related to BCI-calibration problems. According to our classification result obtained using the baseline decoding method and conventional approach, some subjects showed a significant change in classification results when the session as changed; however, the other subjects showed similar classification accuracies over different sessions.

-Comment 5: Regarding classification performance, it is not clear, what were the features? Finally, while Fig. 9 provides all the information, a separate summary of classification results (a table or larger version of the “average” columns) could be better for the reader get an overview. Finally, the accuracies are relatively low, however it is acceptable at this level since the aim is to prove the consistency of the dataset.
-Response : Thank you for the suggestion of providing more information on the classification performance in Fig. 9. Accordingly, we have revised the classification performance, of each subject, for each class from the figure to the table (i.e., Table 2) to enable better understanding. We also modified the manuscript in detail about the classification performances using the baseline machine learning method (CSP+RLDA).

Powered by Editorial Manager® and ProduXion Manager® from Aries Systems Corporation

Additionally, the grand-average classifications ac- curacies were similar to the chance-level accuracy. As the reviewer mentioned, we could prove the consistency and quality of the dataset for each recording session through preliminary data validation. In this study, we collected large dataset for multimodal signals, so other researchers could try novel approaches in future studies to overcome various types of limitations. Following the comment by the reviewer, we have revised the manuscript more in detail in the "Results and Discussion" section.
-Revision : (SECTION: Results and Discussion) We evaluated the dataset quality by observing BCI classification performances (see Table 2). By using the baseline machine learning method, we confirmed that the accuracies were at least higher than chance-level accuracy for each class. The performances were validated according to tasks including arm-reaching, hand-grasping, and wrist- twisting. We computed the chance-level accuracies with a significant confidence level (a = 5%) [60] and could obtain the chance results per evaluation as 0.17 (arm-reaching), 0.34 (hand-grasping), and 0.51 (wrist-twisting). Table 2 represents the classification accuracies with the standard deviation for each subject and recording session. Because our dataset was recorded over three different sessions, it allows for further research related to BCI-calibration problems. According to our classification result obtained using the baseline decoding method and conventional approach, some subjects showed a significant change in classification results when the session as changed; however, the other subjects showed similar classification accuracies over different sessions.

-Comment 6: Some proofreading and polishing in the English language is required. In addition, in EMG preprocessing the ~ symbol can change to "to".
-Response : Thank you for your valuable comment. We have conducted proofreading and polished the revised manuscript for the English language. Additionally, we have changed the "~"symbol to "to" in the manuscript.
-Revision : (SECTION: Methods) We segmented a time interval of -0.5 to -4 s for EMG data analysis. We selected the interval of -0.5 to 0 s for as the baseline period.

-Comment 7: Since classification is performed, in the introduction section, a few machine learning review papers about BCI can be mentioned such as:
Kakkos I., Miloulis ST., Gkiatis K., Dimitrakopoulos G.N., Matsopoulos G.K. (2020). Human-Machine Interfaces for Motor Rehabilitation.
Maglogiannis I., Brahnam S., Jain L. (eds) Advanced Computational Intelligence in Healthcare-7. Studies in Computational Intelligence, vol 891. Springer, Berlin, Heidelberg.
Abiri, R., Borhani, S., Sellers, E. W., Jiang, Y., Zhao, X. (2019). A comprehensive review of EEG-based brain-computer interface paradigms," Journal of Neural Engineering, vol. 16, no. 1, p. 011001, Jan. 2019.
-Response : Following the suggestion by the reviewer, we have modified the manuscript for enhanced clarity by referencing some other papers in the "Background and purpose" section.
-Revision: (SECTION: Background and purpose) A noninvasive BCI commonly employs electroencephalography (EEG) signals to decode user intentions [8-11] because the EEG-based BCI system offers lower risk, lower cost, and more convenient than other non-invasive BCI paradigms (e.g., functional near-infrared spectroscopy (fNIRS) [12]).

==

Reviewer #2
The authors provide a dataset consisting of EEG, EMG and EOG recordings from 25 subjects performing real and imaginary movements of the right arm and hand across 3 different sessions. This dataset can provide the community with an opportunity for method evaluations. The number of tasks that each subject performs together with the number of trials for each subject are substantial but the classification accuracy is rather low compared to the existing literature. Overall the paper is well written and covers most of the information explaining and evaluating the provided dataset. However, a detailed comparison with the existing datasets (explaining how the provided dataset is novel/different from the existing ones) is missing.

Powered by Editorial Manager® and ProduXion Manager® from Aries Systems Corporation

	Here are some comments and suggestions to help further clarify the manuscript:<br><br>-Comment 1: Please explain in more details how the provided dataset is novel/different from the existing open-access BCI datasets.<br>-Response : Thank you very much for letting us know that we did not sufficiently explained the novelty and difference of our dataset relative to the existing BCI datasets. The provided multimodal signal dastaset could contribute to developing a high-level BCI system and supporting the practicality of the BCI technology. Furthermore, to realize more advanced in the BCI technology, a large dataset is essential owing to the requirement of various types of approaches such as deep learning or multimodal signals fusion. Hence, this dataset can be useful for solving related BCI problems including the session-to-session problem, subject-independent model construction, and multimodal signals decoding. Following the critical point by the reviewer, we have provided more details in the revised manuscript.<br>-Revision: (SECTION: Background and purpose) Eventually, the multimodal signal dataset became sufficiently large for the BCI experiment because it now included data acquired from 82,500 trials performed for all the subjects (i.e., 3,300 trials were collected per subject).<br>(SECTION: Background and purpose) To the best of our knowledge, the present dataset descriptor is the first large public dataset for intuitive BCI paradigms to include multimodal signals such as EEG, EMG, and EOG signals. This study might contribute to the realization of reliable neuro-rehabilitation of patients with motor disabilities and a high-level BCI system for healthy users. Furthermore, to ensure the practicality of the BCI technology, we intend to investigate how to robustly decode motor-related intentions despite different recording sessions and subject dependency (i.e., the session-to-session problem [23, 28] and subject-independent problem [29]). However, presently, only few datasets exist to be applied to various types of real-world applications and to develop a robust neural decoding model. To overcome this challenge, this study could contribute to the development of a practical BCI system based on deep learning techniques and multi-modalities by providing a large dataset.<br>(SECTION: Results and Discussion) Additionally, more advanced analyses can be attempted because we have constructed a database that includes not only the EOG and EMG data but also the EEG data. For example, the EOG data might be used to remove the noise due to explicit eye movement from the EEG data. Additionally, the EMG data may demonstrate the integrity of the EEG data by showing that no movement-related interference was present in the EMG data in the analysis and MI tasks associated with the EEG. In this work, we recorded the signals' data using three modalities, namely, EEG, EOG, and EMG. Additionally, we collected data from 25 subjects and divided the experiment into three sessions to prepare the dataset. We also provided the spectral representation and time-spatial distribution of a representative subject according to multiple recording sessions. Generally, we confirmed that the data variability among each recording session did not show any significant differences in our dataset (i.e., $p<0.05$). Furthermore, we confirmed that the classification accuracy per task was slightly higher than the chance-level accuracy using the baseline method. Conversely, despite the difficult tasks involved in the experiments, the subjects successfully focused on the experiments so that we could obtain high-quality data. In the future, the users of this dataset can contribute to increasing the present classification accuracy using their novel methodology.<br>(SECTION: Results and Discussion) Inter-session comparisons are also important topics in BCI experiments. Since the BCI systems are recalibrated at the beginning of each recording session, this procedure becomes time-consuming and thus may limit the adoption of BCI systems for long-term daily usage [63]. Furthermore, we recorded data over three sessions to enable cross-session analysis. For each session, we collected a dataset of uniform quality on the basis of classification results (see Table 2), as we focused on conducting all the experiments under stable conditions. Researchers can analyze the decoding performance using our dataset from the entire session and they can also compare the decoding results of each session with each other. Different approaches are also available, and they include training the decoding model in a particular session and testing the model using data from independent sessions on the basis of the principle of transfer learning in BCI, as done in [64]. Accordingly, creating a session-independent BCI decoding model is critical to establishing a practical BCI system such as biometric authentication system [65] and brain-controlled AR/VR system [66]. Therefore, our experimental data can be useful for

studies to build session-independent decoding models.

-Comment 2: Page 2, paragraph 2: it is unclear what the authors mean by ‘intuitive decoding without artificial interaction’. The provided citations do not contain information about this particular terminology. This term is also used in the abstract. Please clarify.
-Response : Thank you very much for letting us know that we included many confusing sentences in the manuscript. We also completely agree with the reviewer’s confusion about the needs for intuitive decoding. We have modified the manuscript using easier words and provided more details with examples. Additionally, we mentioned the studies of decoding of upper-extremity movement from the same limb using EEG signals such as arm reaching to multi-direction and various types of hand movements in the provided citations. The aim of these studies has also presented the possibility of intuitive decoding using a single-arm and analyze EEG signals during the tasks. We also revised these references to be make them clearer.
-Revision : (SECTION: Background and purpose) The recent advances in BCI systems have been focused on topics ranging from intuitive EEG decoding to directly matching the interaction between user intention and device feedback for real-world environments [20, 26]. For example, to control a neuro-prosthetic arm using typical BCI paradigms, we temporarily matched BCI commands with robotic arm motions (e.g., MI for both hands = grasping motion of the robotic hand). However, this unintended artificial matching suffers from several constraint limitations, such as the small number of restrictive classes for communicating with devices and inflexible user training due to unintuitive commands in real-world scenarios [7, 13, 27]. For example, if a robotic hand performs the grasping motion, the users should also imagine the hand grasping motion the same as the robotic hand motion to enhance the natural decoding experience [27].

-Comment 3: Page 2, paragraph 2: This sentence is not clear: “For example, to control a neuro-prosthetic arm using typical paradigms, we temporally matched BCI commands with robotic arm motions (e.g., motor imagery for both hands = grasping motion of robotic hand).” What is temporal matching? Please provide appropriate citations as well.
-Response : We appreciate your indicating the typographical errors. We have revised the words from ”temporal” to ”temporarily” and also modified the sentence to be clearer as Comment 2.
-Revision: (SECTION: Background and purpose) For example, to control a neuro-prosthetic arm using typical BCI paradigms, we temporarily matched BCI commands with robotic arm motions (e.g., MI for both hands = grasping motion of the robotic hand).

-Comment 4: Why did the authors choose the movements of the right arm only? Why not both?
-Response : Thank you for your critical comment. In our experiment, we asked the subjects to conduct 11 different upper extremity movements as experimental tasks. The tasks comprised single upper extremity movement only. As mentioned in a related work [27], one of the BCI advances is decoding different tasks performed from the upper extremity by using EEG signals. Compared with typical BCI paradigms that use left/right/foot imagery, it could increase the number of BCI control possibilities. We have focused on decoding various types of movement from single-arm and thus recruited the participants with dominant arms on the right side, and not ambidextrous participants, according to the related studies [27]. In response to your comment, we have added the reason why we chose the movements of the right arm only.
-Revision : (SECTION: Experimental design) The experiment was designed to quantitatively acquire data related to the 11 different upper-extremity movements for both real-movement and MI tasks. The subjects conducted the experimental tasks using the same limbs. Decoding different tasks related to the same limb by using EEG signals could increase the number of possibilities of controlling the BCI system compared with typical somatosensory rhythm (SMR)-based BCIs which often only detected left-/right-hand and foot imagery [27].

-Comment 5: Are the subjects all right-handed?

Powered by Editorial Manager® and ProduXion Manager® from Aries Systems Corporation

	-Response : Thank you for letting us know about our omission of information of the subjects. We have added the information of the dominant arms for subjects as "All-right handed."<br>-Revision : (SECTION: Experimental design) Twenty-five subjects (all-right handed, S1-S25, aged 24-32 years, 15 males and 10 females) who were naive BCI users participated in the experiments.<br><br>-Comment 6: What do the authors mean by the following sentence: "however, unsatisfactory decoding performance was a significant problem, predominantly when testing in the background of MI and in a session independent situation"?<br>-Response : Thank you for the critical comment. We completely agree with the unclear sentence and have removed from the revised manuscript to convey our intention meaning.<br><br>-Comment 7: Page 2, experimental design: How did the authors check for the physical and mental states of the subjects? Was this self-reported by the subjects or did a physician evaluate them prior to participating in the experiment?<br>-Response : Our response to this comment is the same as our response to Comment 3 of Reviewer #1. We completely agree with your indicating the experimental settings for each participant. As the reviewer was concerned, we thoroughly designed the experiment, thereby taking a significant time. Therefore, during the experiment, muscular and mental fatigues could be induced. To avoid these environmental limitations, we provided sufficient break times between the experimental tasks. Additionally, we first confirmed the physical and mental conditions of the participants through self-report. If they reported of any fatigues or unstable conditions, we either adjusted the experimental settings as per their opinion or made a decision to pause or resume the experiment. Consequently, we could obtain clear signals as minimizing internal and external noises during the signal recording. We have added the description of the experimental environment in more detail for better comprehension.<br>-Revision : (SECTION: Experimental design) To maintain the physical and mental conditions of the subjects and thus ensure high signal quality, the subjects took sufficient breaks between each task. During the breaks, we first confirmed the physical and mental conditions of the subjects through self-report. If they reported of any inconvenient position or unstable conditions, we either adjusted the experimental environment according to their requests or halted the experiment. In the case the experiment was halted, the subjects could ask to conduct the experiment next time or not perform the experiment altogether. However, if the conditions of the subjects were good to conduct the experiment, we checked the impedances of the EEG, EMG, and EOG electrodes and injected electrolyte gel into them to maintain the impedance values below 15 kΩ. Therefore, we attempted to obtain clear signals excluding the spontaneous noise due to muscle and mental fatigue during the recording.<br><br>-Comment 8: The monitor was placed about 60 cm away from the subjects. This in my opinion is a very short distance as an average adult's extended arm length is beyond 60 cm. Why was this distance selected? How do the authors ensure comfortable extension of the arm during the experiment?<br>-Response : Thank you for mentioning a very critical point. We agree with your remark that the submitted manuscript could cause confusion owing to the typographical error. We have revised the manuscript from 60 cm to 80 cm according to the reference paper.<br>-Revision : (SECTION: Experimental design) During the experiments, each subject was comfortably seated in a chair with armrests facing the front of an LCD monitor, approximately 80 (±5)cm away from each other [30].<br><br>-Comment 9: Figure 4: For better clarification, please explicitly mention that the EOG channels were channels numbered 32-35 out of the 64-channel acticap montage.<br>-Response : Following the suggestion by the reviewer, we have modified Fig. 4 for including the channel numbers of EOG not only in the EOG section but also in the EEG section. Additionally, we have mentioned that the EOG channels were numbered as 32–35 out of the 64-channel actiCAP montage in the caption of Fig. 4.

Powered by Editorial Manager® and ProduXion Manager® from Aries Systems Corporation

-Revision : (SECTION: Data Records) Figure 4. Data configuration for 60 EEG, 7 EMG, and 4 EOG channels. Specifically, 4 EOG channels were numbered as 32-35 out of the 64-channel actiCAP montage.

-Comment 10: In the experiment, when were the subjects notified so as to perform motor imagery or actual movement? Was it only after the visual cue?
-Response : We appreciate your indicating the insufficient explanation of the experimental paradigm. In our experiment, the subjects were asked to perform motor imagery or real movement tasks following the text sign, as depicted in Fig. 2. We have modified the manuscript by including the exact experimental instructions and also added the description in Fig. 2.
-Revision : (SECTION: Experimental design) Upon changing the visual cue to a text sign as “Movement execution” and “Movement imagery”, the subjects performed the corresponding tasks during 4 s.

-Comment 11: This sentence is missing a verb: “In the motor imagery tasks, the subjects only one of the three grasping motions per trial, randomly.”
-Response : Thank you for your valuable comment. We have modified the sentence by including the relevant verb in the revised manuscript.
-Revision : (SECTION: Experimental design) In the MI tasks, the subjects performed only one of the three grasping motions per trial, randomly.

-Comment 12: Why did the authors use a notch filter while recording? Given that the data is recorded for offline analysis, this does not seem to be necessary. Also, does this mean that the recorded data is not raw but notch filtered? Please provide the specifications of the notch filter?
-Response : Thank you for letting us know that the manuscript needs a more detailed description. We missed the explanation for the role of a notch filter during the signal recording in BCI. The raw signals, upon acquiring through experiments, were affected by external environment noise due to their sensitive characteristics. Generally, we conduct BCI experiments with some constraints such as an intensive place without electrical contamination. Hence, the investigators have normally performed the experiment in their laboratory environment. Furthermore, they have used a 50- or 60Hz notch filter to reduce the effect of external electrical noises (e.g., DC noise due to power supply, scan rate of a monitor display, and frequency of the fluorescent lamp) in raw signals. In our experiment, we also applied a 60Hz notch filter to acquire clean raw signals. We completely agree that we provided an insufficient explanation for signal recording, and thus we have added the detailed description with the corresponding reference paper in the revised manuscript.
-Revision : (SECTION: Data Records) The EEG data were recorded in conjunction with an EEG signal amplifier (BrainAmp, BrainProduct GmbH, Germany), sampled at 2,500 Hz. Additionally, we applied a 60 Hz with a notch filter to reduce the effect of external electrical noises (e.g., DC noise due to power supply, scan rate of the monitor display, and frequency of the fluorescent lamp) in raw signals [21, 32, 33].
(SECTION: Data Records) The signals were captured at a sampling rate of 2,500 Hz with a 60 Hz notch filter same as the setting used to record the EEG signals.

-Comment 13: Why did the authors choose to put the reference electrode of the EMG recording on the elbow? I believe the electrode would be affected by the joint and skin movements during the reach and would not be able to provide a reliable reference.
-Response : Thank you for your valuable comment. EMG signals shared the same ground, and reference channels as EEG signals owing to using the same signal amplifier. Therefore, the ground and reference channels are located on the forehead and scalp, which are not affected by the arm movement. The attached channel of the elbow is the additionally attached reference channel, which is the attachment position of the reference channel frequently used in EMG studies related to the movements of the elbow, hand and wrist. Therefore, if necessary, researchers made this channel available as a reference to EMG. In response to the comment by the reviewer, we have modified the manuscript with sufficient explanation.
-Revision : (SECTION: Data Records) The ground and reference were recorded in Fpz

and FCz, respectively, which are the same as the EEG and EOG signals. The last electrode was placed on the elbow of the right arm which is a non-muscle movement area, as an alternative reference signal [36].

Comment 14: Did the EMG electrodes use the same GND as the EEG and EOG channels?
Response : Our response to this comment is the same as our response to Comment 13. The EMG electrodes used the same GND as those used by the EEG and EOG electrodes. We have also updated the description in the revised manuscript.
Revision : (SECTION: Data Records) The ground and reference were recorded in Fpz and FCz, respectively, which are the same as the EEG and EOG signals. The last electrode was placed on the elbow of the right arm which is a non-muscle movement area, as an alternative reference signal [36].

-Comment 15: It seems that there is a typo where the authors explain which EOG channels were used for horizontal and vertical eye movements. EOG1 and 4 should be for horizontal and EOG2 and 3 for vertical eye movements. Please revise.
-Response : Thank you for your critical comment. We have revised the sentence appropriately as EOG1 and EOG4 for horizontal eye movements and EOG2 and EOG3 for vertical eye movements.
-Revision : (SECTION: Data records, EOG signals) The electrodes EOG1 and EOG4 were used to record horizontal eye movements, while EOG2 and EOG3 were used to record vertical movements [37].

-Comment 16: What sort of pre-processing makes the .mat files? Are the relevant pre-processing files also provided?
-Response : Thank you for mentioning the data conversion issue. We have discussed how the users can access the dataset more conveniently. In signal processing, the MATLAB software was generally used for data analysis. Therefore, we decided to provide the datasets that included not only raw .eeg files but also converted .mat files that could be immediately accessed in the MATLAB software. Therefore, we converted the raw .eeg data to .mat data which included information including trigger mark information, channel configuration, and epoch segmentation, without any relevant pre-processing step. We have modified the manuscript to be more clear.
-Revision : (SECTION: Data records) Additionally, to enable easy data access for the users, we provided the dataset after converting the .eeg file to .mat file in the "Converted data" folder. The converted .mat file includes some information such as trigger mark information, channel configuration, epoch segmentation, and etc.

-Comment 17: Do the authors mean to say 'basic pre-processing steps in' where they say "basis pre-processing step on"?
-Response : Thank you very much for letting us know that we included confusing sentences in the submitted manuscript. We have removed the confusing phrases from the sentences clarified the text. The revision for the Comment #17 is the same as the revision for the Comment #16.
-Revision : (SECTION: Data records) Additionally, to enable easy data access for the users, we provided the dataset after converting the .eeg file to .mat file in the "Converted data" folder. The converted .mat file includes some information such as trigger mark information, channel configuration, epoch segmentation, and etc.

-Comment 18: Visualization seems to be channels by time and not time by channel (as depicted in Figure 5). Please revise.
-Response : Thank you for your critical comment. We have revised the sentence from "time by channels" to "channels by time" for a representative subject.
-Revision: (SECTION: Data records) The raw signals were visualized as channels through the time representation for the representative subject named sub8.

-Comment 19: What was the ICA pipeline (any channel rejection, re-referencing, etc.)?

	Which ICA algorithm was used for the decomposition?<br>-Response : Thank you for indicating the insufficient explanation about ICA in pre-processing. For artifact rejection associated with apparent eye blinking, we have adopted infomax ICA methods. The basic principle of the ICA algorithm is to decompose some independent components, as depicted in Fig. 5. Additionally, the ICs with patterns similar to the EOG channels (i.e., horizontal and vertical eye movements) have been removed as contaminated ICs. The remaining ICs were projected back into the scalp channel space to be reconstructed as the corrected EEG data. Consequently, we could obtain clean EEG data without other contamination for data analysis. We have modified the manuscript more in detail by adding a reference paper for better comprehension.<br>-Revision : (SECTION: Methods) To obtain corrected EEG data, we removed the contamination factors using the infomax ICA [43], which is used to decompose brain signals into statistically independent components (ICs). From various types of ICA methods, we adopted the ICA with the infomax algorithm as it could robustly remove artifacts, such as eye and head movement artifacts from the EEG data robustly [44]. The EEG data were transformed by the ICA mixing matrix. The contaminated ICs with patterns similar to the EOG channels (i.e., horizontal and vertical eye movements) were removed. Subsequently, the remaining ICs were projected back into the scalp channel space to be reconstructed as the corrected EEG data (see Fig. 5).<br><br>-Comment 20: Please remove the term 'shift' in "zero-phase shift bandpass filter".<br>-Response : Following the suggestion by the reviewer, we have removed the term 'shift'<br>from the sentence. We appreciate the opinion of the reviewer aimed at better understanding of the reader.<br>-Revision: (SECTION: Methods) In this work, the EMG signals were pre-processed from 10–500 Hz with a Butterworth 5th zero-phase bandpass filter [34, 49].<br><br>-Comment 21: Figure 6: It seems that the location of the EMG electrodes does not capture the wrist movement very well. Please explain. Some sort of statistical analysis is necessary to compare the time series in the two conditions (real movement and motor imagery) within subject and/or across participants.<br>-Response : Thank you for your critical comment. Actually, one of the goals of our experiment was to record the kinematic signals associated with rotational wrist movements (leftward and rightward). Therefore, we have decided that the EMG signals from the forearm muscles (extensor carpi ulnaris, extensor digit, flexor carpi radialis, and flexor carpi ulnaris) would suffice to represent wrist rotation. This action does not require the direct movement of the wrist muscles because it does not involve wrist bending. Additionally, we have updated the description of the reasons why the EMG signals associated with wrist-twisting were not clear compared with the signals associated with other actions.<br>-Revision : (SECTION: Methods) EMG signal patterns contains important information regarding muscle activation and noise. For example, the noise due to heart beat and other unrelated movements reduces the quality of EMG signals. While the subjects performed the wrist-twisting task, the amplitude scale was reduced as compared with other work, so that noise information could be confirmed to be displayed. Meanwhile, in the real-movement tasks, the EMG signals could show the activation while the subjects were performing the upper-extremity movement task. Notably, the signal amplitude of channel EMG6 is higher than those of the other channels. Since biceps are relatively bigger than other muscles, a peak phenomenon occurred [50]. Particularly, biceps are used the most in bending the arm. The EMG signals featured a large signal amplitude, as more muscle activity increases the signal amplitude. Consequently, the EMG signal amplitude was large for the arm-reaching and hand-grasping tasks, but small for the wrist-twisting task [51].<br><br>-Comment 22: Page 7, paragraph 1:what is the classification done with respect to? Different movements? How many classes at a time? How is the shrinkage parameter estimated?<br>-Response : We appreciate that the reviewer has indicated the evaluation issue regarding the classification performance. We confirm that sufficient description was not

provided for evaluating the classification performance. A regularized linear discriminant analysis (RLDA) classifier has been adopted for multi-class classification. It is generally used as a baseline algorithm for the decoding of motor imagery and execution using EEG signals [47, 48]. In the classification procedure, we have classified the multi-class according to each experimental task. Conversely, we have evaluated the classification performance for each different movement, separately, such as arm-reaching along six direction (6–class), hand-grasping of three objects (3-class), and wrist-twisting with two different motions (2–class). The RLDA classifier was trained using common spatial pattern (CSP) features with regularized terms by employing optimal shrinkage parameters. Essentially, shrinkage performs regularization to improve the estimation of covariance matrices, where the training samples are fewer than features. Therefore, during the training period, the optimal shrinkage parameter was automatically estimated with maximum covariance between the classes. We have modified the manuscript more in detail by using descriptions and citations about the classification procedures.
-Revision : (SECTION: Methods) We trained the RLDA classifier by adding a regularization term to the covariance matrix using the optimal shrinkage parameters [47, 48]. Essentially, shrinkage performs regularization to improve the estimation of covariance matrices where the training samples are fewer than features. Therefore, during the training period, the optimal shrinkage parameter was automatically estimated with the maximum covariance between classes. In the classification procedure, we classified the multi-class according to each different experimental task, separately, such as arm-reaching along six directions (6-class), hand-grasping of three objects (3-class), and wrist-twisting with two different motions (2-class), as depicted in Fig. 3.

-Comment 23: Please support the following sentences by providing appropriate statistical tests: “The ERD patterns can be seen during motor preparation, execution, and imagery as the correlations in an activated cortical area. ERS can be observed after the imagery or execution of movement over the same area.”
-Response : Thank you for your important comment. We have previously included the characteristics of ERD/ERS patterns with the reference paper. Furthermore, in response to the suggestion by the reviewer, we have conducted a statistical analysis using mean ERD/ERS values according to the representative channels. Therefore, we have selected typical EEG channels on the motor cortex, corresponding to the right-hand (C3), left-hand (C4), and foot (Cz) imageries [54, 55]. The mean ERD/ERS values for the C3 contain significant differences compared with the other channels (Cz and C4), as confirmed through a paired t-test ($p<0.05$) except for a few subjects. Through the statistical analysis, we could demonstrate whether the subjects attended the experiment and imagined the experimental task using the right-arm only.
Revision : (SECTION: Results and Discussion) Additionally, we conducted a statistical analysis to confirm whether the subjects consistently performed MI via their single upper-extremities as instructed at the beginning. We selected typical EEG channels on the motor cortex corresponding to right-hand (channel C3), left-hand (channel C4), and foot (channel Cz) imageries [54, 55]. The mean ERD/ERS values for channel C3 contain significant differences compared with other channels (Cz and C4), as confirmed through the paired t-test. All the p-values between the channels were below 0.05 except for a few subjects.

-Comment 24: Figure 7: how were the scalp topographies calculated? Was this averaged across many trials? How many and which ones?
-Response : Thank you for indicating the insufficient explanation of the scalp topographies in Fig. 8(a). We have represented spatial distributions using the values of grand-average signal amplitudes across all the trials. We have used all the EEG channels and adopted signal processing in a manner similar to the pre-processing steps, such as implementing band-pass filtering and epoch segmentation, and we have also employed a baseline period. We have also applied the moving average of amplitudes with a 200 ms interval. The topographic maps were visualized in four temporal intervals for all the recording sessions. We confirm that the left-hemisphere of the contralateral sensorimotor region was activated while performing the tasks in all the recording sessions for a representative subject named sub2.
-Revision : (SECTION: Results and Discussion) Fig. 8(a) depicts the representative

spatial distribution obtained using grand-average signal amplitude responses per time period [56, 57]. For subject sub2, the data for wrist pronation is presented in Fig. 8(a). We used all of EEG channels and adopted signal processing (similar to that in the pre-processing steps), such as band-pass filtering and epoch segmentation, and we also employed a baseline period. We applied the moving average of EEG amplitudes with a 200 ms interval. The topographic maps of the mean amplitudes were visualized in four temporal intervals for all the recording sessions. Therefore, the left-hemisphere of the contralateral sensorimotor region was activated while performing tasks in all the recording sessions.

-Comment 25: Please add similar analysis (Figures 6-9) for all subjects and add them to the data directory or as an appendix to the manuscript.
-Response : Thank you for your valuable comment. If the manuscripts and supplementary documents contain all the figures for each subject and for each analysis (i.e., EMG activation, EEG scalp topographies, event-related spectral perturbation (ERSP), and source imaging), it would be inconvenient for users to access the data analysis owing to a significant number of documents. Therefore, we have decided to provide some codes with respect to the analysis for all the subjects so that the users can easily access the dataset analysis. We added those codes in ‘7 Revision code’ folder at the GigaDB repository. We really appreciate your considering the quality improvement of the manuscript of data notes.

-Comment 26: Figure 8: what is the scale of the colored regions? Which subject does this data pertain to?
-Response : Thank you for your valuable comment. In response to the suggestion by the reviewer, we have revised Fig. 8(b) by adding the scale of colored regions with the information of the subjects. We have also updated the manuscript more in detail by adding the descriptions of the source images obtained by the statistical analysis to enable better understanding of the reader. The source images were visualized by the significant differences by calculating p-values for the spatial distribution between the baseline period (-0.5 to 0 s) and motor imagery period (0 to 4 s). The yellow colors indicated the p-values below 0.01, and the red colors those below 0.05 [59].
-Revision : (SECTION: Results and Discussion) We visualized the activated regions of the brain for each task and showed them in terms of the horizontal, sagittal, and coronal planes, as shown in Fig. 8(b). The source images were visualized by the significant differences by calculating the p-values for the spatial distribution between the baseline period (-0.5 to 0 s) and MI period (0 to 4 s). The yellow colors indicate the p-values below 0.01, and the red colors those below 0.05 [59].

-Comment 27: Please update the reported chance levels with a confidence interval depend- ing on the number of available trials. See the following paper: Muller-Putz, Gernot, et al. “Better than random: a closer look on BCI results.” International Journal of Bioelectromagnetism 10.ARTICLE (2008): 52-55.
-Response : We appreciate that the reviewer indicated the issues regarding the chance-level accuracy of the dataset. We have updated the manuscript regarding the chance level accuracy with a confidence interval motivated by the suggested citation [60]. We have computed the chance-level accuracies with a significant confidence level (a = 5%) and could obtain the chance results for each evaluation as 0.17 (arm-reaching), 0.34 (hand-grasping), and 0.51 (wrist-twisting). Thank you for letting us know the exact information.
-Revision : (SECTION: Results and Discussion) We evaluated the dataset quality by observing BCI classification performances (see Table 2). By using the baseline machine learning method, we confirmed that the accuracies were at least higher than chance-level accuracy for each class. The performances were validated according to tasks including arm-reaching, hand-grasping, and wrist-twisting. We computed the chance-level accuracies with a significant confidence level (a = 5%) [60] and could obtain the chance results per evaluation as 0.17 (arm- reaching), 0.34 (hand-grasping), and 0.51 (wrist-twisting).

-Comment 28: What is the classification procedure? How are the train and test sets

selected?
-Response : Thank you for mentioning these important issues for classification. In response to your comment, we added the description of the training set and test set selection. In the fair evaluation, initially, we adopted 10x10 cross-validation so that we partitioned the data samples as equal sizes into 10 subsets. Therefore, one subset was selected as a test set and the other subsets were used for the training set. In addition, we conducted 10 times with each of the 10 sub- sets used once as the test dataset to avoid variability problems in performance evaluation.
-Revision : (SECTION: Methods) In the classification procedure, we classified the multi-class according to each different experimental task, separately, such as arm-reaching along six directions (6-class), hand-grasping of three objects (3-class), and wrist-twisting with two different motions (2-class), as depicted in Fig. 3. We applied 10x10-fold cross-validation for fair performance measurement so that we partitioned the data samples as equal sizes into 10 subsets. One of the subsets was selected as the test dataset and the remaining others as the training datasets. The cross-validation process was conducted 10 times with each of the 10 subsets used once as the test dataset to avoid variability problems in performance evaluation. The evaluation was estimated using all recorded classes simultaneously over all the recording sessions. Table 2 presents the averaged evaluation results obtained by estimating the classification performance of each arm-reaching (6-class), hand-grasping (3-class), and wrist-twisting (2- class) task.

-Comment 29: Figure 9: Please provide the same range (0-1) for the y-axis in all plots. Also, what do the bar heights and the error bars represent?
-Response : Thank you for the suggestion of indicating more information (i.e., scale of the figure, and error bar of performance) on the classification performance in Fig. 9. Our response to this comment is same as our response to Comment 5 of Reviewer #1. Therefore, we have revised the classification performance, of each subject, for each class from the figure to the table (i.e., Table 2) to enable better understanding.

-Comment 30: Majority of the subjects show classification accuracy very close to chance level. Please explain.
-Response : Thank you for the valuable comment. The submitted manuscript is a data note, not a research article. Therefore, we have focused on constructing a dataset that could contribute to BCI-based studies, and we have also included the novelty and difference of our dataset compared with the conventional dataset. Consequently, we could prove the consistency and quality of our dataset for each recording session through preliminary data validation. In this study, we present the objective and large dataset for multimodal signals, and researchers could overcome various types of study limitations using novel approaches in future studies (e.g., classification performance enhancement, session-to- session transfer BCI, and etc).
-Revision : (SECTION: Background and purpose) This study might contribute to the realization of reliable neuro-rehabilitation of patients with motor disabilities and a high-level BCI system for healthy users. Furthermore, to ensure the practicality of the BCI technology, we intend to investigate how to robustly decode motor-related intentions despite different recording sessions and subject dependency (i.e., the session-to-session problem [23, 28] and subject- independent problem [29]). However, presently, only few datasets exist to be applied to various types of real-world applications and to develop a robust neural decoding model. To overcome this challenge, this study could contribute to the development of a practical BCI system based on deep learning techniques and multi-modalities by providing a large dataset.
(SECTION: Results and Discussion) Furthermore, we confirmed that the classification accuracy per task was slightly higher than the chance-level accuracy using the baseline method. Conversely, despite the difficult tasks involved in the experiments, the subjects successfully focused on the experiments so that we could obtain high-quality data. In the future, the users of this dataset can contribute to increasing the present classification accuracy using their novel methodology.

-Comment 31: The authors mention that “We also analyzed whether the data were inter-session dependent or independent;” where in the manuscript is this explained?
-Response : We agree with your critical remark that the submitted manuscript could

Powered by Editorial Manager® and ProduXion Manager® from Aries Systems Corporation

cause confusion. For better comprehension, we have excluded the concerned sentence from the revised manuscript. Instead, regarding the description of an inter-session problem, we have updated the manuscript more in detail in the 'Results and Discussion' section as described below. Thank you for your valuable comment.
-Revision: (SECTION: Results and Discussion) Inter-session comparisons are also important topics in BCI experiments. Since the BCI systems are recalibrated at the beginning of each recording session, this procedure becomes time-consuming and thus may limit the adoption of BCI systems for long-term daily usage [63]. Furthermore, we recorded data over three sessions to enable cross-session analysis. For each session, we collected a dataset of uniform quality on the basis of classification results (see Table 2), as we focused on conducting all the experiments under stable conditions. Researchers can analyze the decoding performance using our dataset from the entire session and they can also compare the decoding results of each session with each other. Different approaches are also available, and they include training the decoding model in a particular session and testing the model using data from independent sessions on the basis of the principle of transfer learning in BCI, as done in [64]. Accordingly, creating a session-independent BCI decoding model is critical to establishing a practical BCI system such as biometric authentication system [65] and brain-controlled AR/VR system [66]. Therefore, our experimental data can be useful for studies to build session-independent decoding models.

-Comment 32: Please explain in the discussion section how the rather low classification accuracy and the fact that the experiment only focused on right arm/hand movements may limit the application of the provided dataset.
-Response : We have previously mentioned the issues related to the moderately low classification accuracy and those related to the experiment being performed using only a single-arm. In response to the comment of the reviewer, we have updated some descriptions and limitations in the appropriate section of the revised manuscript.
-Revision : (SECTION: Experimental design) The experiment was designed to quantitatively acquire data related to the 11 different upper-extremity movements for both real-movement and MI tasks. The subjects conducted the experimental tasks using the same limbs. Decoding different tasks related to the same limb by using EEG signals could increase the number of possibilities of controlling the BCI system compared with typical somatosensory rhythm (SMR)-based BCIs which often only detected left-/right-hand and foot imagery [27].
(SECTION: Background and purpose) We believe that this work will con- tribute to the reliable neuro-rehabilitation of motor-disabled patients and a high-level BCI system for healthy users. Furthermore, to ensure the practicality of BCI technology, we look forward to investigating to robustly decode motor-related intention despite the different recording sessions and subject dependency (i.e., session-to-session problem [23, 28] and subject-independent problem [29]). However, at present, only few datasets exist to apply various types of real-world applications and to develop a robust neural decoding model. To overcome this difficulty, this work could contribute to the development of a practical BCI system based on deep learning techniques and multi-modalities by providing a large dataset.
(SECTION: Results and Discussion) Furthermore, we confirmed that the classification accuracy per task was slightly higher than the chance-level accuracy using the baseline method. Conversely, despite the difficult tasks involved in the experiments, the subjects successfully focused on the experiments so that we could obtain high-quality data. In the future, the users of this dataset can contribute to increasing the present classification accuracy using their novel methodology.

-Comment 33: Please explain further the suggested hybrid EEG/EMG BCI using the dataset. If a subject were capable of generating such large EMG by fully executing the arm/hand movements, why would he/she need the BCI?
-Response : Thank you for your critical comment. From the BCI viewpoint, the decoding of motor imagery and real movements from EEG signals was insufficient to apply to real-time applications. The BCI technology was developed for patients with motor disabilities to communicate with the external environment. Additionally, performing neuro-rehabilitation using BCI has induced significant brain plasticity, enabling the patients with motor-disabilities to communicate more actively than before. Therefore, BCI systems have been developed for improving system performance using an advanced novel approach and multimodal signals. As the reviewer mentioned, EMG

	signals, one of the multi-modal signals, were capable of generating a large amplitude. Some BCI investigators adopted a hybrid system using both the signals to support the system performance enhancement and to better rehabilitate some patients who could detect muscular activities. Furthermore, in the case of healthy people, the recent BCI technology is one of the AI-based advances for controlling external devices. In real-time systems, that use only EMG signals, muscular fatigue could be induced upon extended usage and also the users could not use body parts attached to signal detectors. To overcome these constraint limitations, a hybrid BCI system that uses multimodal signals has been developed for not only patients but also healthy people. In response to your suggestion, we have revised the manuscript regarding the usefulness of hybrid BCI using EEG and EMG as per the reference paper.<br>-Revision : (SECTION: Results and Discussion) Simultaneously, researchers can attempt to combine EEG and EMG signals using our dataset for developing hybrid BCI systems. In related studies, the hybrid approaches showed remarkable possibility to improve the decoding performances of real-movement and MI-based BCIs [34, 61]. Additionally, our dataset can be used for studies that analyze the correlations between EEG and EMG. In related studies, the relevance of EEG and EMG signals can be found through the connectivity analysis of the data acquired over a specific period. For example, a statistical analysis of activated EEG channels conducted during the activation of a particular EMG channel can determine the region of the brain, channel location, and frequency band directly related to the movement of the particular muscle [62].<br><br><br>-Comment 34: Please cite a few relevant literature that use similar ideas to the ones mentioned in the discussion section, i.e., the connectivity, the hybrid BCI, inter-session comparisons, transfer learning, etc. These are not novel ideas and should be properly cited.<br>-Response : Thank you for your important comment. In response to your comment, we have added the reference papers as example studies for discussing the connectivity, hybrid BCI, inter-session comparisons, and transfer learning.<br>-Revision : (SECTION: Results and Discussion) In related studies, the hybrid approaches showed remarkable possibility to improve the decoding performances of real-movement and MI-based BCIs [34, 61].<br>(SECTION: Results and Discussion) For example, a statistical analysis of activated EEG channels conducted during the activation of a particular EMG channel can determine the region of the brain, channel location, and frequency band directly related to the movement of the particular muscle [62].<br>(SECTION: Results and Discussion) Inter-session comparisons are also important topics in BCI experiments. Since the BCI systems are recalibrated at the beginning of each recording session, this procedure becomes time-consuming and thus may limit the adoption of BCI systems for long-term daily usage [63].<br>(SECTION: Results and Discussion) Different approaches are also available, and they include training the decoding model in a particular session and testing the model using data from independent sessions on the basis of the principle of transfer learning in BCI, as done in [64].<br><br><br>-Comment 35: What do the authors mean by this sentence in the last paragraph? “The variance of the averaged classification accuracy in session-to-session data was low.”<br>-Response : We agree with your critical remark that the submitted manuscript could cause confusion. For better comprehension, we have excluded the concerned sentence from the revised manuscript.<br><br><br>-Comment 36: Please provide a link to the repository.<br>-Response : Thank you for your valuable comment. In response to your comment, we will add the link of the dataset repository after the final decision regarding the acceptance of the manuscript. The current link was temporarily provided by a journal editor for the submission.
**Additional Information:**	
**Question**	**Response**

Powered by Editorial Manager® and ProduXion Manager® from Aries Systems Corporation

Are you submitting this manuscript to a special series or article collection?	No
**Experimental design and statistics**<br><br>Full details of the experimental design and statistical methods used should be given in the Methods section, as detailed in our Minimum Standards Reporting Checklist. Information essential to interpreting the data presented should be made available in the figure legends.<br><br>Have you included all the information requested in your manuscript?	Yes
**Resources**<br><br>A description of all resources used, including antibodies, cell lines, animals and software tools, with enough information to allow them to be uniquely identified, should be included in the Methods section. Authors are strongly encouraged to cite Research Resource Identifiers (RRIDs) for antibodies, model organisms and tools, where possible.<br><br>Have you included the information requested as detailed in our Minimum Standards Reporting Checklist?	Yes
**Availability of data and materials**<br><br>All datasets and code on which the conclusions of the paper rely must be either included in your submission or deposited in publicly available repositories (where available and ethically appropriate), referencing such data using a unique identifier in the references and in the "Availability of Data and Materials" section of your manuscript.<br><br>Have you have met the above requirement as detailed in our Minimum	Yes

Standards Reporting Checklist?	

Powered by Editorial Manager® and ProduXion Manager® from Aries Systems Corporation

Revised Manuscript
Click here to access/download;Manuscript;Revised Manuscript.pdf

OXFORD
(GIGA)^n SCIENCE

GigaScience, 2020, 1–12

doi: xx.xxxx/xxxx
Manuscript in Preparation
Data Note

DATA NOTE

Multimodal signal dataset for 11 intuitive movement tasks from single upper-extremity during multiple recording sessions

Ji-Hoon Jeong[1], Jeong-Hyun Cho[1], Kyung-Hwan Shim[1], Byoung-Hee Kwon[1], Byeong-Hoo Lee[1], Do-Yeun Lee[1], Dae-Hyeok Lee[1] and Seong-Whan Lee[1,2,*]

[1]Department of Brain and Cognitive Engineering, Korea University, 145 Anam-ro, Seongbuk-gu, Seoul 02841, South Korea and [2]Department of Artificial Intelligence, Korea University, 145 Anam-ro, Seongbuk-gu, Seoul 02841, South Korea

[*]**Correspondence address**. Seong-Whan Lee, Tel: +82-2-3290-3197; Fax: +82-2-3290-3583; E-mail: sw.lee@korea.ac.kr

Abstract

Background: Non-invasive brain-computer interfaces (BCIs) have been developed for realizing natural bi-directional interaction between users and external robotic systems. However, the communication between users and BCI systems through artificial matching is a critical issue. Recently, BCIs have been developed to adopt intuitive decoding which is the key to solving several problems such as a small number of classes and manually matching BCI commands with device control. Unfortunately, the advances in this area have been slow owing to the lack of large and uniform datasets. This study provides a large intuitive dataset for 11 different upper-extremity movement tasks obtained during multiple recording sessions. The dataset includes 60-channel electroencephalography (EEG), 7-channel electromyography (EMG), and 4-channel electrooculography (EOG) of 25 healthy subjects collected over 3-day sessions for a total of 82,500 trials across all the subjects. **Findings**: We validated our dataset via neuro-physiological analysis. We observed clear sensorimotor de-/activation and spatial distribution related to real-movement and motor imagery (MI), respectively. Furthermore, we demonstrated the consistency of the dataset by evaluating the classification performance of each session using a baseline machine learning method. **Conclusions**: The dataset includes the data of multiple recording sessions, various classes within the single upper-extremity, and multimodal signals. This work can be used to *i*) compare the brain activities associated with real-movement and imagination, *ii*) improve the decoding performance, and *iii*) analyze the differences among recording sessions. Hence, this study, as a data note, has focused on collecting data required for further advances in the BCI technology.

Key words: brain-computer interface (BCI); multimodal signals; intuitive upper-extremity movements; multiple sessions

Data Description

Background and purpose

The brain-computer interface (BCI) technology allows users to communicate with external devices including a speller [1], wheelchair [2], robotic arm [3–5] and robotic exoskeleton [6, 7]. A noninvasive BCI commonly employs electroencephalography (EEG) signals to decode user intentions [8–11] because the EEG-based BCI system offers lower risk, lower cost, and more convenient than other non-invasive BCI paradigms (e.g., functional near-infrared spectroscopy (fNIRS) [12]). EEG-based

Compiled on: August 13, 2020.
Draft manuscript prepared by the author.

BCIs have been developed using various paradigms including motor imagery (MI) [13–15], steady-state visual evoked potential (SSVEP) [16, 17], event-related potential (ERP) [18], and movement-related cortical potential (MRCP) [19, 20]. Over the past decades, the information regarding the EEG datasets of these general paradigms have been published through competitions, cooperation projects, and open-access articles [21–25]. Some research groups have developed advanced machine learning algorithms and deep learning architectures for improving the BCI performance using these datasets.

The recent advances in BCI systems have been focused on topics ranging from intuitive EEG decoding to directly matching the interaction between user intention and device feedback for real-world environments [20, 26]. For example, to control a neuro-prosthetic arm using typical BCI paradigms, we temporarily matched BCI commands with robotic arm motions (e.g., MI for both hands = grasping motion of the robotic hand). However, this unintended artificial matching suffers from several constraint limitations, such as the small number of restrictive classes for communicating with devices and inflexible user training due to unintuitive commands in real-world scenarios [7, 13, 27]. For example, if a robotic hand performs the grasping motion, the users should also imagine the hand grasping motion the same as the robotic hand motion to enhance the natural decoding experience [27].

In this work, we collected data on intuitive upper-extremity movements from 25 subjects. To collect high-quality signal data, the experiments were conducted on healthy subjects, who had maintained good physical conditions by, for instance, limiting their alcohol intake and getting sufficient sleep. We focused on various upper-extremity motions because they are the most extensible and available movements among all the body movements. Accordingly, we selected the upper extremities for decoding intuitive movements and then collected data based on the movement-based multimodal signals. The subjects were asked to perform 11 different movement tasks: arm-reaching along six directions, hand-grasping of three objects, and wrist-twisting with two different motions. The corresponding 11 classes were designed for each segmented motion related to the arm, hand, and wrist, rather than for continuous limb movements. Therefore, the users of our dataset could either conduct respective analyses for individual classes or attempt decoding the complex upper-extremity movements by combining data from different classes. For researchers focused on more advanced and analytical approaches using multimodal signals, the dataset comprised not only EEG data but also electromyography (EMG) and electrooculography (EOG) data. These data were synchronously collected in the same experimental environment, while ensuring no unintentional interference between them. The data acquired using a 60-channel EEG, 7-channel EMG, and 4-channel EOG were simultaneously recorded during the experiment. EEG sensors were placed according to international specifications to collect signals from all the regions of the scalp. Additionally, EMG sensors were attached to carefully selected locations on the right arm to reflect the most relevant muscle-activity information associated with the corresponding upper limb movement. We also recorded the EOG signals using four channels independent of the EEG channels to capture detailed eye movements, which were mainly used for artifact removal. The subjects performed real upper-extremity movements and MI associated with the 11 aforementioned motions. Additionally, each subject participated in three recording sessions at one-week intervals and followed the same experimental protocols. To acquire a large-amount of high-quality data, we prioritized the physical and mental conditions of the subjects as a priority during the experiments. Eventually, the multimodal signal dataset became sufficiently large for the BCI experiment because it now included data acquired from 82,500 trials performed for all the subjects (i.e., 3,300 trials were collected per subject).

To the best of our knowledge, the present dataset descriptor is the first large public dataset for intuitive BCI paradigms to include multimodal signals such as EEG, EMG, and EOG signals. This study might contribute to the realization of reliable neuro-rehabilitation of patients with motor disabilities and a high-level BCI system for healthy users. Furthermore, to ensure the practicality of the BCI technology, we intend to investigate how to robustly decode motor-related intentions despite different recording sessions and subject dependency (i.e., the session-to-session problem [23, 28] and subject-independent problem [29]). However, presently, only few datasets exist to be applied to various types of real-world applications and to develop a robust neural decoding model. To overcome this challenge, this study could contribute to the development of a practical BCI system based on deep learning techniques and multi-modalities by providing a large dataset.

Experimental design

Subjects

Twenty-five subjects (all-right handed, S1-S25, aged 24-32 years, 15 males and 10 females) who were naïve BCI users participated in the experiments. They were healthy individuals with no known neurophysiological anomalies or musculoskeletal disorders. Before the experiments, they were informed about the experimental protocols, paradigms, and purpose. After ensuring that they had understood the information, they provided their written consent according to the Declaration of Helsinki. The subjects signed a form that agreed to the anonymous public release of their data. We checked their physical and mental states for comparing the influence of the BCI performance according to individual state. Additionally, each subject was required to be in normal health, get sufficient sleep (approximately 8 h), and avoid alcohol, caffeinated drinks, and strenuous physical activity before the experiments. All the experimental protocols and environments were reviewed and approved by the Institutional Review Board (IRB) at Korea University (1040548-KU-IRB-17-181-A-2).

Environment

During the experiments, each subject was comfortably seated in a chair with armrests facing the front of an LCD monitor, approximately 80 ($\pm$5)cm away from each other [30]. An EEG cap (Fig. 1) with 60 channels (actiCap, BrainProduct GmbH, Germany) was placed on the head of each subject. Surface EMG and EOG electrodes were attached to the pre-assigned locations on the right arm and around the eyes of each subject, respectively. The subjects were then asked to perform the movements with relaxed muscles and minimum eye and body movements during the data recording.

The duration of the experiment was approximately 6-7 h a day. Our experiment comprised multiple recording sessions (three days) to consider inter-session and inter-subject variabilities. Compared with typical BCI experiments, our experiments required a longer recording time. To maintain the physical and mental conditions of the subjects and thus ensure high signal quality, the subjects took sufficient breaks between each task. During the breaks, we first confirmed the physical and mental conditions of the subjects through self-report. If they reported of any inconvenient position or unstable conditions, we either adjusted the experimental environment according to their requests or halted the experiment. In the case the experiment was halted, the subjects could ask to conduct the experiment next time or not perform the experiment altogether. However, if the conditions of the subjects were good to con-

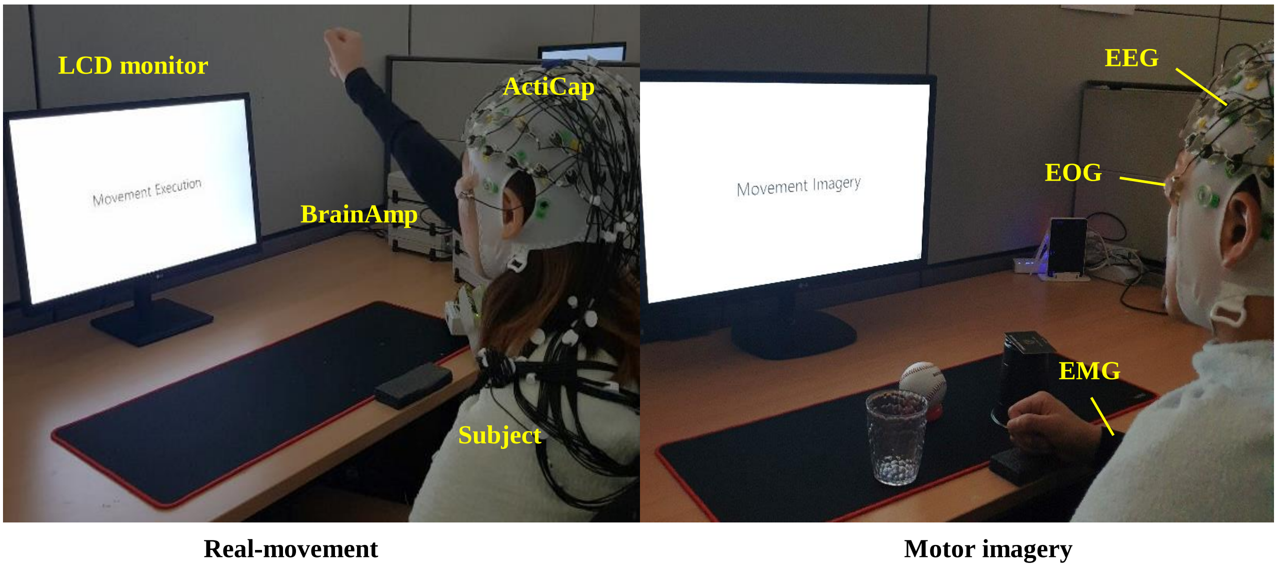

Figure 1. Experimental environments for acquiring multimodal signals related to the intuitive movement tasks. The subjects were asked to perform real-movement (e.g., arm-reaching) and MI tasks (e.g., hand-grasping).

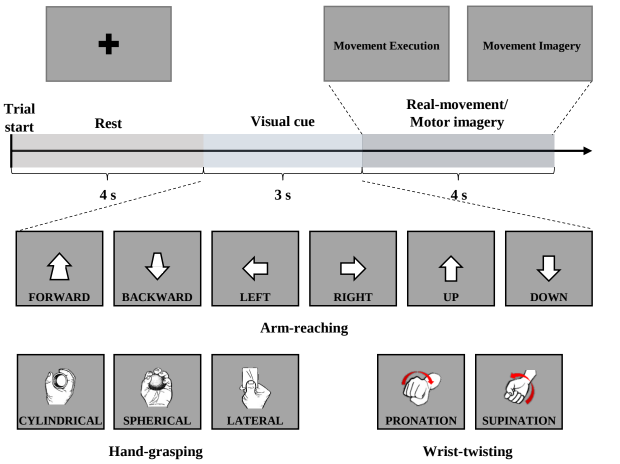

Figure 2. Experimental paradigm in a single-trial and the representation of visual cues according to each task.

duct the experiment, we checked the impedances of the EEG, EMG, and EOG electrodes and injected electrolyte gel into them to maintain the impedance values below 15 kΩ. Therefore, we attempted to obtain clear signals excluding the spontaneous noise due to muscle and mental fatigue during the recording.

Experimental paradigm

The experiment was designed to quantitatively acquire data related to the 11 different upper-extremity movements for both real-movement and MI tasks. The subjects conducted the experimental tasks using the same limbs. Decoding different tasks related to the same limb by using EEG signals could increase the number of possibilities of controlling the BCI system compared with typical somatosensory rhythm (SMR)-based BCIs which often only detected left-/right-hand and foot imagery [27]. The experimental tasks comprised three main upper-extremity motions: arm-reaching, hand-grasping, and wrist-twisting. When the experiment began, visual instructions were provided on the monitor by displaying a black cross sign and a gray background. The subjects stared at the visual

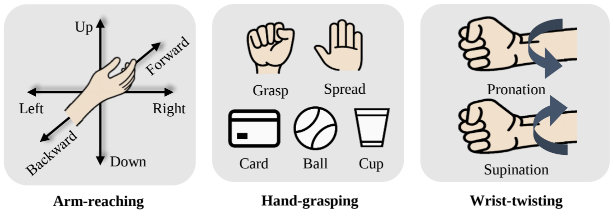

Figure 3. Experimental tasks of 11 intuitive upper-extremity movements related to arm-reaching, hand-grasping, and wrist-twisting, respectively.

instructions for 4 s while resting. After resting, a visual cue was displayed on the monitor with a text sign for 3 s, following which the subjects began preparing to perform the real-movement or MI tasks according to the visual cue (see Fig. 2). Upon changing the visual cue to a text sign as "Movement execution" and "Movement imagery", the subjects performed the corresponding tasks during 4 s. During the real-movement tasks, the subjects were asked to focus on the sensations involved with each motion and to remember those sensations for the MI tasks.

Arm-reaching along six directions: The subjects were asked to perform multi-direction arm-reaching tasks directed from the center of their bodies to the outward direction. They performed the tasks along six different directions in the 3D space: forward, backward, left, right, up, and down, as depicted in Fig. 3. In the real-movement tasks, the subjects extended their arms along one of the directions. The arm-reaching paradigm required 50 trials along each direction so that data could be collected for a total of 300 trials. However, in the MI tasks, the subjects only imagined performing an arm-reaching task; the number of trials in the MI paradigm was the same as in the real-movement paradigm.

Hand-grasping three objects: The subjects were asked to grasp three objects of daily use via the corresponding grasping motions. They performed the three designated grasp motions by holding the objects, namely, card, ball, and cup, corresponded to cylindrical, spherical, and lateral grasp, respectively (see Fig. 3). In the real-movement tasks, we asked the subjects to use their right hands to grasp a randomly selected object and hold it using its corresponding grasping mo-

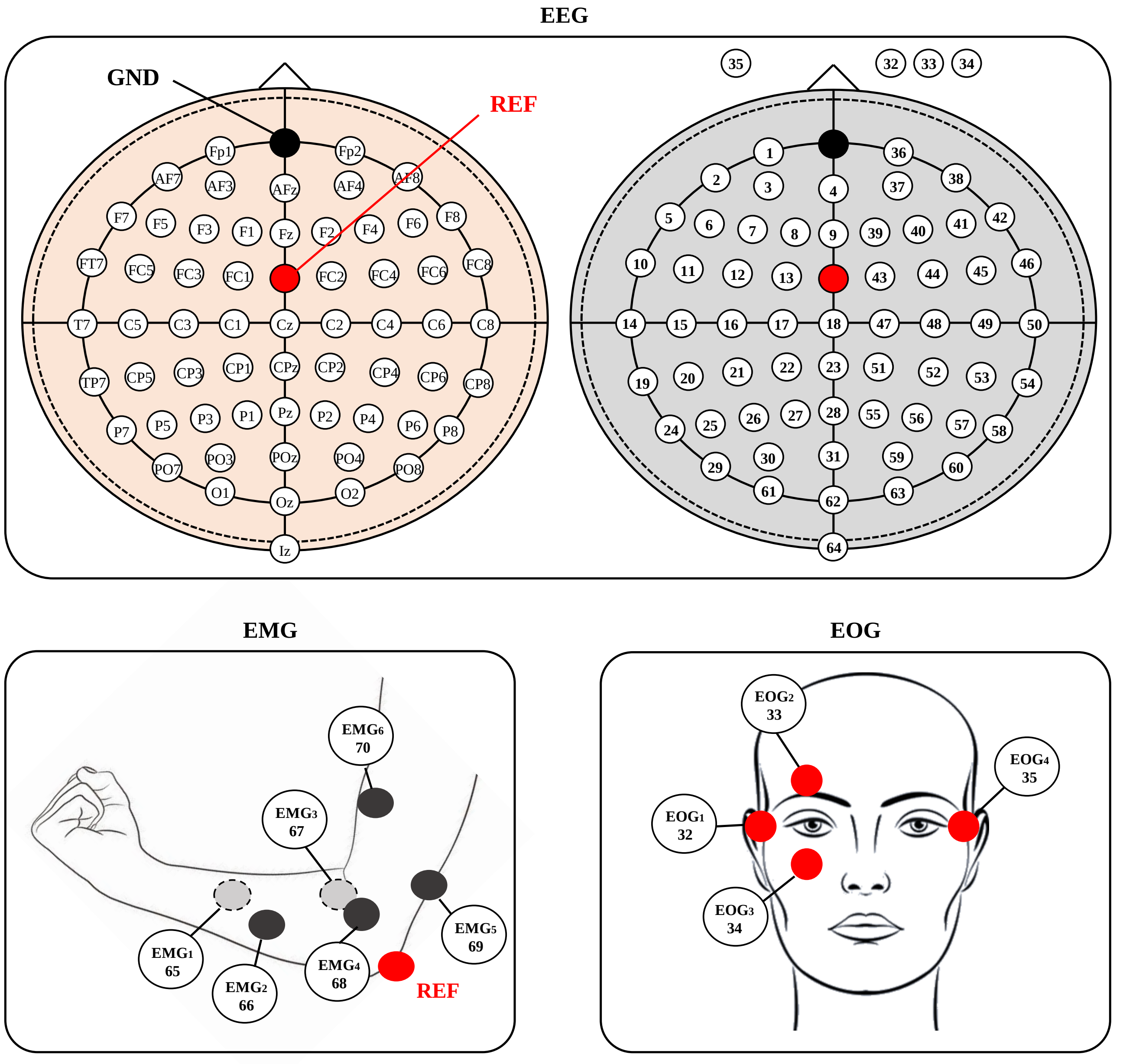

Figure 4. Data configuration for 60 EEG, 7 EMG, and 4 EOG channels. Specifically, 4 EOG channels were numbered as 32–35 out of the 64-channel actiCAP montage.

tion. Eventually, we acquired data on 50 trials for each grasp, and hence, we collected 150 trials per subject. In the MI tasks, the subjects performed only one of the three grasping motions per trial, randomly. The number of trials in the MI paradigm was the same as that in the real-movement paradigm.

Wrist-twisting with two different motions: For the wrist-twisting tasks, the subjects rotated their wrists to the left (pronation) and right (supination), as depicted in Fig. 3. During real-movement task, each subject maintained his/her right hand in a neutral position with the elbow comfortably placed on the desk. Notably, wrist pronation and supination are complex actions used to decode user intentions from brain signals. Additionally, these movements are intuitive motions for realizing neuro-rehabilitation and prosthetic control [31]. We collected data for 50 trials per motion (i.e., total 100 trials) per day, and the visual cues were randomly displayed.

Additionally, the subjects were asked to participate in three recording sessions with a one-week interval between each session. The experimental environment and protocols were the same for all the three sessions. Consequently, we collected data from 3,300 trials (1,800 trials for arm-reaching, 900 for hand-grasping, and 600 for wrist-twisting) in all classes per subject, both for real-movement and MI paradigms.

Data Records

We simultaneously collected three different kinds of physiological signals, namely, EEG, EMG, and EOG signals for 11 different upper-extremity movements (see Fig. 3). During the experiment, the signals were acquired using the same digital amplifier and types of electrodes. Therefore, the raw signals were stored together in one data file according to each subject. To obtain high-quality signals, the impedances of all the channels were maintained to be below 15 kΩ. After applying the conductive gel to the electrodes, we validated the accuracy of the EEG and EOG signals by asking the subjects to blink and close their eyes. The eye-blinking task was used to identify strong spikes

Table 1. Data description for *Raw data* folder and *Converted data* folder

Raw data		Converted data (e.g., session1_sub1_multigrasp_MI.mat)		
Name	Description	Name		Description
session1_sub1_(*Task*)_(realMove/MI).eeg	Raw signals (session I)	.dat	.x	Pre-processed data
session1_sub1_(*Task*)_(realMove/MI).vhdr	Data header information (session I)		.fs	Sampling frequency
session1_sub1_(*Task*)_(realMove/MI).vhdr	Marker information for (session I)		.file	File name
			.clab	Channel information
		.mnt	.x	X coordinates for channel position
session2_sub1_(*Task*)_(realMove/MI).eeg	Raw signals (session II)		.y	Y coordinates for channel position
session2_sub1_(*Task*)_(realMove/MI).vhdr	Data header information (session II)		.pos_3d	3D coordinates for channel position
session2_sub1_(*Task*)_(realMove/MI).vhdr	Marker information for (session II)		.clab	Channel information
		.mrk	.pos	Trigger marking time
			.toe	Trigger number
session3_sub1_(*Task*)_(realMove/MI).eeg	Raw signals (session III)		.fs	Sampling frequency
session3_sub1_(*Task*)_(realMove/MI).vhdr	Data header information (session III)		.y	Class lebels
session3_sub1_(*Task*)_(realMove/MI).vmrk	Marker information for (session III)		.className	Class name
			.mics	Experiment start and end information

in the frontal EEG channels (channel Fp1 and Fp2) and 4 EOG channels. The eye-closing task was used to confirm the alpha oscillations in the occipital channels (e.g., O1, O2, and Oz). We also asked the subjects to perform a simple hand-grasping motion to confirm the strong spikes in the EMG signals.

EEG signals

The EEG data were recorded in conjunction with an EEG signal amplifier (BrainAmp, BrainProduct GmbH, Germany), sampled at 2,500 Hz. Additionally, we applied a 60 Hz with a notch filter to reduce the effect of external electrical noises (e.g., DC noise due to power supply, scan rate of the monitor display, and frequency of the fluorescent lamp) in raw signals [21, 32, 33]. The raw data were recorded using BrainVision (BrainProduct GmbH, Germany) with MATLAB 2019a (The MathWorks Inc., USA). Furthermore, a toal of 60 EEG electrodes were selected by following a 10-20 international configuration (Fp1-2, AF5-6, AF7-8, AFz, F1-8, Fz, FT7-8, FC1-6, T7-8, C1-6, Cz, TP7-8, CP1-6, CPz, P1-8, Pz, PO3-4, PO7-8, POz, O1-2, Oz, and Iz). Ground and reference channels were placed on the Fpz and FCz, respectively (see Fig. 4). The impedances of all the electrodes between the sensors and scalp skin were maintained to be below 15 kΩ. During the break time, conductive gel was injected into the electrodes using a syringe with a blunt needle.

EMG signals

The EMG signals were recorded using 7 Ag/AgCl electrodes from the digital amplifier, the same equipment used to record the EEG signals. We simultaneously acquired the EMG and EEG signals using the same amplifer [34]. The signals were captured at a sampling rate of 2,500 Hz with a 60 Hz notch filter same as the setting used to record the EEG signals. The EMG data were recorded from six related muscles for right arm movement: extensor carpi ulnaris, extensor digitorum, flexor carpi radialis, flexor carpi ulnaris, biceps brachii, and triceps brachii (see Fig. 4) [35]. The ground and reference were recorded in Fpz and FCz, respectively, which are the same as the EEG and EOG signals. The last electrode was placed on the elbow of the right arm which is a non-muscle movement area, as an alternative reference signal [36].The purpose of recording EMG signals was to detect muscle activities when the subjects performed the designated tasks. The signals could prove that the subjects performed MI tasks without muscle movement. Simultaneously, the electrodes were placed so as to record sufficient number of signals from various arm and hand movements (i.e., six arm-reaching, three hand-grasping, and two wrist-twisting actions).

EOG signals

The EOG signals were recorded using 4 channels while following the same protocol. Subsequently, the FT9, FT10, TP9, and TP10 electrodes were moved to the region around the eyes to function as EOG channels to eliminate the artifacts due to ocular activities. One of these channels was moved to the region around the left eye and the others to the region around the right eye (see Fig. 4). The electrodes EOG_1 and EOG_4 were used to record horizontal eye movements, while EOG_2 and EOG_3 were used to record vertical movements [37]. A medical tape was used to hold the sensors around the eyes and maintain the impedances of all the electrodes to be below 15kΩ.

Data format and structure

Readers can access our codes and datasets through the GigaDB repository. The "*Read me*".pdf file, which overviews of data description and code execution, is included in the repository. Several useful scripts, including *Data_analysis*.m and *Visualization*.m files, are in the "SampleCode" folder. We recommend the BBCI (http://www.bci.de) toolbox [38] in the "Reference toolbox" folder and the "Signal Processing" toolbox in the MATLAB software, for data processing using our custom code. Please directly contact the authors for more information on the code script.

Each dataset (raw signals, converted data, and scripts) is also publicly available via the GigaDB repository. The raw signals and converted data are contained in the corresponding folders, namely, "Raw data" and "Converted data", respectively. Table 1 summarizes the data description for both the folders. The indicated *Task* includes the names of arm-reaching, multigrasp, and twisting. We provide the folders, namely,: "Raw data" and "Converted data" which comprise *.eeg*, *.vmrk*, *.vhdr*, and *.mat* files for each subject. The *.eeg* file includes the raw EEG, EOG, and EMG signals data because of the simultaneous data acquisition performed using the same amplifier. Moreover, the *.vmrk* file provides the marked trigger information (e.g., trigger number, marked time, and file name) and the *.vhdr* file includes the number of channels, sampling rate, channel position, and electrode impedances. The *.mat* file includes pre-processed EEG, EOG, and EMG data, channels, class information, scalp montage, and sample frequency. Additionally, to enable easy data access for the users, we pro-

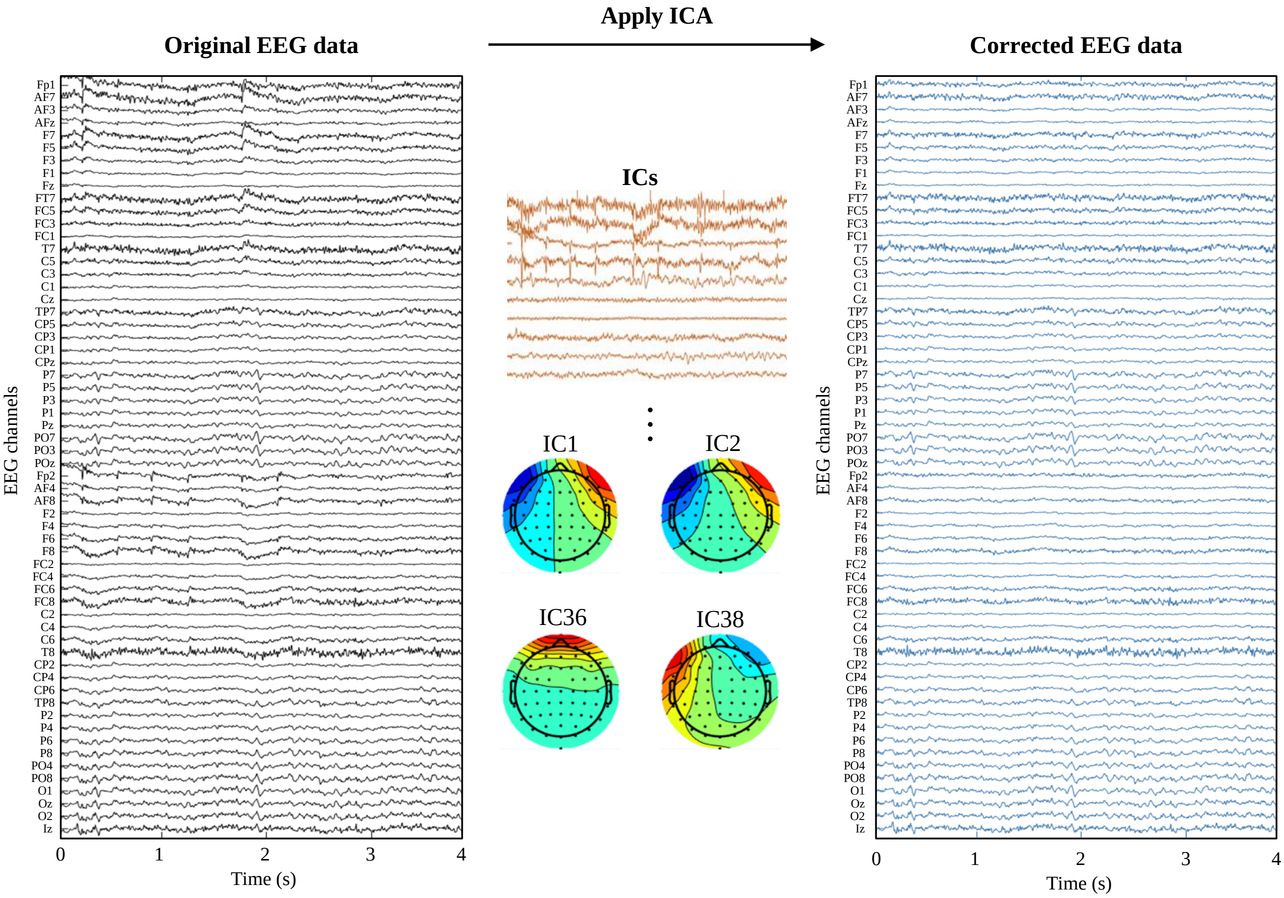

Figure 5. Infomax ICA based on EOG data was applied to eliminate the eye-movement-induced noise from the original EEG data. The right side shows the corrected EEG data wherein noise has been eliminated by the application of the infomax ICA to the original EEG data.

vided the dataset after converting the .eeg file to .mat file in the "Converted data" folder. The converted .mat file includes some information such as trigger mark information, channel configuration, epoch segmentation, and etc.

Data_Analysis.m provides the basic data-processing script, which includes data loading, signal pre-processing, artifact rejection, feature extraction, classification, and performance evaluation. All users can download and unzip the '*SampleData*'.zip file contained in the "SampleData" folder before executing each code.

Visualization.m enables the visualization of raw signals, scalp distribution, and event-related spectral perturbation (ERSP) using EEGLAB [39]. The raw signals were visualized as channels through the time representation for the representative subject named sub8. The scalp plot can be visualized to choose a specific channel and time epoch for a selected subject. The ERSP plot requires the installation of the EEGLAB toolbox. After installing the EEGLAB toolbox, users can load the *.vhdr* file to EEGLAB and visualize the ERSP pattern that follows the description.

Data Validation

Methods

The technical signal validation was conducted using a BBCI toolbox [38] in the MATLAB 2019a environment. The initial settings of the recording program for converting an analog signal into a digital one were slightly different for each signal because of the scale of the signal amplitude. For each of the EEG, EMG, and EOG signals, triggers were marked to indicate the experimental state.

Initially, for data pre-processing, we applied a zero-phase 4th Butterworth filter for band-pass signal filtering in the EEG signals. The data were filtered between 8 and 30 Hz (mu- and beta-bands, respectively) known as within motor-related frequency range and it could also include the spectral range for SMR observation (i.e., [13-15] Hz) [40, 41]. For artifact rejection, the apparent eye-blinking contamination in the EEG signal was removed via independent component analysis (ICA) [42]. To obtain corrected EEG data, we removed the contamination factors using the infomax ICA [43], which is used to decompose brain signals into statistically independent components (ICs). From various types of ICA methods, we adopted the ICA with the infomax algorithm as it could robustly remove artifacts, such as eye and head movement artifacts from the EEG data robustly [44]. The EEG data were transformed by the ICA mixing matrix. The contaminated ICs with patterns similar to the EOG channels (i.e., horizontal and vertical eye movements) were removed. Subsequently, the remaining ICs were projected back into the scalp channel space to be reconstructed as the corrected EEG data (see Fig. 5). Before feature extraction, we segmented a time interval of -0.5 to 4 s for performing EEG classification and also selected a baseline period as -0.5 to 0 s [25].

For evaluating the classification performances using EEG signals, we adopted the common spatial pattern (CSP) algorithm as a feature extraction method and a regularized linear discriminant analysis (RLDA) method as the classification method. CSP feature extraction method and RLDA classifier are generally used as baseline algorithms for decoding EEG-based

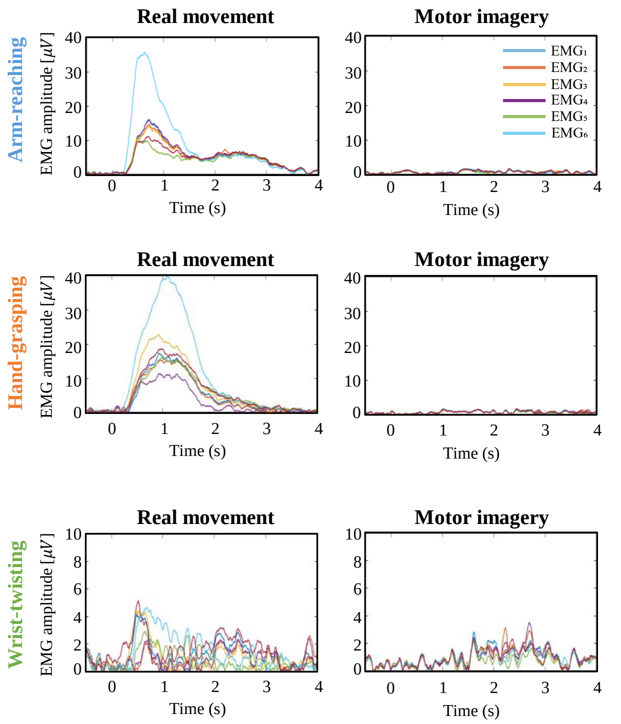

Figure 6. Example representation of EMG activation according to each 7-channel EMG. From the top, the plot represents the activities of the EMG signals as the representative subject sub4 performs arm-reaching, hand-grasping, and wrist-twisting tasks, respectively.

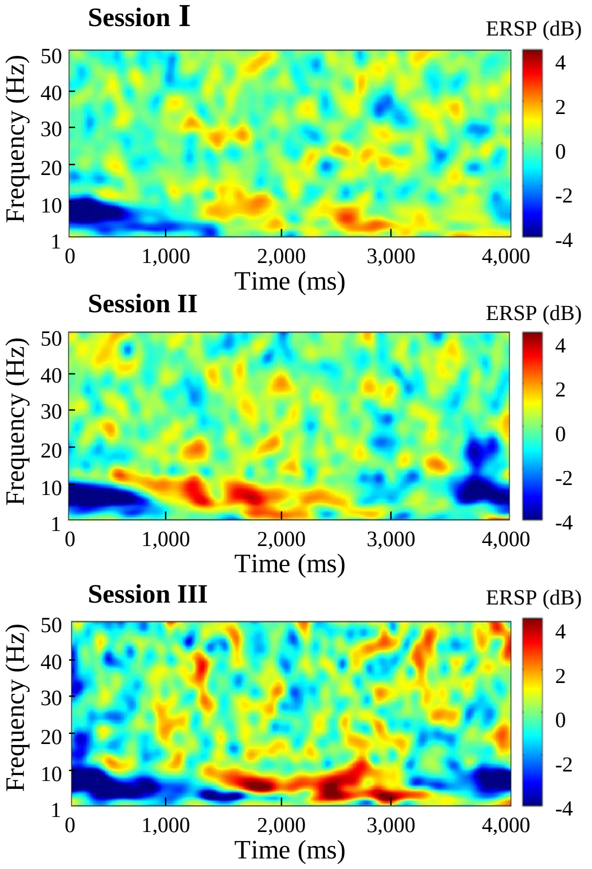

Figure 7. EEG data validation via spectral representation. The event-related desynchronization/synchronization (ERD/ERS) representation of channel C3 during multiple recording sessions.

MI and motor execution in the field of BCI [26, 45, 46]. Especially, CSP, one of the EEG feature extraction methods, is proved to be robust in spatial feature extraction for decoding movement-related tasks and MI. CSP trained for finding the optimal spatial filter that maximizes inter-class variation and minimizes intra-class variation. We calculated a transformation matrix using CSP consisting of the logarithmic variances of the first and last three columns were used as a feature. We trained the RLDA classifier by adding a regularization term to the covariance matrix using the optimal shrinkage parameters [47, 48]. Essentially, shrinkage performs regularization to improve the estimation of covariance matrices where the training samples are fewer than features. Therefore, during the training period, the optimal shrinkage parameter was automatically estimated with the maximum covariance between classes. In the classification procedure, we classified the multi-class according to each different experimental task, separately, such as arm-reaching along six directions (6-class), hand-grasping of three objects (3-class), and wrist-twisting with two different motions (2-class), as depicted in Fig. 3. We applied 10×10-fold cross-validation for fair performance measurement so that we partitioned the data samples as equal sizes into 10 subsets. One of the subsets was selected as the test dataset and the remaining others as the training datasets. The cross-validation process was conducted 10 times with each of the 10 subsets used once as the test dataset to avoid variability problems in performance evaluation. The evaluation was estimated using all recorded classes simultaneously over all the recording sessions. Table 2 presents the averaged evaluation results obtained by estimating the classification performance of each arm-reaching (6-class), hand-grasping (3-class), and wrist-twisting (2-class) task.

Furthermore, we checked the EMG activation to verify whether the upper-extremity movement or not according to the tasks. In this work, the EMG signals were pre-processed from 10–500 Hz with a Butterworth 5th zero-phase bandpass filter [34, 49]. We segmented a time interval of -0.5 to -4 s for EMG data analysis. We selected the interval of -0.5 to 0 s for as the baseline period. Subsequently, the data were rectified using the absolute values, following which we calculated the moving average of EMG amplitudes with a 100 ms interval. The EMG signals could show how well the subjects had followed the experimental protocol for each task. For example, if the subject were asked to perform the MI task, the EMG activation should not show a peak shape because of the static state at that time, as depicted in Fig. 6. EMG signal patterns contains important information regarding muscle activation and noise. For example, the noise due to heart beat and other unrelated movements reduces the quality of EMG signals. While the subjects performed the wrist-twisting task, the amplitude scale was reduced as compared with other work, so that noise information could be confirmed to be displayed. Meanwhile, in the real-movement tasks, the EMG signals could show the activation while the subjects were performing the upper-extremity movement task. Notably, the signal amplitude of channel EMG_6 is higher than those of the other channels. Since biceps are relatively bigger than other muscles, a peak phenomenon occurred [50]. Particularly, biceps are used the most in bending the arm. The EMG signals featured a large signal amplitude, as more muscle activity increases the signal amplitude. Consequently, the EMG signal amplitude was large for the arm-reaching and hand-grasping tasks, but small for the wrist-twisting task [51].

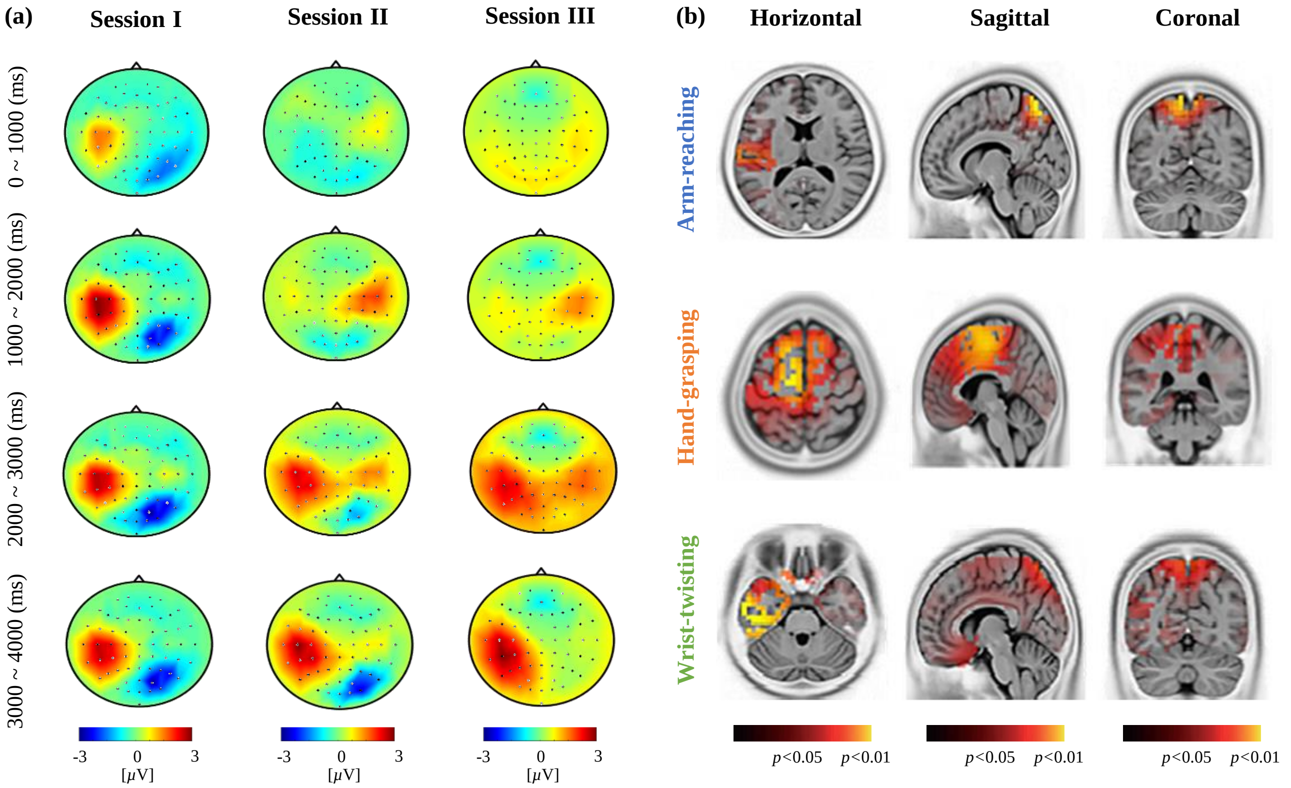

Figure 8. Data validation performed using the spatial information with respect to scalp activation. (a) Scalp topography of the wrist pronation task for a representative subject named sub2. (b) Source imaging analysis performed via sLORETA using by a statistically significant difference in the MI tasks for a representative subject named sub13 (red: $p<0.05$, yellow: $p<0.01$).

Results and Discussion

We verified the data through the EEG-based classification results, the EMG signal quality, and spectral and spatial EEG presentations. First, we verified the data on the basis of EMG signal quality, as shown in Fig. 6. Using the EMG signals recorded while the subjects performed each arm-reaching, hand-grasping, and wrist-twisting experiment, we could confirm the quality of the data obtained. Furthermore, dynamic EMG signals were observed during the real-movement sessions; additionally insignificant changes were observed in the EMG signals close to the rest state in the MI sessions. The EMG signals, as particularly, proved that we had collected the data appropriately, as they were not activated throughout the entire duration of the actual motion session, and appeared strongly for the actual movements of the subjects right after approximately 0.5 seconds from the onset. Additionally, the non-activated EMG signals during the MI tasks clearly indicate that the movement artifacts of the corresponding EEG signal were minimized.

We also analyzed the EEG signals in the spectral and spatial domains to confirm the data quality. Fig. 7 shows the examples of spectral energy information in the EEG data for a representative subject during multiple recording sessions. The ERSP plot illustrates spectral variability according to the time epoch in a certain channel (C3). Generally, the ERSP plot showed ERD/ERS patterns, which reflect sensorimotor activation and deactivation, respectively. The ERD patterns can be seen during motor preparation, execution, and imagery as correlations in an activated cortical region. ERS can be observed after the imagery or execution of movement over the same region [52, 53]. In Fig. 7, ERD/ERS patterns appeared during all the imagery phases (0 to 4 s), showing the same cortical activation on the mu-band across all the recording sessions. Additionally, we conducted a statistical analysis to confirm whether the subjects consistently performed MI via their single upper-extremities as instructed at the beginning. We selected typical EEG channels on the motor cortex corresponding to right-hand (channel C3), left-hand (channel C4), and foot (channel Cz) imageries [54, 55]. The mean ERD/ERS values for channel C3 contain significant differences compared with other channels (Cz and C4), as confirmed through the paired t-test. All the p-values between the channels were below 0.05 except for a few subjects.

Fig. 8(a) depicts the representative spatial distribution obtained using grand-average signal amplitude responses per time period [56, 57]. For subject sub2, the data for wrist pronation is presented in Fig. 8(a). We used all of EEG channels and adopted signal processing (similar to that in the preprocessing steps), such as band-pass filtering and epoch segmentation, and we also employed a baseline period. We applied the moving average of EEG amplitudes with a 200 ms interval. The topographic maps of the mean amplitudes were visualized in four temporal intervals for all the recording sessions. Therefore, the left-hemisphere of the contralateral sensorimotor region was activated while performing tasks in all the recording sessions. Hence, over time, the mean amplitude in the contralateral sensorimotor region of the left hemisphere significantly increased clearly as the subjects performed MI. Moreover, it was clearly observed that the contralateral sensorimotor regions of the most subjects were activated by MI in the 3 to 4 s. Therefore, we confirmed 0 to 2 s as the preparation period and that the contralateral sensorimotor region was appropriately activated after the first 2 s in the recording phases.

For a more sophisticated analysis, we used a source imaging technique to identify and compare activated regions in the brain with each other while a subject was performing each task. We used a standardized low-resolution electromagnetic tomog-

Table 2. Classification accuracy for each task across all the subjects during multiple recording sessions. The evaluation was estimated from each upper-extremity motions such as arm-reaching (6-class), hand grasping (3-class), wrist-twisting (2-class) in real-movement and MI.

Subjects	Session I						Session II						Session III					
	Real-movement			Motor imagery			Real-movement			Motor imagery			Real-movement			Motor imagery		
	Reach	Grasp	Twist	Reach	Grasp	Twist	Reach	Grasp	Twist	Reach	Grasp	Twist	Reach	Grasp	Twist	Reach	Grasp	Twist
sub1	0.21 (±0.01)	0.40 (±0.02)	0.61 (±0.03)	0.21 (±0.02)	0.42 (±0.04)	0.60 (±0.04)	0.21 (±0.02)	0.41 (±0.03)	0.56 (±0.03)	0.21 (±0.01)	0.42 (±0.04)	0.58 (±0.03)	0.24 (±0.01)	0.43 (±0.03)	0.58 (±0.03)	0.21 (±0.02)	0.40 (±0.03)	0.57 (±0.03)
sub2	0.24 (±0.01)	0.41 (±0.03)	0.56 (±0.04)	0.22 (±0.01)	0.40 (±0.02)	0.63 (±0.02)	0.23 (±0.02)	0.41 (±0.03)	0.63 (±0.03)	0.23 (±0.01)	0.40 (±0.02)	0.56 (±0.04)	0.20 (±0.03)	0.41 (±0.02)	0.66 (±0.02)	0.21 (±0.02)	0.40 (±0.02)	0.60 (±0.04)
sub3	0.18 (±0.02)	0.42 (±0.02)	0.59 (±0.04)	0.20 (±0.01)	0.39 (±0.04)	0.61 (±0.02)	0.19 (±0.02)	0.40 (±0.03)	0.59 (±0.03)	0.20 (±0.02)	0.38 (±0.03)	0.63 (±0.05)	0.20 (±0.01)	0.41 (±0.03)	0.56 (±0.04)	0.19 (±0.02)	0.36 (±0.02)	0.55 (±0.05)
sub4	0.17 (±0.02)	0.40 (±0.05)	0.64 (±0.03)	0.21 (±0.02)	0.39 (±0.03)	0.60 (±0.05)	0.28 (±0.02)	0.39 (±0.01)	0.59 (±0.03)	0.18 (±0.02)	0.38 (±0.03)	0.59 (±0.03)	0.21 (±0.01)	0.41 (±0.02)	0.57 (±0.05)	0.20 (±0.02)	0.40 (±0.02)	0.54 (±0.03)
sub5	0.21 (±0.01)	0.39 (±0.03)	0.59 (±0.02)	0.20 (±0.01)	0.43 (±0.03)	0.55 (±0.03)	0.20 (±0.02)	0.41 (±0.03)	0.54 (±0.04)	0.18 (±0.01)	0.35 (±0.03)	0.56 (±0.02)	0.19 (±0.02)	0.41 (±0.02)	0.55 (±0.06)	0.22 (±0.01)	0.35 (±0.03)	0.58 (±0.02)
sub6	0.19 (±0.02)	0.41 (±0.03)	0.60 (±0.04)	0.19 (±0.02)	0.46 (±0.04)	0.55 (±0.04)	0.22 (±0.02)	0.43 (±0.04)	0.60 (±0.03)	0.24 (±0.01)	0.44 (±0.03)	0.60 (±0.03)	0.24 (±0.02)	0.47 (±0.03)	0.62 (±0.05)	0.21 (±0.01)	0.46 (±0.02)	0.54 (±0.03)
sub7	0.22 (±0.01)	0.38 (±0.03)	0.61 (±0.03)	0.24 (±0.02)	0.53 (±0.03)	0.63 (±0.03)	0.21 (±0.02)	0.53 (±0.03)	0.60 (±0.02)	0.27 (±0.02)	0.60 (±0.02)	0.63 (±0.03)	0.21 (±0.02)	0.65 (±0.03)	0.57 (±0.04)	0.34 (±0.02)	0.53 (±0.03)	0.59 (±0.03)
sub8	0.20 (±0.01)	0.50 (±0.03)	0.59 (±0.03)	0.21 (±0.02)	0.54 (±0.03)	0.65 (±0.04)	0.22 (±0.02)	0.66 (±0.03)	0.53 (±0.03)	0.19 (±0.02)	0.79 (±0.03)	0.63 (±0.04)	0.22 (±0.02)	0.65 (±0.02)	0.61 (±0.05)	0.19 (±0.01)	0.75 (±0.02)	0.59 (±0.04)
sub9	0.20 (±0.01)	0.38 (±0.04)	0.57 (±0.02)	0.25 (±0.01)	0.35 (±0.03)	0.56 (±0.02)	0.21 (±0.02)	0.38 (±0.03)	0.62 (±0.05)	0.21 (±0.02)	0.39 (±0.03)	0.60 (±0.04)	0.20 (±0.02)	0.41 (±0.03)	0.58 (±0.04)	0.19 (±0.01)	0.39 (±0.05)	0.59 (±0.05)
sub10	0.20 (±0.02)	0.43 (±0.02)	0.58 (±0.05)	0.21 (±0.02)	0.41 (±0.02)	0.55 (±0.02)	0.22 (±0.01)	0.51 (±0.04)	0.60 (±0.04)	0.18 (±0.02)	0.48 (±0.04)	0.60 (±0.03)	0.22 (±0.01)	0.39 (±0.03)	0.58 (±0.05)	0.19 (±0.02)	0.37 (±0.02)	0.62 (±0.03)
sub11	0.25 (±0.02)	0.43 (±0.02)	0.58 (±0.05)	0.35 (±0.01)	0.41 (±0.03)	0.62 (±0.03)	0.24 (±0.02)	0.52 (±0.03)	0.58 (±0.04)	0.26 (±0.02)	0.40 (±0.02)	0.64 (±0.02)	0.27 (±0.02)	0.64 (±0.02)	0.58 (±0.05)	0.33 (±0.02)	0.59 (±0.02)	0.56 (±0.02)
sub12	0.21 (±0.02)	0.77 (±0.02)	0.56 (±0.02)	0.21 (±0.01)	0.93 (±0.02)	0.55 (±0.04)	0.22 (±0.01)	0.89 (±0.02)	0.56 (±0.03)	0.21 (±0.01)	0.94 (±0.02)	0.63 (±0.03)	0.21 (±0.02)	0.58 (±0.02)	0.58 (±0.04)	0.21 (±0.02)	0.56 (±0.02)	0.65 (±0.03)
sub13	0.20 (±0.01)	0.40 (±0.03)	0.58 (±0.06)	0.19 (±0.02)	0.44 (±0.04)	0.65 (±0.03)	0.22 (±0.02)	0.37 (±0.02)	0.61 (±0.04)	0.21 (±0.02)	0.35 (±0.03)	0.59 (±0.03)	0.19 (±0.02)	0.43 (±0.02)	0.56 (±0.04)	0.17 (±0.02)	0.37 (±0.04)	0.57 (±0.03)
sub14	0.22 (±0.02)	0.43 (±0.03)	0.52 (±0.01)	0.21 (±0.02)	0.46 (±0.03)	0.56 (±0.05)	0.23 (±0.02)	0.69 (±0.02)	0.54 (±0.02)	0.21 (±0.02)	0.42 (±0.03)	0.55 (±0.03)	0.21 (±0.01)	0.50 (±0.02)	0.56 (±0.02)	0.18 (±0.02)	0.45 (±0.02)	0.54 (±0.03)
sub15	0.21 (±0.01)	0.38 (±0.04)	0.61 (±0.03)	0.19 (±0.02)	0.40 (±0.04)	0.58 (±0.04)	0.21 (±0.02)	0.51 (±0.02)	0.63 (±0.03)	0.18 (±0.01)	0.43 (±0.03)	0.57 (±0.05)	0.20 (±0.02)	0.46 (±0.03)	0.61 (±0.04)	0.20 (±0.02)	0.41 (±0.02)	0.63 (±0.04)
sub16	0.21 (±0.02)	0.40 (±0.03)	0.63 (±0.04)	0.23 (±0.02)	0.43 (±0.02)	0.59 (±0.03)	0.20 (±0.01)	0.43 (±0.04)	0.59 (±0.03)	0.20 (±0.02)	0.36 (±0.03)	0.51 (±0.03)	0.19 (±0.03)	0.43 (±0.03)	0.55 (±0.04)	0.20 (±0.02)	0.37 (±0.04)	0.65 (±0.02)
sub17	0.25 (±0.02)	0.86 (±0.03)	0.56 (±0.04)	0.20 (±0.01)	0.34 (±0.03)	0.59 (±0.04)	0.23 (±0.01)	0.86 (±0.03)	0.65 (±0.03)	0.21 (±0.01)	0.36 (±0.03)	0.62 (±0.03)	0.27 (±0.01)	0.41 (±0.03)	0.56 (±0.04)	0.17 (±0.02)	0.39 (±0.04)	0.52 (±0.04)
sub18	0.22 (±0.01)	0.47 (±0.02)	0.64 (±0.03)	0.22 (±0.01)	0.36 (±0.02)	0.63 (±0.05)	0.21 (±0.01)	0.70 (±0.03)	0.64 (±0.03)	0.22 (±0.02)	0.50 (±0.03)	0.63 (±0.04)	0.24 (±0.02)	0.52 (±0.03)	0.58 (±0.03)	0.20 (±0.02)	0.37 (±0.01)	0.57 (±0.02)
sub19	0.22 (±0.02)	0.72 (±0.02)	0.61 (±0.03)	0.22 (±0.02)	0.39 (±0.03)	0.62 (±0.03)	0.22 (±0.02)	0.66 (±0.02)	0.50 (±0.03)	0.18 (±0.01)	0.40 (±0.03)	0.61 (±0.02)	0.19 (±0.02)	0.77 (±0.03)	0.64 (±0.03)	0.21 (±0.02)	0.40 (±0.02)	0.54 (±0.03)
sub20	0.43 (±0.01)	0.38 (±0.02)	0.56 (±0.04)	0.19 (±0.02)	0.41 (±0. 03)	0.58 (±0.03)	0.35 (±0.02)	0.60 (±0.03)	0.55 (±0.03)	0.21 (±0.01)	0.41 (±0.02)	0.59 (±0.04)	0.29 (±0.02)	0.42 (±0.03)	0.61 (±0.03)	0.20 (±0.02)	0.40 (±0.02)	0.54 (±0.04)
sub21	0.28 (±0.02)	0.45 (±0.04)	0.55 (±0.05)	0.20 (±0.02)	0.43 (±0.02)	0.60 (±0.02)	0.22 (±0.02)	0.45 (±0.04)	0.56 (±0.03)	0.21 (±0.02)	0.46 (±0.03)	0.53 (±0.02)	0.29 (±0.02)	0.53 (±0.02)	0.58 (±0.04)	0.19 (±0.02)	0.40 (±0.01)	0.58 (±0.02)
sub22	0.29 (±0.02)	0.73 (±0.02)	0.61 (±0.05)	0.23 (±0.02)	0.46 (±0.03)	0.60 (±0.03)	0.24 (±0.02)	0.46 (±0.02)	0.55 (±0.03)	0.21 (±0.02)	0.36 (±0.03)	0.61 (±0.03)	0.23 (±0.01)	0.60 (±0.03)	0.55 (±0.03)	0.21 (±0.02)	0.42 (±0.02)	0.55 (±0.03)
sub23	0.19 (±0.01)	0.45 (±0.03)	0.60 (±0.04)	0.21 (±0.02)	0.42 (±0.03)	0.57 (±0.04)	0.25 (±0.01)	0.36 (±0.03)	0.60 (±0.04)	0.20 (±0.02)	0.38 (±0.02)	0.57 (±0.04)	0.22 (±0.01)	0.52 (±0.03)	0.60 (±0.03)	0.21 (±0.01)	0.36 (±0.03)	0.57 (±0.03)
sub24	0.20 (±0.01)	0.35 (±0.03)	0.53 (±0.02)	0.23 (±0.01)	0.43 (±0.03)	0.61 (±0.04)	0.21 (±0.01)	0.47 (±0.04)	0.59 (±0.02)	0.20 (±0.02)	0.47 (±0.03)	0.58 (±0.03)	0.22 (±0.01)	0.57 (±0.03)	0.51 (±0.04)	0.22 (±0.03)	0.35 (±0.03)	0.56 (±0.01)
sub25	0.24 (±0.01)	0.49 (±0.04)	0.57 (±0.04)	0.19 (±0.01)	0.41 (±0.04)	0.61 (±0.04)	0.22 (±0.02)	0.64 (±0.03)	0.57 (±0.04)	0.20 (±0.01)	0.41 (±0.02)	0.58 (±0.04)	0.24 (±0.01)	0.58 (±0.03)	0.60 (±0.04)	0.21 (±0.01)	0.42 (±0.03)	0.60 (±0.03)
Average	0.23 (±0.05)	0.47 (±0.14)	0.59 (±0.03)	0.22 (±0.03)	0.44 (±0.11)	0.60 (±0.03)	0.23 (±0.03)	0.53 (±0.15)	0.58 (±0.04)	0.21 (±0.02)	0.45 (±0.14)	0.59 (±0.03)	0.22 (±0.03)	0.50 (±0.10)	0.58 (±0.03)	0.21 (±0.04)	0.43 (±0.09)	0.58 (±0.03)

raphy (sLORETA)-based current density estimation technique for inverse modeling the brain points that were activated from the EEG signals. sLORETA is a variant of the weighted minimum norm estimation technique for obtaining an inverse solution [31, 58]. We visualized the activated regions of the brain for each task and showed them in terms of the horizontal, sagittal, and coronal planes, as shown in Fig. 8(b). The source images were visualized by the significant differences by calculating the p-values for the spatial distribution between the baseline period (-0.5 to 0 s) and MI period (0 to 4 s). The yellow colors indicate the p-values below 0.01, and the red colors those below 0.05 [59].The main differences were observed in the supplementary motor region and premotor cortex; they indicate that the subjects satisfactorily performed MI. Since all the subjects performed MI related to the movement of the right upper limb, it can be confirmed from Fig. 8(b) that the left side region of the cortex associated with the MI is activated.

We evaluated the dataset quality by observing BCI classification performances (see Table 2). By using the baseline machine learning method, we confirmed that the accuracies were

at least higher than chance-level accuracy for each class. The performances were validated according to tasks including arm-reaching, hand-grasping, and wrist-twisting. We computed the chance-level accuracies with a significant confidence level (a = 5%) [60] and could obtain the chance results per evaluation as 0.17 (arm-reaching), 0.34 (hand-grasping), and 0.51 (wrist-twisting). Table 2 represents the classification accuracies with the standard deviation for each subject and recording session. Because our dataset was recorded over three different sessions, it allows for further research related to BCI-calibration problems. According to our classification result obtained using the baseline decoding method and conventional approach, some subjects showed a significant change in classification results when the session as changed; however, the other subjects showed similar classification accuracies over different sessions.

The decoding of intuitive upper-extremity movements from EEG signals is a challenging study. However, if the upper-extremity movements can be successfully analyzed, the BCI technology could be applied to many applications. The BCI may be applied to the operation of robotic instruments, such as a robotic arm and neuro-prosthesis related to upper-extremity movements, or to control peripheral devices using commands based on decoding the movement intention. In this study, for more advances, we provide a multimodal signal dataset when the subjects executed and imagined the intuitive movement tasks using a single arm. As mentioned in "*Experimental paradigm*", decoding various tasks from the same limb could provide various BCI classes and significantly intuitive communication between users and BCI systems as compared with a typical paradigm. Therefore, a robust decoding model for this dataset could contribute one step toward the advancement of a practical and commercial BCI. Therefore, one must obtain high-quality data. Additionally, more advanced analyses can be attempted because we have constructed a database that includes not only the EOG and EMG data but also the EEG data. For example, the EOG data might be used to remove the noise due to explicit eye movement from the EEG data. Additionally, the EMG data may demonstrate the integrity of the EEG data by showing that no movement-related interference was present in the EMG data in the analysis and MI tasks associated with the EEG. In this work, we recorded the signals' data using three modalities, namely, EEG, EOG, and EMG. Additionally, we collected data from 25 subjects and divided the experiment into three sessions to prepare the dataset. We also provided the spectral representation and time-spatial distribution of a representative subject according to multiple recording sessions. Generally, we confirmed that the data variability among each recording session did not show any significant differences in our dataset (i.e., p<0.05). Furthermore, we confirmed that the classification accuracy per task was slightly higher than the chance-level accuracy using the baseline method. Conversely, despite the difficult tasks involved in the experiments, the subjects successfully focused on the experiments so that we could obtain high-quality data. In the future, the users of this dataset can contribute to increasing the present classification accuracy using their novel methodology.

Through preliminary data validation, we determined the sufficient quality of our dataset as sufficient. Further studies can be performed to determine the hidden characteristics and features related to the intention of upper-limb movements using only our EEG data, while the EOG data can be used to filter noise for obtaining clear EEG signals. Simultaneously, researchers can attempt to combine EEG and EMG signals using our dataset for developing hybrid BCI systems. In related studies, the hybrid approaches showed remarkable possibility to improve the decoding performances of real-movement and MI-based BCIs [34, 61]. Additionally, our dataset can be used for studies that analyze the correlations between EEG and EMG. In related studies, the relevance of EEG and EMG signals can be found through the connectivity analysis of the data acquired over a specific period. For example, a statistical analysis of activated EEG channels conducted during the activation of a particular EMG channel can determine the region of the brain, channel location, and frequency band directly related to the movement of the particular muscle [62].

Inter-session comparisons are also important topics in BCI experiments. Since the BCI systems are recalibrated at the beginning of each recording session, this procedure becomes time-consuming and thus may limit the adoption of BCI systems for long-term daily usage [63]. Furthermore, we recorded data over three sessions to enable cross-session analysis. For each session, we collected a dataset of uniform quality on the basis of classification results (see Table 2), as we focused on conducting all the experiments under stable conditions. Researchers can analyze the decoding performance using our dataset from the entire session and they can also compare the decoding results of each session with each other. Different approaches are also available, and they include training the decoding model in a particular session and testing the model using data from independent sessions on the basis of the principle of transfer learning in BCI, as done in [64]. Accordingly, creating a session-independent BCI decoding model is critical to establishing a practical BCI system such as biometric authentication system [65] and brain-controlled AR/VR system [66]. Therefore, our experimental data can be useful for studies to build session-independent decoding models.

Availability of supporting data and materials

The data supporting this paper, including EEG, EMG, and EOG datasets and example codes, are available in the GigaScience database, GigaDB repository.

Abbreviations

BCI: brain-computer interface; EEG: electroencephalography; fNIRS: functional near-infrared spectroscopy; MI: motor imagery; SSVEP: steady-state visual evoked potential; ERP: event-related potential; MRCP:movement-related cortical potential; EMG: electromyography; EOG: electrooculography; LCD: liquid-crystal display; SMR: somatosensory rhythm; ERSP: event-related spectral perturbation; ICA: independent component analysis; ICs: independent components; CSP: common spatial pattern; RLDA: regularized linear discriminant analysis; ERD/ERS: event-related desynchronization/synchronization; sLORETA: standardized low-resolution electromagnetic tomography

Ethical Approval

This study was reviewed and approved by the Institutional Review Board at Korea University (1040548-KU-IRB-17-181-A-2).

Competing Interests

The authors declare no competing interests.

Funding

This work was partly supported by Institute of Information & Communications Technology Planning & Evaluation (IITP)

grant funded by the Korea government (No. 2015-0-00185, Development of Intelligent Pattern Recognition Softwares for Ambulatory Brain-Computer Interface; No. 2017-0-00451, Development of BCI based Brain and Cognitive Computing Technology for Recognizing User's Intentions using Deep Learning; No. 2019-0-00079, Department of Artificial Intelligence, Korea University).

Author's Contributions

J.-H. Jeong, J.-H. Cho, and K.-H. Shim designed the experimental protocols and paradigms. J.-H. Jeong, B.-H. Kwon, B.-H. Lee, D.-Y. Lee, and D.-H. Lee collected the data and checked the physical and mental states of the participant during the experiments. J.-H Jeong, J.-H. Cho, and S.-W. Lee revised the manuscript. All the authors analyzed and validated the collected data technically. Furthermore, the authors prepared the manuscript and approved the database public.

References

1. Kaufmann T, Kübler A. Beyond maximum speed—a novel two-stimulus paradigm for brain-computer interfaces based on event-related potentials (P300-BCI). J Neural Eng 2014;11(5):056004.
2. Kim KT, Suk HI, Lee SW. Commanding a brain-controlled wheelchair using steady-state somatosensory evoked potentials. IEEE Trans Neural Syst Rehabil Eng 2018;26(3):654–665.
3. Jeong JH, Shim KH, Kim DJ, et al. Trajectory decoding of arm reaching movement imageries for brain-controlled robot arm system. In: Proc. 41th Int. Conf. IEEE Eng. Med. Biol. Soc. (EMBC) Berlin, Germany; July 23 2019. p. 5544–5547.
4. Penaloza CI, Nishio S. BMI control of a third arm for multitasking. Sci Robot 2018;3(20):eaat1228.
5. Meng J, Zhang S, Bekyo A, et al. Noninvasive electroencephalogram based control of a robotic arm for reach and grasp tasks. Sci Rep 2016;6:38565.
6. Kwak NS, Müller KR, Lee SW. A lower limb exoskeleton control system based on steady state visual evoked potentials. J Neural Eng 2015;12(5):056009.
7. He Y, Eguren D, Azorín JM, et al. Brain–machine interfaces for controlling lower-limb powered robotic systems. J Neural Eng 2018;15(2):021004.
8. Abiri R, Borhani S, Sellers EW, et al. A comprehensive review of EEG-based brain-computer interface paradigms. J Neural Eng 2019;16(1):011001.
9. Wolpaw JR, Birbaumer N, McFarland DJ, et al. Brain–computer interfaces for communication and control. Clin Neurophysiol 2002;113(6):767–791.
10. Craik A, He Y, Contreras-Vidal JLP. Deep learning for Electroencephalogram (EEG) classification tasks: A review. J Neural Eng 2019;16(3):031001.
11. Kakkos I, Miloulis ST, Gkiatis K, et al. Human–machine interfaces for motor rehabilitation. Adv Comput Intell in Healthcare-7 2020;p. 1–16.
12. Lee MH, Fazli S, Mehnert J, et al. Subject-dependent classification for robust idle state detection using multi-modal neuroimaging and data-fusion techniques in BCI. Patt Recognit 2015;48(8):2725–2737.
13. Ang KK, Guan C. EEG-based strategies to detect motor imagery for control and rehabilitation. IEEE Trans Neural Syst Rehabil Eng 2017;25(4):392–401.
14. Tabar YR, Halici U. A novel deep learning approach for classification of EEG motor imagery signals. J Neural Eng 2016;14(1):016003.
15. Lu N, Li T, Ren X, et al. A deep learning scheme for motor imagery classification based on restricted Boltzmann machines. IEEE Trans Neural Syst Rehabil Eng 2017;25(6):566–576.
16. Won DO, Hwang HJ, Dähne S, et al. Effect of higher frequency on the classification of steady-state visual evoked potentials. J Neural Eng 2015;13(1):016014.
17. Kwak NS, Müller KR, Lee SW. A convolutional neural network for steady state visual evoked potential classification under ambulatory environment. PloS One 2017;12(2):e0172578.
18. Yeom SK, Fazli S, Müller KR, et al. An efficient ERP-based brain-computer interface using random set presentation and face familiarity. PloS One 2014;9(11).
19. Shakeel A, Navid MS, Anwar MN, et al. A review of techniques for detection of movement intention using movement-related cortical potentials. Comput Math Method M 2015;2015.
20. Jeong JH, Kwak NS, Guan C, et al. Decoding movement-related cortical potentials based on subject-dependent and section-wise spectral filtering. IEEE Trans Neural Syst Rehabil Eng 2020;28(3):687–698.
21. Kaya M, Binli MK, Ozbay E, et al. A large electroencephalographic motor imagery dataset for electroencephalographic brain computer interfaces. Sci Data 2018;5:180211.
22. Cao Z, Chuang CH, King JK, et al. Multi-channel EEG recordings during a sustained-attention driving task. Sci Data 2019;6(1):1–8.
23. Lee MH, Kwon OY, Kim YJ, et al. EEG dataset and OpenBMI toolbox for three BCI paradigms: an investigation into BCI illiteracy. GigaScience 2019;8(5):giz002.
24. Choi GY, Han CH, Jung YJ, et al. A multi-day and multi-band dataset for a steady-state visual-evoked potential–based brain-computer interface. GigaScience 2019;8(11):giz133.
25. Cho H, Ahn M, Ahn S, et al. EEG datasets for motor imagery brain–computer interface. GigaScience 2017;6(7):gix034.
26. Shiman F, López-Larraz E, Sarasola-Sanz A, et al. Classification of different reaching movements from the same limb using EEG. J Neural Eng 2017;14(4):046018.
27. Ofner P, Schwarz A, Pereira J, et al. Upper limb movements can be decoded from the time-domain of low-frequency EEG. PloS One 2017;12(8).
28. Hossain I, Khosravi A, Hettiarachchi I, et al. Batch mode query by committee for motor imagery-based BCI. IEEE Trans Neural Syst Rehabil Eng 2019;27(1):13–21.
29. Kwon OY, Lee MH, Guan C, Lee SW. Subject-independent brain-computer interfaces based on deep convolutional neural networks. IEEE Trans Neural Netw Learn Syst 2019;p. 1–14.
30. Lee BH, Jeong JH, Lee SW. SessionNet: Feature similarity-based weighted ensemble learning for motor imagery classification. IEEE Access 2020;8:134524–134535.
31. Edelman BJ, Baxter B, He B. EEG source imaging enhances the decoding of complex right-hand motor imagery tasks. IEEE Trans Biomed Eng 2015;63(1):4–14.
32. Leske S, Dalal SS. Reducing power line noise in EEG and MEG data via spectrum interpolation. NeuroImage 2019;189:763–776.
33. Jeong JH, Shim KH, Kim DJ, et al. Brain-controlled robotic arm System based on multi-directional CNN-BiLSTM network using EEG signals. IEEE Trans Neural Syst Rehabil Eng 2020;28(5):1226–1238.
34. Li X, Samuel OW, Zhang X, et al. A motion-classification strategy based on sEMG-EEG signal combination for upper-limb amputees. J Neuroeng Rehabil 2017;14(1):2.
35. Furui A, Hayashi H, Nakamura G, et al. An artificial

EMG generation model based on signal-dependent noise and related application to motion classification. PloS One 2017;12(6).
36. Hussain J, Sundaraj K, Subramaniam ID, et al. Muscle fatigue in the three heads of triceps brachii during intensity and speed variations of triceps push-down exercise. Front Physiol 2020;11(112):1–13.
37. Ma J, Zhang Y, Cichocki A, et al. A novel EOG/EEG hybrid human–machine interface adopting eye movements and ERPs: Application to robot control. IEEE Trans Biomed Eng 2014;62(3):876–889.
38. Blankertz B, Tangermann M, Vidaurre C, et al. The Berlin brain–computer interface: non-medical uses of BCI technology. Front Neurosci 2010;4:198.
39. Delorme A, Makeig S. EEGLAB: an open source toolbox for analysis of single-trial EEG dynamics including independent component analysis. J Neurosci Methods 2004;134(1):9–21.
40. Pfurtscheller G, Neuper C. Motor imagery and direct brain-computer communication. Proc IEEE 2001;89(7):1123–1134.
41. Sreeja S, Rabha J, Samanta D, et al. Classification of motor imagery based EEG signals using sparsity approach. In: 9th Int. Conf. Intell. Hum. Com. Interact. (IHCI) Paris, France; Dec. 2017. p. 47–59.
42. Singh B, Wagatsuma H. A removal of eye movement and blink artifacts from EEG data using morphological component analysis. Comput Math Method M 2017;2017.
43. Bell AJ, Sejnowski TJ. An information-maximization approach to blind separation and blind deconvolution. Neural Comput 1995;7(6):1129–1159.
44. Kachenoura A, Albera L, Senhadji L, et al. ICA: a potential tool for BCI systems. IEEE Signal Process Mag 2007;25(1):57–68.
45. Blankertz B, Tomioka R, Lemm S, et al. Optimizing spatial filters for robust EEG single-trial analysis. IEEE Signal Process Mag 2007;25(1):41–56.
46. Rozado D, Duenser A, Howell B. Improving the performance of an EEG-based motor imagery brain computer interface using task evoked changes in pupil diameter. PloS One 2015;10(3):e0121262.
47. Treder MS, Porbadnigk AK, Avarvand FS, et al. The LDA beamformer: optimal estimation of ERP source time series using linear discriminant analysis. Neuroimage 2016;129:279–291.
48. Mkhadri A. Shrinkage parameter for the modified linear discriminant analysis. Pattern Recognit Lett 1995;16(3):267–275.
49. Trigili E, Grazi L, Crea S, et al. Detection of movement onset using EMG signals for upper-limb exoskeletons in reaching tasks. J Neuroeng Rehabil 2019;16(1):45.
50. Winiarski D. The use of EMG signal in human-machine interface. Automatyka/automatics 2015;19(2):47–61.
51. Wang J, Tang L, Bronlund JE. Surface EMG signal amplification and filtering. Int J Comput Appl 2013;82(1):15–22.
52. Yao L, Mrachacz-Kersting N, Sheng X, et al. A multi-class BCI based on somatosensory imagery. IEEE Trans Neural Syst Rehabil Eng 2018;26(8):1508–1515.
53. Tang N, Guan C, Ang K, et al. Motor imagery-assisted brain-computer interface for gait retraining in neurorehabilitation in chronic stroke. Ann Phys Rehabil Med 2018;61:e188.
54. Kaiser V, Kreilinger A, Müller-Putz GR, et al. First steps toward a motor imagery based stroke BCI: new strategy to set up a classifier. Front Neurosci 2011;5:86.
55. Müller-Putz G, Scherer R, Pfurtscheller G, et al. Temporal coding of brain patterns for direct limb control in humans. Front Neurosci 2010;4:34.
56. Blankertz B, Acqualagna L, Dähne S, et al. The Berlin brain-computer interface: progress beyond communication and control. Front Neurosci 2016;10:530.
57. Amin HU, Malik AS, Kamel N, et al. P300 correlates with learning memory abilities and fluid intelligence. J Neuroeng Rehabil 2015;12(1):87.
58. Handiru VS, Vinod A, Guan C. EEG source space analysis of the supervised factor analytic approach for the classification of multi-directional arm movement. J Neural Eng 2017;14(4):046008.
59. Canuet L, Ishii R, Pascual-Marqui RD, et al. Resting-state EEG source localization and functional connectivity in schizophrenia-like psychosis of epilepsy. PloS One 2011;6(11):e27863.
60. Müller-Putz G, Scherer R, Brunner C, et al. Better than random: a closer look on BCI results. Int J Bioelectromagn 2008;10(1):52–55.
61. Bakshi K, Pramanik R, Manjunatha M, et al. Upper limb prosthesis control: A hybrid EEG-EMG scheme for motion estimation in transhumeral subjects. In: Proc. 40th Int. Conf. IEEE Eng. Med. Biol. Soc. (EMBC) Hawaii, USA; July 17 2018. p. 2024–2027.
62. Cho JH, Jeong JH, Shim KH, et al. Classification of various grasping tasks based on temporal segmentation method using EEG and EMG signals. In: GBCIC Graz, Austria; Sep. 16 2019. p. 1–6.
63. Abu-Rmileh A, Zakkay E, Shmuelof L, et al. Co-adaptive training improves efficacy of a multi-day EEG-based motor imagery BCI training. Front Hum Neurosci 2019;13:362.
64. Azab AM, Mihaylova L, Ang KK, et al. Weighted transfer learning for improving motor imagery-based brain-computer interface. IEEE Trans Neural Syst Rehabil Eng 2019;27(7):1352–1359.
65. Chen Y, Atnafu AD, Schlattner I, et al. A high-security EEG-based login system with RSVP stimuli and dry electrodes. IEEE Trans Inf Forensics and Secur 2016;11(12):2635–2647.
66. Putze F, Vourvopoulos A, Lécuyer A, et al. Brain-computer interfaces and Augmented/Virtual Reality. Front Hum Neurosci 2020;14.

Click here to access/download
Supplementary Material
Reply To Reviewers Comments.pdf
