# Supplementary material for: Multimodal signal dataset for 11 intuitive movement tasks from single upper extremity during multiple recording sessions
Source: Gigascience. 2020 Oct 7;9(10):giaa098. doi: 10.1093/gigascience/giaa098 (PMC7539536; doi:10.1093/gigascience/giaa098)
Supplement: giaa098_GIGA-D-20-00075_Revision_2 [file giaa098_giga-d-20-00075_revision_2.pdf]

# GigaScience

# Multimodal signal dataset for 11 intuitive movement tasks from single upper extremity during multiple recording sessions

--Manuscript Draft--

| | |
|---|---|
| **Manuscript Number:** | GIGA-D-20-00075R2 |
| **Full Title:** | Multimodal signal dataset for 11 intuitive movement tasks from single upper extremity during multiple recording sessions |
| **Article Type:** | Data Note |
| **Funding Information:** | Institute of Information & Communications Technology Planning & Evaluation (IITP) grant (2015-0-00185) — Mr. Ji-Hoon Jeong<br>Institute of Information & Communications Technology Planning & Evaluation (IITP) grant (2017-0-00451) — Dr. Seong-Whan Lee<br>Institute of Information & Communications Technology Planning & Evaluation (IITP) grant (2019-0-00079) — Dr. Seong-Whan Lee |
| **Abstract:** | Background: Non-invasive brain-computer interfaces (BCIs) have been developed for realizing natural bi-directional interaction between users and external robotic systems. However, the communication between users and BCI systems through artificial matching is a critical issue. Recently, BCIs have been developed to adopt intuitive decoding which is the key to solving several problems such as a small number of classes and manually matching BCI commands with device control. Unfortunately, the advances in this area have been slow owing to the lack of large and uniform datasets. This study provides a large intuitive dataset for 11 different upper-extremity movement tasks obtained during multiple recording sessions. The dataset includes 60-channel electroencephalography (EEG), 7-channel electromyography (EMG), and 4-channel electrooculography (EOG) of 25 healthy subjects collected over 3-day sessions for a total of 82,500 trials across all the subjects. Findings: We validated our dataset via neuro physiological analysis. We observed clear sensorimotor de-/activation and spatial distribution related to real-movement and motor imagery (MI), respectively. Furthermore, we demonstrated the consistency of the dataset by evaluating the classification performance of each session using a baseline machine learning method. Conclusions: The dataset includes the data of multiple recording sessions, various classes within the single upper-extremity, and multimodal signals. This work can be used to i) compare the brain activities associated with real-movement and imagination, ii) improve the decoding performance, and iii) analyze the differences among recording sessions. Hence, this study, as a data note, has focused on collecting data required for further advances in the BCI technology. |
| **Corresponding Author:** | Seong-Whan Lee<br>Korea University<br>Seoul, KOREA, REPUBLIC OF |
| **Corresponding Author Secondary Information:** | |
| **Corresponding Author's Institution:** | Korea University |
| **Corresponding Author's Secondary Institution:** | |
| **First Author:** | Ji-Hoon Jeong |
| **First Author Secondary Information:** | |
| **Order of Authors:** | Ji-Hoon Jeong<br>Jeong-Hyun Cho<br>Kyung-Hwan Shim<br>Byoung-Hee Kwon |

Powered by Editorial Manager® and ProduXion Manager® from Aries Systems Corporation

| | |
|---|---|
| | Byeong-Hoo Lee |
| | Do-Yeun Lee |
| | Dae-Hyeok Lee |
| | Seong-Whan Lee |
| Order of Authors Secondary Information: | |
| Response to Reviewers: | When we have used the Gigascience latex template in overleaf, the DOI doesn't represent in the manuscript. Therefore, we added DOI as URL in the BibTeX. Please confirm this issue.<br><br>In addition, I cannot upload the latex files as .zip file. Therefore, I uploaded the files separately. Please also confirm this issue too. |
| Additional Information: | |
| Question | Response |
| Are you submitting this manuscript to a special series or article collection? | No |
| **Experimental design and statistics**<br><br>Full details of the experimental design and statistical methods used should be given in the Methods section, as detailed in our Minimum Standards Reporting Checklist. Information essential to interpreting the data presented should be made available in the figure legends.<br><br>Have you included all the information requested in your manuscript? | Yes |
| **Resources**<br><br>A description of all resources used, including antibodies, cell lines, animals and software tools, with enough information to allow them to be uniquely identified, should be included in the Methods section. Authors are strongly encouraged to cite Research Resource Identifiers (RRIDs) for antibodies, model organisms and tools, where possible.<br><br>Have you included the information requested as detailed in our Minimum Standards Reporting Checklist? | Yes |
| **Availability of data and materials** | Yes |

Powered by Editorial Manager® and ProduXion Manager® from Aries Systems Corporation

| | |
|---|---|
| All datasets and code on which the conclusions of the paper rely must be either included in your submission or deposited in publicly available repositories (where available and ethically appropriate), referencing such data using a unique identifier in the references and in the "Availability of Data and Materials" section of your manuscript.<br><br>Have you have met the above requirement as detailed in our Minimum Standards Reporting Checklist? | |

Manuscript revised with DOI

Click here to access/download;Manuscript;Lee_Brain_R2.pdf

Placeholder for OUP logo oup.pdf

Placeholder for journal logo gigascience-logo.pdf

*GigaScience*, 2020, 1–13

doi: xx.xxxx/xxxx
**Manuscript in Preparation**
**Data Note**

DATA NOTE

# Multimodal signal dataset for 11 intuitive movement tasks from single upper-extremity during multiple recording sessions

Ji-Hoon Jeong[1], Jeong-Hyun Cho[1], Kyung-Hwan Shim[1], Byoung-Hee Kwon[1], Byeong-Hoo Lee[1], Do-Yeun Lee[1], Dae-Hyeok Lee[1] and Seong-Whan Lee[1,2,*]

[1]Department of Brain and Cognitive Engineering, Korea University, 145 Anam-ro, Seongbuk-gu, Seoul 02841, South Korea and [2]Department of Artificial Intelligence, Korea University, 145 Anam-ro, Seongbuk-gu, Seoul 02841, South Korea

[*]**Correspondence address**. Seong-Whan Lee, Tel: +82-2-3290-3197; Fax: +82-2-3290-3583; E-mail: sw.lee@korea.ac.kr

## Abstract

**Background**: Non-invasive brain-computer interfaces (BCIs) have been developed for realizing natural bi-directional interaction between users and external robotic systems. However, the communication between users and BCI systems through artificial matching is a critical issue. Recently, BCIs have been developed to adopt intuitive decoding which is the key to solving several problems such as a small number of classes and manually matching BCI commands with device control. Unfortunately, the advances in this area have been slow owing to the lack of large and uniform datasets. This study provides a large intuitive dataset for 11 different upper-extremity movement tasks obtained during multiple recording sessions. The dataset includes 60-channel electroencephalography (EEG), 7-channel electromyography (EMG), and 4-channel electrooculography (EOG) of 25 healthy subjects collected over 3-day sessions for a total of 82,500 trials across all the subjects. **Findings**: We validated our dataset via neuro-physiological analysis. We observed clear sensorimotor de-/activation and spatial distribution related to real-movement and motor imagery (MI), respectively. Furthermore, we demonstrated the consistency of the dataset by evaluating the classification performance of each session using a baseline machine learning method. **Conclusions**: The dataset includes the data of multiple recording sessions, various classes within the single upper-extremity, and multimodal signals. This work can be used to *i*) compare the brain activities associated with real-movement and imagination, *ii*) improve the decoding performance, and *iii*) analyze the differences among recording sessions. Hence, this study, as a data note, has focused on collecting data required for further advances in the BCI technology.

**Key words**: brain-computer interface (BCI); multimodal signals; intuitive upper-extremity movements; multiple sessions

## Data Description

### Background and purpose

The brain-computer interface (BCI) technology allows users to communicate with external devices including a speller [1], wheelchair [2], robotic arm [3–5] and robotic exoskeleton [6, 7]. A non-invasive BCI commonly employs electroencephalography (EEG) signals to decode user intentions [8–11] because the EEG-based BCI system offers lower risk, lower cost, and more convenient than other non-invasive BCI paradigms (e.g., functional near-infrared spectroscopy (fNIRS) [12]). EEG-based

**Compiled on:** September 3, 2020.
Draft manuscript prepared by the author.

BCIs have been developed using various paradigms including motor imagery (MI) [13–15], steady-state visual evoked potential (SSVEP) [16, 17], event-related potential (ERP) [18], and movement-related cortical potential (MRCP) [19, 20]. Over the past decades, the information regarding the EEG datasets of these general paradigms have been published through competitions, cooperation projects, and open-access articles [21–25]. Some research groups have developed advanced machine learning algorithms and deep learning architectures for improving the BCI performance using these datasets.

The recent advances in BCI systems have been focused on topics ranging from intuitive EEG decoding to directly matching the interaction between user intention and device feedback for real-world environments [20, 26]. For example, to control a neuro-prosthetic arm using typical BCI paradigms, we temporarily matched BCI commands with robotic arm motions (e.g., MI for both hands = grasping motion of the robotic hand). However, this unintended artificial matching suffers from several constraint limitations, such as the small number of restrictive classes for communicating with devices and inflexible user training due to unintuitive commands in real-world scenarios [7, 13, 27]. For example, if a robotic hand performs the grasping motion, the users should also imagine the hand grasping motion the same as the robotic hand motion to enhance the natural decoding experience [27].

In this work, we collected data on intuitive upper-extremity movements from 25 subjects. To collect high-quality signal data, the experiments were conducted on healthy subjects, who had maintained good physical conditions by, for instance, limiting their alcohol intake and getting sufficient sleep. We focused on various upper-extremity motions because they are the most extensible and available movements among all the body movements. Accordingly, we selected the upper extremities for decoding intuitive movements and then collected data based on the movement-based multimodal signals. The subjects were asked to perform 11 different movement tasks: arm-reaching along six directions, hand-grasping of three objects, and wrist-twisting with two different motions. The corresponding 11 classes were designed for each segmented motion related to the arm, hand, and wrist, rather than for continuous limb movements. Therefore, the users of our dataset could either conduct respective analyses for individual classes or attempt decoding the complex upper-extremity movements by combining data from different classes. For researchers focused on more advanced and analytical approaches using multimodal signals, the dataset comprised not only EEG data but also electromyography (EMG) and electrooculography (EOG) data. These data were synchronously collected in the same experimental environment, while ensuring no unintentional interference between them. The data acquired using a 60-channel EEG, 7-channel EMG, and 4-channel EOG were simultaneously recorded during the experiment. EEG sensors were placed according to international specifications to collect signals from all the regions of the scalp. Additionally, EMG sensors were attached to carefully selected locations on the right arm to reflect the most relevant muscle-activity information associated with the corresponding upper limb movement. We also recorded the EOG signals using four channels independent of the EEG channels to capture detailed eye movements, which were mainly used for artifact removal. The subjects performed real upper-extremity movements and MI associated with the 11 aforementioned motions. Additionally, each subject participated in three recording sessions at one-week intervals and followed the same experimental protocols. To acquire a large-amount of high-quality data, we prioritized the physical and mental conditions of the subjects as a priority during the experiments. Eventually, the multimodal signal dataset became sufficiently large for the BCI experiment because it now included data acquired from 82,500 trials performed for all the subjects (i.e., 3,300 trials were collected per subject).

To the best of our knowledge, the present dataset descriptor is the first large public dataset for intuitive BCI paradigms to include multimodal signals such as EEG, EMG, and EOG signals. This study might contribute to the realization of reliable neuro-rehabilitation of patients with motor disabilities and a high-level BCI system for healthy users. Furthermore, to ensure the practicality of the BCI technology, we intend to investigate how to robustly decode motor-related intentions despite different recording sessions and subject dependency (i.e., the session-to-session problem [23, 28] and subject-independent problem [29]). However, presently, only few datasets exist to be applied to various types of real-world applications and to develop a robust neural decoding model. To overcome this challenge, this study could contribute to the development of a practical BCI system based on deep learning techniques and multi-modalities by providing a large dataset.

## Experimental design

### *Subjects*

Twenty-five subjects (all-right handed, S1-S25, aged 24-32 years, 15 males and 10 females) who were naïve BCI users participated in the experiments. They were healthy individuals with no known neurophysiological anomalies or musculoskeletal disorders. Before the experiments, they were informed about the experimental protocols, paradigms, and purpose. After ensuring that they had understood the information, they provided their written consent according to the Declaration of Helsinki. The subjects signed a form that agreed to the anonymous public release of their data. We checked their physical and mental states for comparing the influence of the BCI performance according to individual state. Additionally, each subject was required to be in normal health, get sufficient sleep (approximately 8 h), and avoid alcohol, caffeinated drinks, and strenuous physical activity before the experiments. All the experimental protocols and environments were reviewed and approved by the Institutional Review Board (IRB) at Korea University (1040548-KU-IRB-17-181-A-2).

### *Environment*

During the experiments, each subject was comfortably seated in a chair with armrests facing the front of an LCD monitor, approximately 80 ($\pm$5)cm away from each other [30]. An EEG cap (Fig. 1) with 60 channels (actiCap, BrainProduct GmbH, Germany) was placed on the head of each subject. Surface EMG and EOG electrodes were attached to the pre-assigned locations on the right arm and around the eyes of each subject, respectively. The subjects were then asked to perform the movements with relaxed muscles and minimum eye and body movements during the data recording.

The duration of the experiment was approximately 6-7 h a day. Our experiment comprised multiple recording sessions (three days) to consider inter-session and inter-subject variabilities. Compared with typical BCI experiments, our experiments required a longer recording time. To maintain the physical and mental conditions of the subjects and thus ensure high signal quality, the subjects took sufficient breaks between each task. During the breaks, we first confirmed the physical and mental conditions of the subjects through self-report. If they reported of any inconvenient position or unstable conditions, we either adjusted the experimental environment according to their requests or halted the experiment. In the case the experiment was halted, the subjects could ask to conduct the experiment next time or not perform the experiment altogether. However, if the conditions of the subjects were good to con-

Figure/GIGA_Figure1.pdf

**Figure 1.** Experimental environments for acquiring multimodal signals related to the intuitive movement tasks. The subjects were asked to perform real-movement (e.g., arm-reaching) and MI tasks (e.g., hand-grasping).

duct the experiment, we checked the impedances of the EEG, EMG, and EOG electrodes and injected electrolyte gel into them to maintain the impedance values below 15 k$\Omega$. Therefore, we attempted to obtain clear signals excluding the spontaneous noise due to muscle and mental fatigue during the recording.

### *Experimental paradigm*

The experiment was designed to quantitatively acquire data related to the 11 different upper-extremity movements for both real-movement and MI tasks. The subjects conducted the experimental tasks using the same limbs. Decoding different tasks related to the same limb by using EEG signals could increase the number of possibilities of controlling the BCI system compared with typical somatosensory rhythm (SMR)-based BCIs which often only detected left-/right-hand and foot imagery [27]. The experimental tasks comprised three main upper-extremity motions: arm-reaching, hand-grasping, and wrist-twisting. When the experiment began, visual instructions were provided on the monitor by displaying a black cross sign and a gray background. The subjects stared at the visual instructions for 4 s while resting. After resting, a visual cue was displayed on the monitor with a text sign for 3 s, following which the subjects began preparing to perform the real-movement or MI tasks according to the visual cue (see Fig. 2). Upon changing the visual cue to a text sign as "Movement execution" and "Movement imagery", the subjects performed the corresponding tasks during 4 s. During the real-movement tasks, the subjects were asked to focus on the sensations involved with each motion and to remember those sensations for the MI tasks.

Arm-reaching along with six directions: The subjects were asked to perform multi-direction arm-reaching tasks directed from the center of their bodies to the outward direction. They performed the tasks along six different directions in the 3D

Figure/GIGA_Figure2.pdf

**Figure 2.** Experimental paradigm in a single-trial and the representation of visual cues according to each task.

Figure/GIGA_Figure3.pdf

**Figure 3.** Experimental tasks of 11 intuitive upper-extremity movements related to arm-reaching, hand-grasping, and wrist-twisting, respectively.

space: forward, backward, left, right, up, and down, as depicted in Fig. 3. In the real-movement tasks, the subjects extended their arms along one of the directions. The arm-reaching paradigm required 50 trials along each direction so that data could be collected for a total of 300 trials. However, in the MI tasks, the subjects only imagined performing an arm-reaching task; the number of trials in the MI paradigm was the same as in the real-movement paradigm.

Hand-grasping three objects: The subjects were asked to grasp three objects of daily use via the corresponding grasping motions. They performed the three designated grasp motions by holding the objects, namely, card, ball, and cup, corresponded to cylindrical, spherical, and lateral grasp, respectively (see Fig. 3). In the real-movement tasks, we asked the subjects to use their right hands to grasp a randomly selected object and hold it using its corresponding grasping motion. Eventually, we acquired data on 50 trials for each grasp, and hence, we collected 150 trials per subject. In the MI tasks, the subjects performed only one of the three grasping motions per trial, randomly. The number of trials in the MI paradigm was the same as that in the real-movement paradigm.

Wrist-twisting with two different motions: For the wrist-twisting tasks, the subjects rotated their wrists to the left (pronation) and right (supination), as depicted in Fig. 3. During real-movement task, each subject maintained his/her right hand in a neutral position with the elbow comfortably placed on the desk. Notably, wrist pronation and supination are complex actions used to decode user intentions from brain signals. Additionally, these movements are intuitive motions for realizing neuro-rehabilitation and prosthetic control [31]. We collected data for 50 trials per motion (i.e., total 100 trials) per day, and the visual cues were randomly displayed.

Additionally, the subjects were asked to participate in three recording sessions with a one-week interval between each session. The experimental environment and protocols were the same for all the three sessions. Consequently, we collected data from 3,300 trials (1,800 trials for arm-reaching, 900 for hand-grasping, and 600 for wrist-twisting) in all classes per subject, both for real-movement and MI paradigms.

## Data Records

We simultaneously collected three different kinds of physiological signals, namely, EEG, EMG, and EOG signals for 11 different upper-extremity movements (see Fig. 3). During the experiment, the signals were acquired using the same digital amplifier and types of electrodes. Therefore, the raw signals were stored together in one data file according to each subject. To obtain high-quality signals, the impedances of all the channels were maintained to be below 15 kΩ. After applying the conductive gel to the electrodes, we validated the accuracy of the EEG and EOG signals by asking the subjects to blink and close their eyes. The eye-blinking task was used to identify strong spikes in the frontal EEG channels (e.g., Fp1 and Fp2) and 4 EOG channels. The eye-closing task was used to confirm the alpha oscillations in the occipital channels (e.g., O1, O2, and Oz). We also asked the subjects to perform a simple hand-grasping motion to confirm the strong spikes in the EMG signals.

### *EEG signals*

The EEG data were recorded in conjunction with an EEG signal amplifier (BrainAmp, BrainProduct GmbH, Germany), sampled at 2,500 Hz. Additionally, we applied a 60 Hz with a notch filter to reduce the effect of external electrical noises (e.g., DC noise due to power supply, scan rate of the monitor display, and frequency of the fluorescent lamp) in raw signals [21, 32, 33]. The raw data were recorded using BrainVision (BrainProduct GmbH, Germany) with MATLAB 2019a (The MathWorks Inc., USA). Furthermore, a toal of 60 EEG electrodes were selected by following a 10-20 international configuration (Fp1-2, AF5-6, AF7-8, AFz, F1-8, Fz, FT7-8, FC1-6, T7-8, C1-6, Cz, TP7-8, CP1-6, CPz, P1-8, Pz, PO3-4, PO7-8, POz, O1-2, Oz, and Iz). Ground and reference channels were placed on the Fpz and FCz, respectively (see Fig. 4). The impedances of all the electrodes between the sensors and scalp skin were maintained to be below 15 kΩ. During the break time, conductive gel was injected into the electrodes using a syringe with a blunt needle.

### *EMG signals*

The EMG signals were recorded using 7 Ag/AgCl electrodes from the digital amplifier, the same equipment used to record

Figure/GIGA_Figure4.pdf

**Figure 4.** Data configuration for 60 EEG, 7 EMG, and 4 EOG channels. Specifically, 4 EOG channels were numbered as 32-35 out of the 64-channel actiCAP montage.

the EEG signals. We simultaneously acquired the EMG and EEG signals using the same amplifer [34]. The signals were captured at a sampling rate of 2,500 Hz with a 60 Hz notch filter same as the setting used to record the EEG signals. The EMG data were recorded from six related muscles for right arm movement: extensor carpi ulnaris, extensor digitorum, flexor carpi radialis, flexor carpi ulnaris, biceps brachii, and triceps brachii (see Fig. 4) [35]. The ground and reference were recorded in Fpz and FCz, respectively, which are the same as the EEG and EOG signals. The last electrode was placed on the elbow of the right arm which is a non-muscle movement area, as an alternative reference signal [36]. The purpose of recording EMG signals was to detect muscle activities when the subjects performed the designated tasks. The signals could prove that the subjects performed MI tasks without muscle movement. Simultaneously, the electrodes were placed so as to record sufficient number of signals from various arm and hand movements (i.e., six arm-reaching, three hand-grasping, and two wrist-twisting motions).

### *EOG signals*

The EOG signals were recorded using 4 channels while following the same protocol. Subsequently, the FT9, FT10, TP9, and TP10 electrodes were moved to the region around the eyes to function as EOG channels to eliminate the artifacts due to ocular activities. One of these channels was moved to the region around the left eye and the others to the region around the right eye (see Fig. 4). The electrodes $EOG_1$ and $EOG_4$ were used to record horizontal eye movements, while $EOG_2$ and $EOG_3$ were used to record vertical movements [37]. A medical tape was used to hold the sensors around the eyes and maintain the impedances of all the electrodes to be below $15k\Omega$.

**Table 1.** Data description for *Raw data* folder and *Converted data* folder

| Raw data | | Converted data | | |
|---|---|---|---|---|
| | | (e.g., session1_sub1_multigrasp_MI.mat) | | |
| Name | Description | Name | | Description |
| session1_sub1_(*Task*)_(realMove/MI).eeg | Raw signals (session I) | .dat | .x | Pre-processed data |
| session1_sub1_(*Task*)_(realMove/MI).vhdr | Data header information (session I) | | .fs | Sampling frequency |
| session1_sub1_(*Task*)_(realMove/MI).vhdr | Marker information for (session I) | | .file | File name |
| | | | .clab | Channel information |
| | | .mnt | .x | X coordinates for channel position |
| session2_sub1_(*Task*)_(realMove/MI).eeg | Raw signals (session II) | | .y | Y coordinates for channel position |
| session2_sub1_(*Task*)_(realMove/MI).vhdr | Data header information (session II) | | .pos_3d | 3D coordinates for channel position |
| session2_sub1_(*Task*)_(realMove/MI).vhdr | Marker information for (session II) | | .clab | Channel information |
| | | .mrk | .pos | Trigger marking time |
| | | | .toe | Trigger number |
| session3_sub1_(*Task*)_(realMove/MI).eeg | Raw signals (session III) | | .fs | Sampling frequency |
| session3_sub1_(*Task*)_(realMove/MI).vhdr | Data header information (session III) | | .y | Class lebels |
| session3_sub1_(*Task*)_(realMove/MI).vmrk | Marker information for (session III) | | .className | Class name |
| | | | .mics | Experiment start and end information |

### *Data format and structure*

Readers can access our codes and datasets through the GigaDB repository. The "*Read me*".pdf file, which overviews of data description and code execution, is included in the repository. Several useful scripts, including *Data_analysis*.m and *Visualization*.m files, are in the "SampleCode" folder. We recommend the BBCI (http://www.bci.de) toolbox [38] in the "Reference toolbox" folder and the "Signal Processing" toolbox in the MATLAB software, for data processing using our custom code. Please directly contact the authors for more information on the code script.

Each dataset (raw signals, converted data, and scripts) is also publicly available via the GigaDB repository. The raw signals and converted data are contained in the corresponding folders, namely, "Raw data" and "Converted data", respectively. Table 1 summarizes the data description for both the folders. The indicated *Task* includes the names of arm-reaching, multigrasp, and twisting. We provide the folders, namely,: "Raw data" and "Converted data" which comprise *.eeg*, *.vmrk*, *.vhdr*, and *.mat* files for each subject. The *.eeg* file includes the raw EEG, EOG, and EMG signals data because of the simultaneous data acquisition performed using the same amplifier. Moreover, the *.vmrk* file provides the marked trigger information (e.g., trigger number, marked time, and file name) and the *.vhdr* file includes the number of channels, sampling rate, channel position, and electrode impedances. The *.mat* file includes pre-processed EEG, EOG, and EMG data, channels, class information, scalp montage, and sample frequency. Additionally, to enable easy data access for the users, we provided the dataset after converting the .eeg file to .mat file in the "Converted data" folder. The converted .mat file includes some information such as trigger mark information, channel configuration, epoch segmentation, and etc.

*Data_Analysis*.m provides the basic data-processing script, which includes data loading, signal pre-processing, artifact rejection, feature extraction, classification, and performance evaluation. All users can download and unzip the '*SampleData*'.zip file contained in the "SampleData" folder before executing each code.

*Visualization*.m enables the visualization of raw signals, scalp distribution, and event-related spectral perturbation (ERSP) using EEGLAB [39]. The raw signals were visualized as channels through the time representation for the representative subject named sub8. The scalp plot can be visualized to choose a specific channel and time epoch for a selected subject. The ERSP plot requires the installation of the EEGLAB toolbox. After installing the EEGLAB toolbox, users can load the *.vhdr* file to EEGLAB and visualize the ERSP pattern that follows the description.

## Data Validation

### Methods

The technical signal validation was conducted using a BBCI toolbox [38] in the MATLAB 2019a environment. The initial settings of the recording program for converting an analog signal into a digital one were slightly different for each signal because of the scale of the signal amplitude. For each of the EEG, EMG, and EOG signals, triggers were marked to indicate the experimental state.

Initially, for data pre-processing, we applied a zero-phase $4^{th}$ Butterworth filter for band-pass signal filtering in the EEG signals. The data were filtered between 8 and 30 Hz (mu- and beta-bands, respectively) known as within motor-related frequency range and it could also include the spectral range for SMR observation (i.e., [13-15] Hz) [40, 41]. For artifact rejection, the apparent eye-blinking contamination in the EEG signal was removed via independent component analysis (ICA) [42]. To obtain corrected EEG data, we removed the contamination factors using the infomax ICA [43], which is used to decompose brain signals into statistically independent components (ICs). From various types of ICA methods, we adopted the ICA with the infomax algorithm as it could robustly remove artifacts, such as eye and head movement artifacts from the EEG data robustly [44]. The EEG data were transformed by the ICA mixing matrix. The contaminated ICs with patterns similar to the EOG channels (i.e., horizontal and vertical eye movements) were removed. Subsequently, the remaining ICs were projected back into the scalp channel space to be reconstructed as the corrected EEG data (see Fig. 5). Before feature extraction, we segmented a time interval of -0.5 to 4 s for performing EEG classification and also selected a baseline period as -0.5 to 0 s [25].

For evaluating the classification performances using EEG signals, we adopted the common spatial pattern (CSP) algorithm as a feature extraction method and a regularized lin-

Figure/GIGA_Figure5.pdf

**Figure 5.** Infomax ICA based on EOG data was applied to eliminate the eye-movement-induced noise from the original EEG data. The right side shows the corrected EEG data wherein noise has been eliminated by the application of the infomax ICA to the original EEG data.

ear discriminant analysis (RLDA) method as the classification method. CSP feature extraction method and RLDA classifier are generally used as baseline algorithms for decoding EEG-based MI and motor execution in the field of BCI [26, 45, 46]. Especially, CSP, one of the EEG feature extraction methods, is proved to be robust in spatial feature extraction for decoding movement-related tasks and MI. CSP trained for finding the optimal spatial filter that maximizes inter-class variation and minimizes intra-class variation. We calculated a transformation matrix using CSP consisting of the logarithmic variances of the first and last three columns were used as a feature. We trained the RLDA classifier by adding a regularization term to the covariance matrix using the optimal shrinkage parameters [47, 48]. Essentially, shrinkage performs regularization to improve the estimation of covariance matrices where the training samples are fewer than features. Therefore, during the training period, the optimal shrinkage parameter was automatically estimated with the maximum covariance between classes. In the classification procedure, we classified the multi-class according to each different experimental task, separately, such as arm-reaching along six directions (6-class), hand-grasping of three objects (3-class), and wrist-twisting with two different motions (2-class), as depicted in Fig. 3. We applied $10\times10$-fold cross-validation for fair performance measurement so that we partitioned the data samples as equal sizes into 10 subsets. One of the subsets was selected as the test dataset and the remaining others as the training datasets. The cross-validation process was conducted 10 times with each of the 10 subsets used once as the test dataset to avoid variability problems in performance evaluation. The evaluation was estimated using all recorded classes simultaneously over all the recording sessions. Table 2 presents the averaged evaluation results obtained by estimating the classification performance of each arm-reaching (6-class), hand-grasping (3-class), and wrist-

Figure/GIGA_Figure6.pdf

**Figure 6.** Example representation of EMG activation according to each 7-channel EMG. From the top, the plot represents the activities of the EMG signals as the representative subject named sub4 performs arm-reaching, hand-grasping, and wrist-twisting tasks, respectively.

Figure/GIGA_Figure7.pdf

**Figure 7.** EEG data validation via spectral representation. The event-related desynchronization/synchronization (ERD/ERS) representation of channel C3 during multiple recording sessions.

twisting (2-class) task.

Furthermore, we checked the EMG activation to verify whether the upper-extremity movement or not according to the tasks. In this work, the EMG signals were pre-processed from 10–500 Hz with a Butterworth $5^{th}$ zero-phase bandpass filter [34, 49]. We segmented a time interval of -0.5 to -4 s for EMG data analysis. We selected the interval of -0.5 to 0 s for as the baseline period. Subsequently, the data were rectified using the absolute values, following which we calculated the moving average of EMG amplitudes with a 100 ms interval. The EMG signals could show how well the subjects had followed the experimental protocol for each task. For example, if the subject were asked to perform the MI task, the EMG activation should not show a peak shape because of the static state at that time, as depicted in Fig. 6. EMG signal patterns contains important information regarding muscle activation and noise. For example, the noise due to heart beat and other unrelated movements reduces the quality of EMG signals. While the subjects performed the wrist-twisting task, the amplitude scale was reduced as compared with other work, so that noise information could be confirmed to be displayed. Meanwhile, in the real-movement tasks, the EMG signals could show the activation while the subjects were performing the upper-extremity movement task. Notably, the signal amplitude of channel $EMG_6$ is higher than those of the other channels. Since biceps are relatively bigger than other muscles, a peak phenomenon occurred [50]. Particularly, biceps are used the most in bending the arm. The EMG signals featured a large signal amplitude, as more muscle activity increases the signal amplitude. Consequently, the EMG signal amplitude was large for the arm-reaching and hand-grasping tasks, but small for the wrist-twisting task [51].

## Results and Discussion

We verified the data through the EEG-based classification results, the EMG signal quality, and spectral and spatial EEG presentations. First, we verified the data on the basis of EMG signal quality, as shown in Fig. 6. Using the EMG signals recorded while the subjects performed each arm-reaching, hand-grasping, and wrist-twisting experiment, we could confirm the quality of the data obtained. Furthermore, dynamic EMG signals were observed during the real-movement sessions; additionally insignificant changes were observed in the EMG signals close to the rest state in the MI sessions. The EMG signals, as particularly, proved that we had collected the data appropriately, as they were not activated throughout the entire duration of the actual motion session, and appeared strongly for the actual movements of the subjects right after approximately 0.5 seconds from the onset. Additionally, the non-activated EMG signals during the MI tasks clearly indicate that the movement artifacts of the corresponding EEG signal were minimized.

We also analyzed the EEG signals in the spectral and spatial domains to confirm the data quality. Fig. 7 shows the examples of spectral energy information in the EEG data for a representative subject during multiple recording sessions. The ERSP plot illustrates spectral variability according to the time epoch in a certain channel (C3). Generally, the ERSP plot showed ERD/ERS patterns, which reflect sensorimotor activation and deactivation, respectively. The ERD patterns can be seen during motor preparation, execution, and imagery as correlations in an activated cortical region. ERS can be observed after the imagery or execution of movement over the same region [52, 53]. In Fig. 7, ERD/ERS patterns appeared during all the imagery phases (0 to 4 s), showing the same cortical activation on the mu-band across all the recording sessions. Additionally, we conducted a statistical analysis to confirm whether the subjects consistently performed MI via their single upper-extremities as instructed at the beginning. We selected typical EEG channels on the motor cortex corresponding to right-hand (channel C3), left-hand (channel C4), and foot (channel Cz) imageries [54, 55]. The mean ERD/ERS values for channel C3 contain significant differences compared with other channels (Cz and C4), as confirmed through the paired $t$-test. All the $p$-values between the channels were below 0.05 except for a few subjects.

Fig. 8(a) depicts the representative spatial distribution obtained using grand-average signal amplitude responses per time period [56, 57]. For subject sub2, the data for wrist pronation is presented in Fig. 8(a). We used all of EEG channels and adopted signal processing (similar to that in the pre-processing steps), such as band-pass filtering and epoch segmentation, and we also employed a baseline period. We applied the moving average of EEG amplitudes with a 200 ms interval. The topographic maps of the mean amplitudes were visualized in four temporal intervals for all the recording sessions. Therefore, the left-hemisphere of the contralateral sensorimotor region was activated while performing tasks in all the recording sessions. Hence, over time, the mean amplitude in the contralateral sensorimotor region of the left hemisphere significantly increased clearly as the subjects performed MI. Moreover, it was clearly observed that the contralateral sensorimotor regions of the most subjects were activated by MI in the 3 to 4 s. Therefore, we confirmed 0 to 2 s as the preparation

Figure/GIGA_Figure8.pdf

**Figure 8.** Data validation performed using the spatial information with respect to scalp activation. (a) Scalp topography of the wrist pronation task for a representative subject named sub2. (b) Source imaging analysis performed via sLORETA using by a statistically significant difference in the MI tasks for a representative subject named sub13 (red: $p<0.05$, yellow: $p<0.01$).

period and that the contralateral sensorimotor region was appropriately activated after the first 2 s in the recording phases.

For a more sophisticated analysis, we used a source imaging technique to identify and compare activated regions in the brain with each other while a subject was performing each task. We used a standardized low-resolution electromagnetic tomography (sLORETA)-based current density estimation technique for inverse modeling the brain points that were activated from the EEG signals. sLORETA is a variant of the weighted minimum norm estimation technique for obtaining an inverse solution [31, 58]. We visualized the activated regions of the brain for each task and showed them in terms of the horizontal, sagittal, and coronal planes, as shown in Fig. 8(b). The source images were visualized by the significant differences by calculating the $p$-values for the spatial distribution between the baseline period (-0.5 to 0 s) and MI period (0 to 4 s). The yellow colors indicate the $p$-values below 0.01, and the red colors those below 0.05 [59]. The main differences were observed in the supplementary motor region and premotor cortex; they indicate that the subjects satisfactorily performed MI. Since all the subjects performed MI related to the movement of the right upper limb, it can be confirmed from Fig. 8(b) that the left side region of the cortex associated with the MI is activated.

We evaluated the dataset quality by observing BCI classification performances (see Table 2). By using the baseline machine learning method, we confirmed that the accuracies were at least higher than chance-level accuracy for each class. The performances were validated according to tasks including arm-reaching, hand-grasping, and wrist-twisting. We computed the chance-level accuracies with a significant confidence level ($a$ = 5%) [60] and could obtain the chance results per evaluation as 0.17 (arm-reaching), 0.34 (hand-grasping), and 0.51

**Table 2.** Classification accuracy for each task across all the subjects during multiple recording sessions. The evaluation was estimated from each upper-extremity motions such as arm-reaching (6-class), hand grasping (3-class), wrist-twisting (2-class) in real-movement and MI.

| Subjects | Session I | | | | | | Session II | | | | | | Session III | | | | | |
|---|---|---|---|---|---|---|---|---|---|---|---|---|---|---|---|---|---|---|
| | Real-movement | | | Motor imagery | | | Real-movement | | | Motor imagery | | | Real-movement | | | Motor imagery | | |
| | Reach | Grasp | Twist | Reach | Grasp | Twist | Reach | Grasp | Twist | Reach | Grasp | Twist | Reach | Grasp | Twist | Reach | Grasp | Twist |
| sub1 | 0.21 (±0.01) | 0.40 (±0.02) | 0.61 (±0.03) | 0.21 (±0.02) | 0.42 (±0.04) | 0.60 (±0.04) | 0.21 (±0.02) | 0.41 (±0.03) | 0.56 (±0.03) | 0.21 (±0.01) | 0.42 (±0.04) | 0.58 (±0.03) | 0.24 (±0.01) | 0.43 (±0.03) | 0.58 (±0.03) | 0.21 (±0.02) | 0.40 (±0.03) | 0.57 (±0.03) |
| sub2 | 0.24 (±0.01) | 0.41 (±0.03) | 0.56 (±0.04) | 0.22 (±0.01) | 0.40 (±0.02) | 0.63 (±0.02) | 0.23 (±0.02) | 0.41 (±0.03) | 0.63 (±0.03) | 0.23 (±0.01) | 0.40 (±0.02) | 0.56 (±0.04) | 0.20 (±0.03) | 0.41 (±0.02) | 0.66 (±0.02) | 0.21 (±0.02) | 0.40 (±0.02) | 0.60 (±0.04) |
| sub3 | 0.18 (±0.02) | 0.42 (±0.02) | 0.59 (±0.04) | 0.20 (±0.01) | 0.39 (±0.04) | 0.61 (±0.02) | 0.19 (±0.02) | 0.40 (±0.03) | 0.59 (±0.03) | 0.20 (±0.02) | 0.38 (±0.03) | 0.63 (±0.05) | 0.20 (±0.01) | 0.41 (±0.03) | 0.56 (±0.04) | 0.19 (±0.02) | 0.36 (±0.02) | 0.55 (±0.05) |
| sub4 | 0.17 (±0.02) | 0.40 (±0.05) | 0.64 (±0.03) | 0.21 (±0.02) | 0.39 (±0.03) | 0.60 (±0.05) | 0.28 (±0.02) | 0.39 (±0.01) | 0.59 (±0.03) | 0.18 (±0.02) | 0.38 (±0.03) | 0.59 (±0.03) | 0.21 (±0.01) | 0.41 (±0.02) | 0.57 (±0.05) | 0.20 (±0.02) | 0.40 (±0.02) | 0.54 (±0.03) |
| sub5 | 0.21 (±0.01) | 0.39 (±0.03) | 0.59 (±0.02) | 0.20 (±0.01) | 0.43 (±0.03) | 0.55 (±0.03) | 0.20 (±0.02) | 0.41 (±0.03) | 0.54 (±0.04) | 0.18 (±0.01) | 0.35 (±0.03) | 0.56 (±0.02) | 0.19 (±0.02) | 0.41 (±0.02) | 0.55 (±0.06) | 0.22 (±0.01) | 0.35 (±0.03) | 0.58 (±0.02) |
| sub6 | 0.19 (±0.02) | 0.41 (±0.03) | 0.60 (±0.04) | 0.19 (±0.02) | 0.46 (±0.04) | 0.55 (±0.04) | 0.22 (±0.02) | 0.43 (±0.04) | 0.60 (±0.03) | 0.24 (±0.01) | 0.44 (±0.03) | 0.60 (±0.03) | 0.24 (±0.02) | 0.47 (±0.03) | 0.62 (±0.05) | 0.21 (±0.01) | 0.46 (±0.02) | 0.54 (±0.03) |
| sub7 | 0.22 (±0.01) | 0.38 (±0.03) | 0.61 (±0.03) | 0.24 (±0.02) | 0.53 (±0.03) | 0.63 (±0.03) | 0.21 (±0.02) | 0.53 (±0.03) | 0.60 (±0.02) | 0.27 (±0.02) | 0.60 (±0.02) | 0.63 (±0.03) | 0.21 (±0.02) | 0.65 (±0.03) | 0.57 (±0.04) | 0.34 (±0.02) | 0.53 (±0.03) | 0.59 (±0.03) |
| sub8 | 0.20 (±0.01) | 0.50 (±0.03) | 0.59 (±0.03) | 0.21 (±0.02) | 0.54 (±0.03) | 0.65 (±0.04) | 0.22 (±0.02) | 0.66 (±0.03) | 0.53 (±0.03) | 0.19 (±0.02) | 0.79 (±0.03) | 0.63 (±0.04) | 0.22 (±0.02) | 0.65 (±0.02) | 0.61 (±0.05) | 0.19 (±0.01) | 0.75 (±0.02) | 0.59 (±0.04) |
| sub9 | 0.20 (±0.01) | 0.38 (±0.04) | 0.57 (±0.02) | 0.25 (±0.01) | 0.35 (±0.03) | 0.56 (±0.02) | 0.21 (±0.02) | 0.38 (±0.03) | 0.62 (±0.05) | 0.21 (±0.02) | 0.39 (±0.03) | 0.60 (±0.04) | 0.20 (±0.02) | 0.41 (±0.03) | 0.58 (±0.04) | 0.19 (±0.01) | 0.39 (±0.05) | 0.59 (±0.05) |
| sub10 | 0.20 (±0.02) | 0.43 (±0.02) | 0.58 (±0.05) | 0.21 (±0.02) | 0.41 (±0.02) | 0.55 (±0.02) | 0.22 (±0.01) | 0.51 (±0.04) | 0.60 (±0.04) | 0.18 (±0.02) | 0.48 (±0.04) | 0.60 (±0.03) | 0.22 (±0.01) | 0.39 (±0.03) | 0.58 (±0.05) | 0.19 (±0.02) | 0.37 (±0.02) | 0.62 (±0.03) |
| sub11 | 0.25 (±0.02) | 0.43 (±0.02) | 0.58 (±0.05) | 0.35 (±0.01) | 0.41 (±0.03) | 0.62 (±0.03) | 0.24 (±0.02) | 0.52 (±0.03) | 0.58 (±0.04) | 0.26 (±0.02) | 0.40 (±0.02) | 0.64 (±0.02) | 0.27 (±0.02) | 0.64 (±0.02) | 0.58 (±0.05) | 0.33 (±0.02) | 0.59 (±0.02) | 0.56 (±0.02) |
| sub12 | 0.21 (±0.02) | 0.77 (±0.02) | 0.56 (±0.02) | 0.21 (±0.01) | 0.93 (±0.02) | 0.55 (±0.04) | 0.22 (±0.01) | 0.89 (±0.02) | 0.56 (±0.03) | 0.21 (±0.01) | 0.94 (±0.02) | 0.63 (±0.03) | 0.21 (±0.02) | 0.58 (±0.02) | 0.58 (±0.04) | 0.21 (±0.02) | 0.56 (±0.02) | 0.65 (±0.03) |
| sub13 | 0.20 (±0.01) | 0.40 (±0.03) | 0.58 (±0.06) | 0.19 (±0.02) | 0.44 (±0.04) | 0.65 (±0.03) | 0.22 (±0.02) | 0.37 (±0.02) | 0.61 (±0.04) | 0.21 (±0.02) | 0.35 (±0.03) | 0.59 (±0.03) | 0.19 (±0.02) | 0.43 (±0.02) | 0.56 (±0.04) | 0.17 (±0.02) | 0.37 (±0.04) | 0.57 (±0.03) |
| sub14 | 0.22 (±0.02) | 0.43 (±0.03) | 0.52 (±0.01) | 0.21 (±0.02) | 0.46 (±0.03) | 0.56 (±0.05) | 0.23 (±0.02) | 0.69 (±0.02) | 0.54 (±0.02) | 0.21 (±0.02) | 0.42 (±0.03) | 0.55 (±0.03) | 0.21 (±0.01) | 0.50 (±0.02) | 0.56 (±0.02) | 0.18 (±0.02) | 0.45 (±0.02) | 0.54 (±0.03) |
| sub15 | 0.21 (±0.01) | 0.38 (±0.04) | 0.61 (±0.03) | 0.19 (±0.02) | 0.40 (±0.04) | 0.58 (±0.04) | 0.21 (±0.02) | 0.51 (±0.02) | 0.63 (±0.03) | 0.18 (±0.01) | 0.43 (±0.03) | 0.57 (±0.05) | 0.20 (±0.02) | 0.46 (±0.03) | 0.61 (±0.04) | 0.20 (±0.02) | 0.41 (±0.02) | 0.63 (±0.04) |
| sub16 | 0.21 (±0.02) | 0.40 (±0.03) | 0.63 (±0.04) | 0.23 (±0.02) | 0.43 (±0.02) | 0.59 (±0.03) | 0.20 (±0.01) | 0.43 (±0.04) | 0.59 (±0.03) | 0.20 (±0.02) | 0.36 (±0.03) | 0.51 (±0.03) | 0.19 (±0.03) | 0.43 (±0.03) | 0.55 (±0.04) | 0.20 (±0.02) | 0.37 (±0.04) | 0.65 (±0.02) |
| sub17 | 0.25 (±0.02) | 0.86 (±0.03) | 0.56 (±0.04) | 0.20 (±0.01) | 0.34 (±0.03) | 0.59 (±0.04) | 0.23 (±0.01) | 0.86 (±0.03) | 0.65 (±0.03) | 0.21 (±0.01) | 0.36 (±0.03) | 0.62 (±0.03) | 0.27 (±0.01) | 0.41 (±0.03) | 0.56 (±0.04) | 0.17 (±0.02) | 0.39 (±0.04) | 0.52 (±0.04) |
| sub18 | 0.22 (±0.01) | 0.47 (±0.02) | 0.64 (±0.03) | 0.22 (±0.01) | 0.36 (±0.02) | 0.63 (±0.05) | 0.21 (±0.01) | 0.70 (±0.03) | 0.64 (±0.03) | 0.22 (±0.02) | 0.50 (±0.03) | 0.63 (±0.04) | 0.24 (±0.02) | 0.52 (±0.03) | 0.58 (±0.03) | 0.20 (±0.02) | 0.37 (±0.01) | 0.57 (±0.02) |
| sub19 | 0.22 (±0.02) | 0.72 (±0.02) | 0.61 (±0.03) | 0.22 (±0.02) | 0.39 (±0.03) | 0.62 (±0.03) | 0.22 (±0.02) | 0.66 (±0.02) | 0.50 (±0.03) | 0.18 (±0.01) | 0.40 (±0.03) | 0.61 (±0.02) | 0.19 (±0.02) | 0.77 (±0.03) | 0.64 (±0.03) | 0.21 (±0.02) | 0.40 (±0.02) | 0.54 (±0.03) |
| sub20 | 0.43 (±0.01) | 0.38 (±0.02) | 0.56 (±0.04) | 0.19 (±0.02) | 0.41 (±0. 03) | 0.58 (±0.03) | 0.35 (±0.02) | 0.60 (±0.03) | 0.55 (±0.03) | 0.21 (±0.01) | 0.41 (±0.02) | 0.59 (±0.04) | 0.29 (±0.02) | 0.42 (±0.03) | 0.61 (±0.03) | 0.20 (±0.02) | 0.40 (±0.02) | 0.54 (±0.04) |
| sub21 | 0.28 (±0.02) | 0.45 (±0.04) | 0.55 (±0.05) | 0.20 (±0.02) | 0.43 (±0.02) | 0.60 (±0.02) | 0.22 (±0.02) | 0.45 (±0.04) | 0.56 (±0.03) | 0.21 (±0.02) | 0.46 (±0.03) | 0.53 (±0.02) | 0.29 (±0.02) | 0.53 (±0.02) | 0.58 (±0.04) | 0.19 (±0.02) | 0.40 (±0.01) | 0.58 (±0.02) |
| sub22 | 0.29 (±0.02) | 0.73 (±0.02) | 0.61 (±0.05) | 0.23 (±0.02) | 0.46 (±0.03) | 0.60 (±0.03) | 0.24 (±0.02) | 0.46 (±0.02) | 0.55 (±0.03) | 0.21 (±0.02) | 0.36 (±0.03) | 0.61 (±0.03) | 0.23 (±0.01) | 0.60 (±0.03) | 0.55 (±0.03) | 0.21 (±0.02) | 0.42 (±0.02) | 0.55 (±0.03) |
| sub23 | 0.19 (±0.01) | 0.45 (±0.03) | 0.60 (±0.04) | 0.21 (±0.02) | 0.42 (±0.03) | 0.57 (±0.04) | 0.25 (±0.01) | 0.36 (±0.03) | 0.60 (±0.04) | 0.20 (±0.02) | 0.38 (±0.02) | 0.57 (±0.04) | 0.22 (±0.01) | 0.52 (±0.03) | 0.60 (±0.03) | 0.21 (±0.01) | 0.36 (±0.03) | 0.57 (±0.03) |
| sub24 | 0.20 (±0.01) | 0.35 (±0.03) | 0.53 (±0.02) | 0.23 (±0.01) | 0.43 (±0.03) | 0.61 (±0.04) | 0.21 (±0.01) | 0.47 (±0.04) | 0.59 (±0.02) | 0.20 (±0.02) | 0.47 (±0.03) | 0.58 (±0.03) | 0.22 (±0.01) | 0.57 (±0.03) | 0.51 (±0.04) | 0.22 (±0.03) | 0.35 (±0.03) | 0.56 (±0.01) |
| sub25 | 0.24 (±0.01) | 0.49 (±0.04) | 0.57 (±0.04) | 0.19 (±0.01) | 0.41 (±0.04) | 0.61 (±0.04) | 0.22 (±0.02) | 0.64 (±0.03) | 0.57 (±0.04) | 0.20 (±0.01) | 0.41 (±0.02) | 0.58 (±0.04) | 0.24 (±0.01) | 0.58 (±0.03) | 0.60 (±0.04) | 0.21 (±0.01) | 0.42 (±0.03) | 0.60 (±0.03) |
| Average | 0.23 (±0.05) | 0.47 (±0.14) | 0.59 (±0.03) | 0.22 (±0.03) | 0.44 (±0.11) | 0.60 (±0.03) | 0.23 (±0.03) | 0.53 (±0.15) | 0.58 (±0.04) | 0.21 (±0.02) | 0.45 (±0.14) | 0.59 (±0.03) | 0.22 (±0.03) | 0.50 (±0.10) | 0.58 (±0.03) | 0.21 (±0.04) | 0.43 (±0.09) | 0.58 (±0.03) |

(wrist-twisting). Table 2 represents the classification accuracies with the standard deviation for each subject and recording session. Because our dataset was recorded over three different sessions, it allows for further research related to BCI-calibration problems. According to our classification result obtained using the baseline decoding method and conventional approach, some subjects showed a significant change in classification results when the session as changed; however, the other subjects showed similar classification accuracies over different sessions.

The decoding of intuitive upper-extremity movements from EEG signals is a challenging study. However, if the upper-extremity movements can be successfully analyzed, the BCI technology could be applied to many applications. The BCI may be applied to the operation of robotic instruments, such as a robotic arm and neuro-prosthesis related to upper-extremity movements, or to control peripheral devices using commands based on decoding the movement intention. In this study, for more advances, we provide a multimodal signal dataset when the subjects executed and imagined the intuitive move-

ment tasks using a single arm. As mentioned in "*Experimental paradigm*", decoding various tasks from the same limb could provide various BCI classes and significantly intuitive communication between users and BCI systems as compared with a typical paradigm. Therefore, a robust decoding model for this dataset could contribute one step toward the advancement of a practical and commercial BCI. Therefore, one must obtain high-quality data. Additionally, more advanced analyses can be attempted because we have constructed a database that includes not only the EOG and EMG data but also the EEG data. For example, the EOG data might be used to remove the noise due to explicit eye movement from the EEG data. Additionally, the EMG data may demonstrate the integrity of the EEG data by showing that no movement-related interference was present in the EMG data in the analysis and MI tasks associated with the EEG. In this work, we recorded the signals' data using three modalities, namely, EEG, EOG, and EMG. Additionally, we collected data from 25 subjects and divided the experiment into three sessions to prepare the dataset. We also provided the spectral representation and time-spatial distribution of a representative subject according to multiple recording sessions. Generally, we confirmed that the data variability among each recording session did not show any significant differences in our dataset (i.e., $p<0.05$). Furthermore, we confirmed that the classification accuracy per task was slightly higher than the chance-level accuracy using the baseline method. Conversely, despite the difficult tasks involved in the experiments, the subjects successfully focused on the experiments so that we could obtain high-quality data. In the future, the users of this dataset can contribute to increasing the present classification accuracy using their novel methodology.

Through preliminary data validation, we determined the sufficient quality of our dataset as sufficient. Further studies can be performed to determine the hidden characteristics and features related to the intention of upper-limb movements using only our EEG data, while the EOG data can be used to filter noise for obtaining clear EEG signals. Simultaneously, researchers can attempt to combine EEG and EMG signals using our dataset for developing hybrid BCI systems. In related studies, the hybrid approaches showed remarkable possibility to improve the decoding performances of real-movement and MI-based BCIs [34, 61]. Additionally, our dataset can be used for studies that analyze the correlations between EEG and EMG. In related studies, the relevance of EEG and EMG signals can be found through the connectivity analysis of the data acquired over a specific period. For example, a statistical analysis of activated EEG channels conducted during the activation of a particular EMG channel can determine the region of the brain, channel location, and frequency band directly related to the movement of the particular muscle [62].

Inter-session comparisons are also important topics in BCI experiments. Since the BCI systems are recalibrated at the beginning of each recording session, this procedure becomes time-consuming and thus may limit the adoption of BCI systems for long-term daily usage [63]. Furthermore, we recorded data over three sessions to enable cross-session analysis. For each session, we collected a dataset of uniform quality on the basis of classification results (see Table 2), as we focused on conducting all the experiments under stable conditions. Researchers can analyze the decoding performance using our dataset from the entire session and they can also compare the decoding results of each session with each other. Different approaches are also available, and they include training the decoding model in a particular session and testing the model using data from independent sessions on the basis of the principle of transfer learning in BCI, as done in [64]. Accordingly, creating a session-independent BCI decoding model is critical to establishing a practical BCI system such as biometric authentication system [65] and brain-controlled AR/VR system [66]. Therefore, our experimental data can be useful for studies to build session-independent decoding models.

## Availability of supporting data and materials

The data supporting this paper, including EEG, EMG, and EOG datasets and example codes, are available in the GigaScience database, GigaDB repository [67].

### Abbreviations

BCI: brain-computer interface; EEG: electroencephalography; fNIRS: functional near-infrared spectroscopy; MI: motor imagery; SSVEP: steady-state visual evoked potential; ERP: event-related potential; MRCP:movement-related cortical potential; EMG: electromyography; EOG: electrooculography; LCD: liquid-crystal display; SMR: somatosensory rhythm; ERSP: event-related spectral perturbation; ICA: independent component analysis; ICs: independent components; CSP: common spatial pattern; RLDA: regularized linear discriminant analysis; ERD/ERS: event-related desynchronization/synchronization; sLORETA: standardized low-resolution electromagnetic tomography

### Ethical Approval

This study was reviewed and approved by the Institutional Review Board at Korea University (1040548-KU-IRB-17-181-A-2).

### Competing Interests

The authors declare no competing interests.

### Funding

This work was partly supported by Institute of Information & Communications Technology Planning & Evaluation (IITP) grant funded by the Korea government (No. 2015-0-00185, Development of Intelligent Pattern Recognition Softwares for Ambulatory Brain-Computer Interface; No. 2017-0-00451, Development of BCI based Brain and Cognitive Computing Technology for Recognizing User's Intentions using Deep Learning; No. 2019-0-00079, Department of Artificial Intelligence, Korea University).

### Author's Contributions

J.-H. Jeong, J.-H. Cho, and K.-H. Shim designed the experimental protocols and paradigms. J.-H. Jeong, B.-H. Kwon, B.-H. Lee, D.-Y. Lee, and D.-H. Lee collected the data and checked the physical and mental states of the participant during the experiments. J.-H Jeong, J.-H. Cho, and S.-W. Lee revised the manuscript. All the authors analyzed and validated the collected data technically. Furthermore, the authors prepared the manuscript and approved the database public.

## References

1. Kaufmann T, Kübler A. Beyond maximum speed—a novel two-stimulus paradigm for brain-computer interfaces based on event-related potentials (P300-BCI). J Neural Eng 2014;11(5):056004.

2. Kim KT, Suk HI, Lee SW. Commanding a brain-controlled wheelchair using steady-state somatosensory evoked potentials. IEEE Trans Neural Syst Rehabil Eng 2018;26(3):654–665.
3. Jeong JH, Shim KH, Kim DJ, et al. Trajectory decoding of arm reaching movement imageries for brain-controlled robot arm system. In: Proc. 41th Int. Conf. IEEE Eng. Med. Biol. Soc. (EMBC) Berlin, Germany; July 23 2019. p. 5544–5547.
4. Penaloza CI, Nishio S. BMI control of a third arm for multitasking. Sci Robot 2018;3(20):eaat1228.
5. Meng J, Zhang S, Bekyo A, et al. Noninvasive electroencephalogram based control of a robotic arm for reach and grasp tasks. Sci Rep 2016;6:38565.
6. Kwak NS, Müller KR, Lee SW. A lower limb exoskeleton control system based on steady state visual evoked potentials. J Neural Eng 2015;12(5):056009.
7. He Y, Eguren D, Azorín JM, et al. Brain–machine interfaces for controlling lower-limb powered robotic systems. J Neural Eng 2018;15(2):021004.
8. Abiri R, Borhani S, Sellers EW, et al. A comprehensive review of EEG-based brain–computer interface paradigms. J Neural Eng 2019;16(1):011001.
9. Wolpaw JR, Birbaumer N, McFarland DJ, et al. Brain–computer interfaces for communication and control. Clin Neurophysiol 2002;113(6):767–791.
10. Craik A, He Y, Contreras-Vidal JLP. Deep learning for Electroencephalogram (EEG) classification tasks: A review. J Neural Eng 2019;16(3):031001.
11. Kakkos I, Miloulis ST, Gkiatis K, et al. Human–machine interfaces for motor rehabilitation. Adv Comput Intell in Healthcare-7 2020;p. 1–16.
12. Lee MH, Fazli S, Mehnert J, et al. Subject-dependent classification for robust idle state detection using multi-modal neuroimaging and data-fusion techniques in BCI. Patt Recognit 2015;48(8):2725–2737.
13. Ang KK, Guan C. EEG-based strategies to detect motor imagery for control and rehabilitation. IEEE Trans Neural Syst Rehabil Eng 2017;25(4):392–401.
14. Tabar YR, Halici U. A novel deep learning approach for classification of EEG motor imagery signals. J Neural Eng 2016;14(1):016003.
15. Lu N, Li T, Ren X, et al. A deep learning scheme for motor imagery classification based on restricted Boltzmann machines. IEEE Trans Neural Syst Rehabil Eng 2017;25(6):566–576.
16. Won DO, Hwang HJ, Dähne S, et al. Effect of higher frequency on the classification of steady-state visual evoked potentials. J Neural Eng 2015;13(1):016014.
17. Kwak NS, Müller KR, Lee SW. A convolutional neural network for steady state visual evoked potential classification under ambulatory environment. PloS One 2017;12(2):e0172578.
18. Yeom SK, Fazli S, Müller KR, et al. An efficient ERP-based brain-computer interface using random set presentation and face familiarity. PloS One 2014;9(11).
19. Shakeel A, Navid MS, Anwar MN, et al. A review of techniques for detection of movement intention using movement-related cortical potentials. Comput Math Method M 2015;2015.
20. Jeong JH, Kwak NS, Guan C, et al. Decoding movement-related cortical potentials based on subject-dependent and section-wise spectral filtering. IEEE Trans Neural Syst Rehabil Eng 2020;28(3):687–698.
21. Kaya M, Binli MK, Ozbay E, et al. A large electroencephalographic motor imagery dataset for electroencephalographic brain computer interfaces. Sci Data 2018;5:180211.
22. Cao Z, Chuang CH, King JK, et al. Multi-channel EEG recordings during a sustained-attention driving task. Sci Data 2019;6(1):1–8.
23. Lee MH, Kwon OY, Kim YJ, et al. EEG dataset and OpenBMI toolbox for three BCI paradigms: an investigation into BCI illiteracy. GigaScience 2019;8(5):giz002.
24. Choi GY, Han CH, Jung YJ, et al. A multi-day and multi-band dataset for a steady-state visual-evoked potential–based brain-computer interface. GigaScience 2019;8(11):giz133.
25. Cho H, Ahn M, Ahn S, et al. EEG datasets for motor imagery brain–computer interface. GigaScience 2017;6(7):gix034.
26. Shiman F, López-Larraz E, Sarasola-Sanz A, et al. Classification of different reaching movements from the same limb using EEG. J Neural Eng 2017;14(4):046018.
27. Ofner P, Schwarz A, Pereira J, et al. Upper limb movements can be decoded from the time-domain of low-frequency EEG. PloS One 2017;12(8).
28. Hossain I, Khosravi A, Hettiarachchi I, et al. Batch mode query by committee for motor imagery-based BCI. IEEE Trans Neural Syst Rehabil Eng 2019;27(1):13–21.
29. Kwon OY, Lee MH, Guan C, Lee SW. Subject-independent brain–computer interfaces based on deep convolutional neural networks. IEEE Trans Neural Netw Learn Syst 2019;p. 1–14.
30. Lee BH, Jeong JH, Lee SW. SessionNet: Feature similarity-based weighted ensemble learning for motor imagery classification. IEEE Access 2020;8:134524–134535.
31. Edelman BJ, Baxter B, He B. EEG source imaging enhances the decoding of complex right-hand motor imagery tasks. IEEE Trans Biomed Eng 2015;63(1):4–14.
32. Leske S, Dalal SS. Reducing power line noise in EEG and MEG data via spectrum interpolation. NeuroImage 2019;189:763–776.
33. Jeong JH, Shim KH, Kim DJ, et al. Brain-controlled robotic arm System based on multi-directional CNN-BiLSTM network using EEG signals. IEEE Trans Neural Syst Rehabil Eng 2020;28(5):1226–1238.
34. Li X, Samuel OW, Zhang X, et al. A motion-classification strategy based on sEMG-EEG signal combination for upper-limb amputees. J Neuroeng Rehabil 2017;14(1):2.
35. Furui A, Hayashi H, Nakamura G, et al. An artificial EMG generation model based on signal-dependent noise and related application to motion classification. PloS One 2017;12(6).
36. Hussain J, Sundaraj K, Subramaniam ID, et al. Muscle fatigue in the three heads of triceps brachii during intensity and speed variations of triceps push-down exercise. Front Physiol 2020;11(112):1–13.
37. Ma J, Zhang Y, Cichocki A, et al. A novel EOG/EEG hybrid human–machine interface adopting eye movements and ERPs: Application to robot control. IEEE Trans Biomed Eng 2014;62(3):876–889.
38. Blankertz B, Tangermann M, Vidaurre C, et al. The Berlin brain–computer interface: non-medical uses of BCI technology. Front Neurosci 2010;4:198.
39. Delorme A, Makeig S. EEGLAB: an open source toolbox for analysis of single-trial EEG dynamics including independent component analysis. J Neurosci Methods 2004;134(1):9–21.
40. Pfurtscheller G, Neuper C. Motor imagery and direct brain-computer communication. Proc IEEE 2001;89(7):1123–1134.
41. Sreeja S, Rabha J, Samanta D, et al. Classification of motor imagery based EEG signals using sparsity approach. In: 9th Int. Conf. Intell. Hum. Com. Interact. (IHCI) Paris, France; Dec. 2017. p. 47–59.
42. Singh B, Wagatsuma H. A removal of eye movement and

blink artifacts from EEG data using morphological component analysis. Comput Math Method M 2017;2017.
43. Bell AJ, Sejnowski TJ. An information-maximization approach to blind separation and blind deconvolution. Neural Comput 1995;7(6):1129–1159.
44. Kachenoura A, Albera L, Senhadji L, et al. ICA: a potential tool for BCI systems. IEEE Signal Process Mag 2007;25(1):57–68.
45. Blankertz B, Tomioka R, Lemm S, et al. Optimizing spatial filters for robust EEG single-trial analysis. IEEE Signal Process Mag 2007;25(1):41–56.
46. Rozado D, Duenser A, Howell B. Improving the performance of an EEG-based motor imagery brain computer interface using task evoked changes in pupil diameter. PloS One 2015;10(3):e0121262.
47. Treder MS, Porbadnigk AK, Avarvand FS, et al. The LDA beamformer: optimal estimation of ERP source time series using linear discriminant analysis. Neuroimage 2016;129:279–291.
48. Mkhadri A. Shrinkage parameter for the modified linear discriminant analysis. Pattern Recognit Lett 1995;16(3):267–275.
49. Trigili E, Grazi L, Crea S, et al. Detection of movement onset using EMG signals for upper-limb exoskeletons in reaching tasks. J Neuroeng Rehabil 2019;16(1):45.
50. Winiarski D. The use of EMG signal in human-machine interface. Automatyka/automatics 2015;19(2):47–61.
51. Wang J, Tang L, Bronlund JE. Surface EMG signal amplification and filtering. Int J Comput Appl 2013;82(1):15–22.
52. Yao L, Mrachacz-Kersting N, Sheng X, et al. A multi-class BCI based on somatosensory imagery. IEEE Trans Neural Syst Rehabil Eng 2018;26(8):1508–1515.
53. Tang N, Guan C, Ang K, et al. Motor imagery-assisted brain-computer interface for gait retraining in neurorehabilitation in chronic stroke. Ann Phys Rehabil Med 2018;61:e188.
54. Kaiser V, Kreilinger A, Müller-Putz GR, et al. First steps toward a motor imagery based stroke BCI: new strategy to set up a classifier. Front Neurosci 2011;5:86.
55. Müller-Putz G, Scherer R, Pfurtscheller G, et al. Temporal coding of brain patterns for direct limb control in humans. Front Neurosci 2010;4:34.
56. Blankertz B, Acqualagna L, Dähne S, et al. The Berlin brain-computer interface: progress beyond communication and control. Front Neurosci 2016;10:530.
57. Amin HU, Malik AS, Kamel N, et al. P300 correlates with learning memory abilities and fluid intelligence. J Neuroeng Rehabil 2015;12(1):87.
58. Handiru VS, Vinod A, Guan C. EEG source space analysis of the supervised factor analytic approach for the classification of multi-directional arm movement. J Neural Eng 2017;14(4):046008.
59. Canuet L, Ishii R, Pascual-Marqui RD, et al. Resting-state EEG source localization and functional connectivity in schizophrenia-like psychosis of epilepsy. PloS One 2011;6(11):e27863.
60. Müller-Putz G, Scherer R, Brunner C, et al. Better than random: a closer look on BCI results. Int J Bioelectromagn 2008;10(1):52–55.
61. Bakshi K, Pramanik R, Manjunatha M, et al. Upper limb prosthesis control: A hybrid EEG-EMG scheme for motion estimation in transhumeral subjects. In: Proc. 40th Int. Conf. IEEE Eng. Med. Biol. Soc. (EMBC) Hawaii, USA; July 17 2018. p. 2024–2027.
62. Cho JH, Jeong JH, Shim KH, et al. Classification of various grasping tasks based on temporal segmentation method using EEG and EMG signals. In: GBCIC Graz, Austria; Sep. 16 2019. p. 1–6.
63. Abu-Rmileh A, Zakkay E, Shmuelof L, et al. Co-adaptive training improves efficacy of a multi-day EEG-based motor imagery BCI training. Front Hum Neurosci 2019;13:362.
64. Azab AM, Mihaylova L, Ang KK, et al. Weighted transfer learning for improving motor imagery-based brain-computer interface. IEEE Trans Neural Syst Rehabil Eng 2019;27(7):1352–1359.
65. Chen Y, Atnafu AD, Schlattner I, et al. A high-security EEG-based login system with RSVP stimuli and dry electrodes. IEEE Trans Inf Forensics and Secur 2016;11(12):2635–2647.
66. Putze F, Vourvopoulos A, Lécuyer A, et al. Brain-computer interfaces and Augmented/Virtual Reality. Front Hum Neurosci 2020;14.
67. Jeong JH, Cho JH, Shim KH, et al. Supporting data for “Multimodal signal dataset for 11 intuitive movement tasks from single upper extremity during multiple recording sessions”. GigaScience Database 2020;http://dx.doi.org/10.5524/100788.
