# Supplementary material for: Multimodal signal dataset for 11 intuitive movement tasks from single upper extremity during multiple recording sessions
Source: Gigascience. 2020 Oct 7;9(10):giaa098. doi: 10.1093/gigascience/giaa098 (PMC7539536; doi:10.1093/gigascience/giaa098)
Supplement: giaa098_Response_to_Reviewer_Comments_Revision_1 [file giaa098_response_to_reviewer_comments_revision_1.pdf]

Author's Response To Reviewer Comments

Close

When we have used the Gigascience latex template in overleaf, the DOI doesn't represent in the manuscript. Therefore, we added DOI as URL in the BibTeX. Please confirm this issue.

In addition, I cannot upload the latex files as .zip file. Therefore, I uploaded the files separately. Please also confirm this issue too.

Close
