# Supplementary material for: Multimodal signal dataset for 11 intuitive movement tasks from single upper extremity during multiple recording sessions
Source: Gigascience. 2020 Oct 7;9(10):giaa098. doi: 10.1093/gigascience/giaa098 (PMC7539536; doi:10.1093/gigascience/giaa098)
Supplement: giaa098_Reviewer_1_Report_Original_Submission — Georgios Dimitrakopoulos -- 5/6/2020 Reviewed [file giaa098_reviewer_1_report_original_submission.pdf]

Reviewer Report

Title: Multimodal signal dataset for 11 intuitive movement tasks from single upper extremity during multiple recording sessions

Version: Original Submission **Date:** 5/6/2020

Reviewer name: Georgios Dimitrakopoulos

Reviewer Comments to Author:

The paper presents the data taken in an experiment concerning hand movement in real and imaginary motion task. The experiment included 11 different motions collecting in total 3300 trials for each of the 25 subjects. This large dataset will be useful for other research groups to expand knowledge in the field of Neuroscience. The description of the experiment and the data is clear, however there are some comments that need to be addressed.
Major Comments:
Indeed the dataset appears to be efficient to facilitate studies about the brain kinetic patterns both in real as well as imaginary motion. This dataset can be used to train BCI simulations or do off-line analysis about kinetic brain aspects. However, since only the dataset is provided, the frequent use of BCI term (title, abstract etc) might be somewhat misleading since currently there is no interface for real-time communication between mental activities and an application.
Regarding Methods, preprocessing of the EEG could also include baseline removal as was done to EMG and referencing. Additionally, somatosensory rhythm (SMR) is usually between 13 to 15Hz, while mu is similar to alpha (8-12Hz) but mostly found over the motor cortex.
The experimental settings took several hours each day for each participants. Is it possible that muscular and mental fatigue was included as noise in the recordings?
The authors performed a validation analysis via neuro-physiological analysis, however this analysis is at preliminary level and could be enhanced by providing more details in results section, e.g. the findings related to ERDS at Fig 7 and areas in Fig 8 (still I acknowledge the main aim is the data description).
Moreover, in the results section and Fig. 7 different spectral and temporal representations are showed in regard to different sessions. Were any systematic differences observed between sessions?
Regarding classification performance, it is not clear, what were the features? Finally, while Fig. 9 provides all the information, a separate summary of classification results (a table or larger version of the "average" columns) could be better for the reader get an overview. Finally, the accuracies are relatively low, however it is acceptable at this level since the aim is to prove the consistency of the dataset.
Minor comments:
Some proofreading and polishing in the English language is required. In addition, in EMG preprocessing the ~ symbol can change to "to".
Since classification is performed, in the introduction section, a few machine learning review papers about BCI can be mentioned such as:
-Kakkos I., Miloulis ST., Gkiatis K., Dimitrakopoulos G.N., Matsopoulos G.K. (2020). Human-Machine Interfaces for Motor Rehabilitation. In: Maglogiannis I., Brahnam S., Jain L. (eds) Advanced

Computational Intelligence in Healthcare-7. Studies in Computational Intelligence, vol 891. Springer, Berlin, Heidelberg. doi: 10.1007/978-3-662-61114-2_1
-Abiri, R., Borhani, S., Sellers, E. W., Jiang, Y., Zhao, X. (2019). A comprehensive review of EEG-based brain-computer interface paradigms," Journal of Neural Engineering, vol. 16, no. 1, p. 011001, Jan. 2019, doi: 10.1088/1741-2552/aaf12e.

Level of Interest

Please indicate how interesting you found the manuscript: Choose an item.

Quality of Written English

Please indicate the quality of language in the manuscript: Choose an item.

Declaration of Competing Interests

Please complete a declaration of competing interests, considering the following questions:

- Have you in the past five years received reimbursements, fees, funding, or salary from an organisation that may in any way gain or lose financially from the publication of this manuscript, either now or in the future?
- Do you hold any stocks or shares in an organisation that may in any way gain or lose financially from the publication of this manuscript, either now or in the future?
- Do you hold or are you currently applying for any patents relating to the content of the manuscript?
- Have you received reimbursements, fees, funding, or salary from an organization that holds or has applied for patents relating to the content of the manuscript?
- Do you have any other financial competing interests?
- Do you have any non-financial competing interests in relation to this paper?

If you can answer no to all of the above, write 'I declare that I have no competing interests' below. If your reply is yes to any, please give details below.

I declare that I have no competing interests

I agree to the open peer review policy of the journal. I understand that my name will be included on my report to the authors and, if the manuscript is accepted for publication, my named report including any attachments I upload will be posted on the website along with the authors' responses. I agree for my report to be made available under an Open Access Creative Commons CC-BY license (http://creativecommons.org/licenses/by/4.0/). I understand that any comments which I do not wish to

be included in my named report can be included as confidential comments to the editors, which will not be published.

Choose an item.

To further support our reviewers, we have joined with Publons, where you can gain additional credit to further highlight your hard work (see: https://publons.com/journal/530/gigascience). On publication of this paper, your review will be automatically added to Publons, you can then choose whether or not to claim your Publons credit. I understand this statement.

Yes Choose an item.
