# Supplementary material for: Multimodal signal dataset for 11 intuitive movement tasks from single upper extremity during multiple recording sessions
Source: Gigascience. 2020 Oct 7;9(10):giaa098. doi: 10.1093/gigascience/giaa098 (PMC7539536; doi:10.1093/gigascience/giaa098)
Supplement: giaa098_Reviewer_2_Report_Original_Submission — M Mousavi -- 5/16/2020 Reviewed [file giaa098_reviewer_2_report_original_submission.pdf]

Reviewer Report

Title: Multimodal signal dataset for 11 intuitive movement tasks from single upper extremity during multiple recording sessions

Version: Original Submission **Date:** 5/16/2020

Reviewer name: M Mousavi

Reviewer Comments to Author:

The authors provide a dataset consisting of EEG, EMG and EOG recordings from 25 subjects performing real and imaginary movements of the right arm and hand across 3 different sessions. This dataset can provide the community with an opportunity for method evaluations. The number of tasks that each subject performs together with the number of trials for each subject are substantial but the classification accuracy is rather low compared to the existing literature. Overall the paper is well written and covers most of the information explaining and evaluating the provided dataset. However, a detailed comparison with the existing datasets (explaining how the provided dataset is novel/different from the existing ones) is missing.

Here are some comments and suggestions to help further clarify the manuscript:

1) Please explain in more details how the provided dataset is novel/different from the existing open-access BCI datasets.

2) Page 2, paragraph 2: it is unclear what the authors mean by 'intuitive decoding without artificial interaction'. The provided citations do not contain information about this particular terminology. This term is also used in the abstract. Please clarify.

3) Page 2, paragraph 2: This sentence is not clear: "For example, to control a neuro-prosthetic arm using typical paradigms, we temporally matched BCI commands with robotic arm motions (e.g., motor imagery for both hands = grasping motion of robotic hand)." What is temporal matching? Please provide appropriate citations as well.

4) Why did the authors choose the movements of the right arm only? Why not both?

5) Are the subjects all right-handed?

6) What do the authors mean by the following sentence: "however, unsatisfactory decoding performance was a significant problem, predominantly when testing in the background of MI and in a session independent situation"?

7) Page 2, experimental design: How did the authors check for the physical and mental states of the subjects? Was this self-reported by the subjects or did a physician evaluate them prior to participating in the experiment?

8) The monitor was placed about 60 cm away from the subjects. This in my opinion is a very short distance as an average adult's extended arm length is beyond 60 cm. Why was this distance selected? How do the authors ensure comfortable extension of the arm during the experiment?

9) Figure 4: For better clarification, please explicitly mention that the EOG channels were channels numbered 32-35 out of the 64-channel acticap montage.

10) In the experiment, when were the subjects notified so as to perform motor imagery or actual

movement? Was it only after the visual cue?
11) This sentence is missing a verb: "In the motor imagery tasks, the subjects only one of the three grasping motions per trial, randomly."
12) Why did the authors use a notch filter while recording? Given that the data is recorded for offline analysis, this does not seem to be necessary. Also, does this mean that the recorded data is not raw but notch filtered? Please provide the specifications of the notch filter?
13) Why did the authors choose to put the reference electrode of the EMG recording on the elbow? I believe the electrode would be affected by the joint and skin movements during the reach and would not be able to provide a reliable reference.
14) Did the EMG electrodes use the same GND as the EEG and EOG channels?
15) It seems that there is a typo where the authors explain which EOG channels were used for horizontal and vertical eye movements. EOG1 and 4 should be for horizontal and EOG2 and 3 for vertical eye movements. Please revise.
16) What sort of pre-processing makes the .mat files? Are the relevant pre-processing files also provided?
17) Do the authors mean to say 'basic pre-processing steps in' where they say "basis pre-processing step on"?
18) Visualization seems to be channels by time and not time by channel (as depicted in Figure 5). Please revise.
19) What was the ICA pipeline (any channel rejection, re-referencing, etc.)? Which ICA algorithm was used for the decomposition?
20) Please remove the term 'shift' in "zero-phase shift bandpass filter".
21) Figure 6: It seems that the location of the EMG electrodes does not capture the wrist movement very well. Please explain. Some sort of statistical analysis is necessary to compare the time series in the two conditions (real movement and motor imagery) within subject and/or across participants.
22) Page 7, paragraph 1:what is the classification done with respect to? Different movements? How many classes at a time? How is the shrinkage parameter estimated?
23) Please support the following sentences by providing appropriate statistical tests: "The ERD patterns can be seen during motor preparation, execution, and imagery as the correlations in an activated cortical area. ERS can be observed after the imagery or execution of movement over the same area."
24) Figure 7: how were the scalp topographies calculated? Was this averaged across many trials? How many and which ones?
25) Please add similar analysis (Figures 6-9) for all subjects and add them to the data directory or as an appendix to the manuscript.
26) Figure 8: what is the scale of the colored regions? Which subject does this data pertain to?
27) Please update the reported chance levels with a confidence interval depending on the number of available trials. See the following paper: MÃ¼ller-Putz, Gernot, et al. "Better than random: a closer look on BCI results." International Journal of Bioelectromagnetism 10.ARTICLE (2008): 52-55.
28) What is the classification procedure? How are the train and test sets selected?
29) Figure 9: Please provide the same range (0-1) for the y-axis in all plots. Also, what do the bar heights and the error bars represent?
30) Majority of the subjects show classification accuracy very close to chance level. Please explain.

31) The authors mention that "We also analyzed whether the data were inter-session dependent or independent;" where in the manuscript is this explained?
32) Please explain in the discussion section how the rather low classification accuracy and the fact that the experiment only focused on right arm/hand movements may limit the application of the provided dataset.
33) Please explain further the suggested hybrid EEG/EMG BCI using the dataset. If a subject were capable of generating such large EMG by fully executing the arm/hand movements, why would he/she need the BCI?
34) Please cite a few relevant literature that use similar ideas to the ones mentioned in the discussion section, i.e., the connectivity, the hybrid BCI, inter-session comparisons, transfer learning, etc. These are not novel ideas and should be properly cited.
35) What do the authors mean by this sentence in the last paragraph? "The variance of the averaged classification accuracy in session-to-session data was low."
36) Please provide a link to the repository.

Level of Interest

Please indicate how interesting you found the manuscript: Choose an item.

Quality of Written English

Please indicate the quality of language in the manuscript: Choose an item.

Declaration of Competing Interests

Please complete a declaration of competing interests, considering the following questions:

- Have you in the past five years received reimbursements, fees, funding, or salary from an organisation that may in any way gain or lose financially from the publication of this manuscript, either now or in the future?
- Do you hold any stocks or shares in an organisation that may in any way gain or lose financially from the publication of this manuscript, either now or in the future?
- Do you hold or are you currently applying for any patents relating to the content of the manuscript?
- Have you received reimbursements, fees, funding, or salary from an organization that holds or has applied for patents relating to the content of the manuscript?
- Do you have any other financial competing interests?
- Do you have any non-financial competing interests in relation to this paper?

If you can answer no to all of the above, write 'I declare that I have no competing interests' below. If your reply is yes to any, please give details below.

I declare that I have no competing interests.

I agree to the open peer review policy of the journal. I understand that my name will be included on my report to the authors and, if the manuscript is accepted for publication, my named report including any attachments I upload will be posted on the website along with the authors' responses. I agree for my report to be made available under an Open Access Creative Commons CC-BY license (http://creativecommons.org/licenses/by/4.0/). I understand that any comments which I do not wish to be included in my named report can be included as confidential comments to the editors, which will not be published.

Choose an item.

To further support our reviewers, we have joined with Publons, where you can gain additional credit to further highlight your hard work (see: https://publons.com/journal/530/gigascience). On publication of this paper, your review will be automatically added to Publons, you can then choose whether or not to claim your Publons credit. I understand this statement.

Yes Choose an item.
